# Genome sequence and analysis of methylotrophic yeast Hansenula polymorpha DL1

**DOI:** 10.1186/1471-2164-14-837

**Published:** 2013-11-27

**Authors:** Nikolai V Ravin, Michael A Eldarov, Vitaly V Kadnikov, Alexey V Beletsky, Jessica Schneider, Eugenia S Mardanova, Elena M Smekalova, Maria I Zvereva, Olga A Dontsova, Andrey V Mardanov, Konstantin G Skryabin

**Affiliations:** 1 Centre “Bioengineering” of RAS, Prosp. 60-let Oktyabrya, bld. 7-1, Moscow 117312, Russia; 2 Faculty of Chemistry, Lomonosov Moscow State University, 119999 Moscow, Russia and Belozersky Institute, Moscow State University, Leninskie Gory 1, Bldg. 40, 119991 Moscow, Russia; 3 Institute for Bioinformatics, Center for Biotechnology, Bielefeld University, Universitätsstraße 25, 33615 Bielefeld, Germany

**Keywords:** *Hansenula polymorpha*, Genome, Methylotrophic yeasts, RNA-seq, Yeast evolution

## Abstract

**Background:**

*Hansenula polymorpha* DL1 is a methylotrophic yeast, widely used in fundamental studies of methanol metabolism, peroxisome biogenesis and function, and also as a microbial cell factory for production of recombinant proteins and metabolic engineering towards the goal of high temperature ethanol production.

**Results:**

We have sequenced the 9 Mbp *H. polymorpha* DL1 genome and performed whole-genome analysis for the *H. polymorpha* transcriptome obtained from both methanol- and glucose-grown cells. RNA-seq analysis revealed the complex and dynamic character of the *H. polymorpha* transcriptome under the two studied conditions, identified abundant and highly unregulated expression of 40% of the genome in methanol grown cells, and revealed alternative splicing events. We have identified subtelomerically biased protein families in *H. polymorpha*, clusters of LTR elements at G + C-poor chromosomal loci in the middle of each of the seven *H. polymorpha* chromosomes, and established the evolutionary position of *H. polymorpha* DL1 within a separate yeast clade together with the methylotrophic yeast *Pichia pastoris* and the non-methylotrophic yeast *Dekkera bruxellensis*. Intergenome comparisons uncovered extensive gene order reshuffling between the three yeast genomes. Phylogenetic analyses enabled us to reveal patterns of evolution of methylotrophy in yeasts and filamentous fungi.

**Conclusions:**

Our results open new opportunities for in-depth understanding of many aspects of *H. polymorpha* life cycle, physiology and metabolism as well as genome evolution in methylotrophic yeasts and may lead to novel improvements toward the application of *H. polymorpha* DL-1 as a microbial cell factory.

## Background

Yeast capable of using methanol as their sole carbon and energy source have been described in several lineages [1]. All methylotrophic yeasts share the same methanol utilization pathway composed of abundant and highly inducible enzymes, localized in peroxisomes, which proliferate extensively upon growth in methanol [2,3]. The efficient and tightly regulated promoters of the methanol-assimilating genes are widely used in gene expression and recombinant protein production studies, and powerful industrial protein production platforms have been developed for several methylotrophic yeast species, namely, *Pichia pastoris, Hansenula polymorpha* and *Candida boindii*[4-9]. Methylotrophic yeasts are also widely used in studies of peroxisome biogenesis, protein targeting and function [10-13]. Due to their widespread application as cell factories and in basic research, methylotrophic yeasts are currently the subjects of intense genomic and systems biology studies.

For *Pichia pastoris*, now reclassified as *Komagatella pastoris, K. pseudopastoris* and *Komagatella phaffi*[14], draft or near complete genome sequences are available for several strains [15-17]. These achievements have greatly facilitated subsequent transcriptomic, proteomic and systems biology developments (see, for instance [18-20]).

Genomic and post-genomic studies in another popular and widely used methylotrophic yeast species, *Hansenula polymorpha*, somewhat lag behind those in *P. pastoris*. The *H. polymorpha* species complex in fact includes several phylogenetically distinct strains [21] now reclassified as *Ogataea polymorpha* and *Ogataea parapolymorpha*[22,23]. A genome sequencing project for strain *H. polymorpha* CBS4732 that resulted in assembly of about 90% of the genome, including the vast majority of encoded proteins [24], appears extremely useful for comparative genomic and proteomic studies [21,25], identification of various transcription responses [26] and studies of mechanisms of strain adaptation to growth on methanol [27].

Another widely used and popular *H. polymorpha* strain that has several advantages as protein production host is DL-1 also known as ATCC 26012 [21]. This strain is phylogenetically distinct from the majority of the *Ogataea* species complex [28] and is currently classified as *Ogataea parapolymorpha* DL-1 [23]. Such characteristics as resistance to heavy metals, oxidative stress, and thermotolerance also make the DL-1 strain an attractive host for various metabolic engineering purposes, for instance for development of novel ethanol producers [29].

We present here the almost complete genome of *H. polymorpha* DL-1 (ATCC26012), which enabled us to perform detailed analysis of genome content and organization, and identify shared and distinctive features with genomes of other methylotrophic yeast species. The presented genome sequence should bridge the gap in *H. polymorpha* genomic studies and facilitate further “omics” developments.

## Results and discussion

### Genome sequence, assembly and annotation

The whole genome of *H. polymorpha* DL-1 was sequenced by a pyrosequencing approach using a combination of shotgun and paired ends genome libraries and gap closure by selected PCR fragments sequenced on ABI 3730. Sequencing of the shotgun library resulted in the generation about 424 Mb of sequences with an average read length of 326 bp. Sequencing of the paired ends library produced 142896 reads. A total of 111 contigs assembled into 13 scaffolds were obtained. A near complete genome sequence was produced upon the generation and sequencing of appropriate bridge PCR fragments on an ABI 3730 sequencer (Applied Biosystems, USA). In addition, a single 41719-bp contig was identified as representing the mtDNA on the basis of very high coverage and extensive sequence similarity to known yeast mitochondrial genomes. The assembled sequence for the *H. polymorpha* DL-1 genome was deposited in the GenBank database under the accession nos. AEOI02000000 (nuclear genome) and HQ616673 (mtDNA).

The essentially complete genomic sequence of *H. polymorpha* DL-1 is thus composed of seven linear chromosomes ranging in size from 0.99 to 1.52 Mbp. Chromosomes 2, 3, 5, 6 and 7 correspond to particular contigs. Chromosome 4 was assembled as two contigs separated by an approximately 4 kb repeat-rich gap which we were unable to close. Another separate contig corresponds to a 7.7 kb rDNA locus, located within chromosome 1 and repeated about 25 times as estimated from its coverage. Chromosome 1 was therefore assembled as a scaffold of three contigs. The total calculated nuclear genome size of strain DL-1 is thus about 9 Mbp. The 42 kbp circular-mapping mitochondrial genome, identified as a separate contig, was characterized by us previously [30]. Details of the genome assembly statistics are provided in Table 1.

**Table 1:** **General features of *H. polymorpha *** **nuclear genome**

**Chromosome**	**Contigs (bp)**	**Total calculated length (bp)**	**GC content (%)**
1	297,310 + 7, 737(×25) + 650, 519	1, 141, 254	48.9
2	990, 963	990, 963	48.2
3	1, 273, 462	1, 273, 462	49.0
4	366, 734 + 922, 894	1, 289, 628*	47.7
5	1, 330, 267	1, 330, 267	48.3
6	1, 514, 933	1, 514, 933	46.7
7	1, 515, 570	1, 515, 570	46.4
Total		9, 056, 077	47.8

*gap between two contigs not taken into account.

A total of 5325 protein coding genes were predicted using Augustus trained on the assembled transcripts. tRNA genes were predicted using the tRNA-scanSE tool. Predicted gene models were used to assign functions, EC numbers and map GO terms using the RAPYD functional prediction pipeline. An overview of the statistics of the genome-wide functional annotation is provided in Table 2.

**Table 2:** Genes and functional annotation

**Coding sequences (% of total)**	**84.4**
Average gene length (bp)	1416
Average exon frequency	1.09
Average exon length	1289
Average intron length	65
rRNA genes	3 (× 25)
tRNA genes	80
Protein-coding genes	5325
Proteins with GO terms	2396
Proteins with EC numbers	1041

### Phylogenetic position of H. polymorpha DL-1

**Figure 1:**
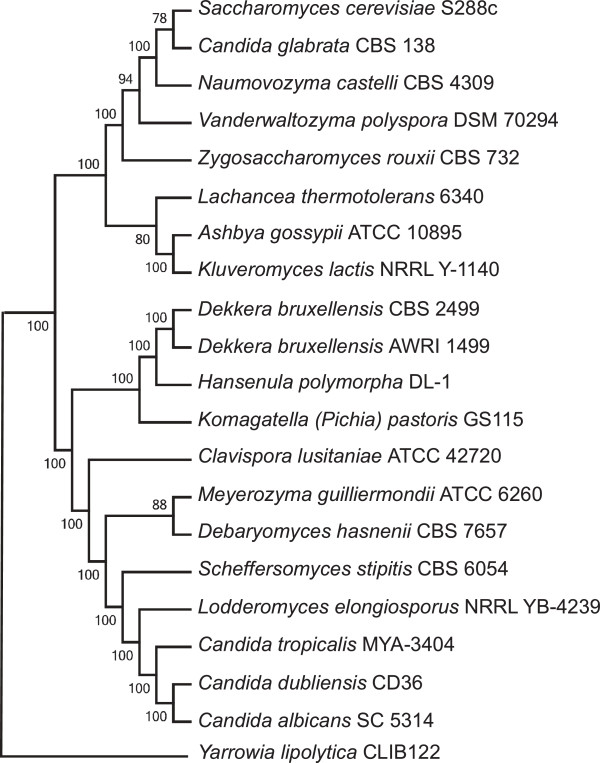
**Phylogenetic position of *H. polymorpha *within Hemiascomycetes.** The tree is based on Maximum Likelihood analysis of a concatenated alignment of 153 universally distributed orthologs previously identified in 42 sequenced fungal genomes. All nodes received the highest support in terms of approximate likelihood ratio tests, with a bootstrap analysis of 100 replicas.

We have previously reported the phylogeny of strain DL-1 based on comparisons of mitochondrial proteins [30]. The deduced phylogenetic position placed *H. polymorpha* DL-1 together with *Dekkera/Brettanomyces* group in a separate lineage, branching between the WGD and CTG groups with high bootstrap support values. This taxonomy is now confirmed by comparing nuclear encoded gene sets. *H. polymorpha* is grouped with *P. pastoris* and *Dekkera bruxellensis* (Figure 1) in a separate clade, whose ancestry apparently was not affected by such major events in the evolution of *Saccharomycetales* as a whole-genome duplication and genetic code alteration. A phylogenetic analysis of *D. bruxellensis* AWRI1499 gave similar results [31].

### Telomeres and subtelomeric regions

Yeast telomeres are dynamic structures fulfilling many functions in the cell [32]. Besides telomere repeats *per se*, linear eukaryotic chromosome ends in general possess highly variable repeated sequences adjacent to the telomeres. Proximal to the telomeres are the so-called subtelomeric regions, repeat-rich and gene-poor chromosome loci.

Several telomeric fragments from strain DL-1 have been isolated and cloned by Song and co-workers [33]. Sequence analysis of these fragments revealed the presence of (GGTGGCGG) telomeric repeats, sites of potentially bent DNA, and ARS sequences. All these fragments were found in our assembly at the utmost ends of the assembled contigs, along with the (GGTGGCGG) telomeric repeat sequence present at the assembled ends of chromosomes 4 and 7. The ARS consensus sequence, however, was present only at 3 chromosome ends; thus, the suggested core sequence hardly corresponds to authentic chromosomal replication origins, and is likely to be similar to the X-elements characteristic of *S. cerevisiae* telomeres [34].

A recent comparative genomic analysis of genes located at subtelomeric regions of evolutionarily diverse yeast species uncovered the extraordinary dynamics of subtelomeric gene families [35]. It was shown that genes residing near the telomeres undergo frequent recombination and duplication, which may allow evolutionary adaptation and innovation. The textbook case is exemplified by genetic variation in the subtelomeric MAL, MEL and SUC genes in *Saccharomycetacea*[35-37].

To investigate what genes are specifically enriched or depleted in *H. polymorpha* DL-1 subtelomeric regions we searched the genes located within 50 kbp from the chromosome ends and looked at their distribution into different functional categories. It appeared that different metabolic genes, various permeases and transporters responsible for metal, amino acid, and carbohydrate uptake, redox-processes and NADPH regeneration are overrepresented in *H. polymorpha* subtelomeric regions. GO enrichment analysis with Fisher exact test (FDR < 0,25) confirmed this observation, and additionally indicated more abundant representation of genes with oxidoreductase activity, cellular response to nitrogen starvation and extracellular stimuli, cellular response to nutrient and nitrogen levels, secondary metabolism, and abiotic stresses (Additional file 1: Figures S1-S5).

**Figure 2:**
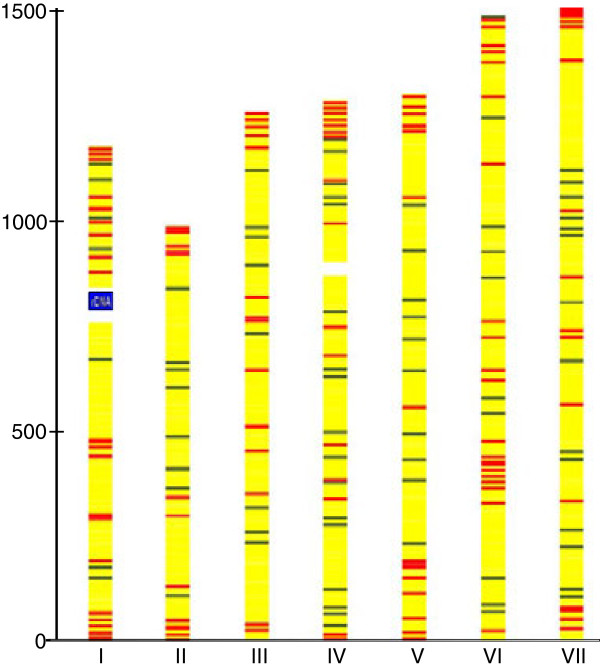
**Subtelomerically-biased and unbiased distribution of members of two multigene families across the *H. polymorpha *genome.** The subtelomeric family is represented by MFS-transporters (115 copies per genome, red), and the nonsubtelomeric family by proteins with armadillo-like domains (68 copies per genome, green).

The most abundant group of *H. polymorpha* genes with a predominantly subtelometic location is the one coding for MFS membrane transporters. Among 115 MFS genes present in the *H. polymorpha* genome, 40 are located in subtelomeric regions (Figure 2). Phylogenetic analysis of *H. polymorpha* MFS proteins showed clustering of subtelomerically located genes, providing support for their spread due to inter-and intrachromosomal recombination and amplification (Additional file 1:Figure S6).

### Transcriptomic analysis overview

The growth of methylotrophic yeast on methanol generates a strong environmental stimulus that dramatically affects numerous aspects of yeast cell metabolism, physiology, intracellular architecture, macromolecular synthesis, energetics and gene expression [38,39]. Quantitative analysis performed on the *H. polymorpha* strain NCYC495 *leu*^-^ using DNA microarrays [27] showed that hundreds of genes alter their expression in methanol-grown versus glucose-grown conditions. The methanol-induced gene set includes those encoding specific methanol utilization pathway enzymes, genes required for peroxisome biogenesis and function, stress response and antioxidant defence, mitochondrial respiratory function and numerous other genes belonging to diverse functional categories [25].

Our analysis based on RNA-seq complements and extends these data. RNA-seq is a powerful approach to global transcriptome analysis and has distinct advantages over micrioarray technology. RNA-seq is an efficient tool for quantification of gene expression, and may also be applied to the identification of novel transcripts and alternative splice sites [40-42]. Since the original demonstration of the many advantages of RNA-seq for characterization of *S. cerevisiae* transcriptome [43], this technology has been applied successfully for whole-genome transcriptome analysis of diverse yeast species [44-46], including *Pichia pastoris* GS115.

We used RNA-seq to characterize *H. polymorpha* DL-1 transcriptomes obtained from cells grown with glucose or methanol. The obtained data enabled us to identify over-and under expressed genes, quantify differential gene expression under the two conditions, and correct automatic annotation. In total, we obtained 733,393 pyrosequencing reads for samples cultivated with methanol and 709,815 reads for samples cultivated with glucose as the substrate. Of the total reads, 94.13% and 95.01% were mapped to the *H. polymorpha* genome, of which 89.94% and 88.46% were mapped to known exons, for samples grown on methanol and glucose, respectively. To quantify gene expression levels, the number of reads per total number of mapped reads was calculated for each sample.

The results of the transcriptome sequencing were explored to quantitatively analyse differential gene expression in *H. polymorpha* cells cultivated on methanol and glucose. A value characterizing differential expression level was considered as log2 of the ratio between the gene expression levels on methanol and glucose. A total of 5325 genes were annotated in the *H. polymorpha* genome. No expression was observed for 87 genes, while 2312 genes were up-regulated on methanol relative to glucose and 968 genes were down-regulated (with at least a two-fold difference in expression).

**Figure 3:**
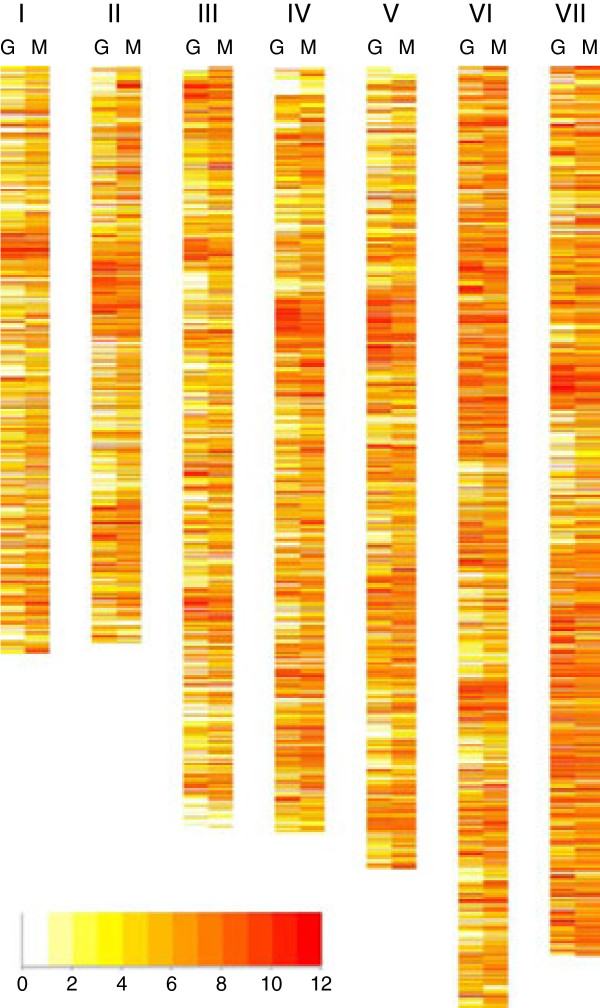
**Transcriptional landscape of the *H. polymorpha *genome.** Heat map showing chromosomal distribution of transcribed genes coloured according to expression levels (log2 tag count). G – glucose grown cells, M - methanol-grown cells. The colour code for normalized log2 transcript count is shown in the bar below.

The genome-wide landscape of the *H. polymorpha* transcriptome obtained from glucose grown cells is a variegated picture composed of “peaks” of over-expressed genes separated by “valleys” of genes with moderate or low-expression levels (Figure 3, lanes “G”). Some over-expressed genes tend to form clusters, and short transcriptionally “cold” regions are visible near telomeres. We could detect transcripts corresponding to 4652 genes, indicating that more than 87% of protein coding sequences are expressed as polyandenylated mRNA under these conditions. The “silent” portion of the genome included about 673 genes encoding many poorly characterized proteins. Genes for “hypothetical protein”, “putative secreted protein”, or “uncharacterized protein” constitute about 46% of this group. About 29% of genes among the “silent group” show significant (more than 10-fold) up-regulation in cells grown with methanol.

Genes abundantly expressed on glucose mostly perform housekeeping functions in different metabolic processes, ribosome biogenesis, translation, DNA repair, cell cycle and transcriptional regulation. Gene ontology (GO) analysis showed that this group is enriched for genes involved in such biological processes as generation of precursor metabolites and energy, gene expression, translation, cellular biosynthetic process, macromolecule biosynthetic process, macromolecule metabolic process and other anabolic activities (Additional file 1: Figures S7-S10). The encoded proteins show predominantly cytoplasmic, cell wall, mitochondrial and ribosomal localizations.

Changing the carbon source from glucose to methanol dramatically altered the observed patterns and chromosomal landscape of the *H. polymorpha* transcriptome (Figure 3, lanes “M”). The distribution of genes between different GO categories as related to expression level differed from what was observed for glucose grown cells. A large portion of the genome was expressed (94%), and more genes with medium and high expression levels (normalized log2 count above 4) were detected (Figure 3).

Genes highly expressed in methanol of course include those encoding methanol metabolic enzymes as well as hundreds of genes for proteins involved in numerous other functional categories responsible for complex methanol adaptation reactions, including peroxisome biogenesis and function, antioxidant defence, pentose phosphate pathway, various transporters, some ribosomal proteins and components of the mitochondrial oxidative phosphorylation system (Additional file 1: Figures S11-S18). GO enrichment analysis for the group of genes overexpressed in methanol shows a prevalence of such terms as “protein catabolic process”, “cellular macromolecule catabolic process”, “energy derivation by oxidation of organic compounds”, “response to stimulus”, “response to stress”, “oxidation/reduction”, “cell periphery”, and “microbody, peroxisome”. Among the genes up-regulated in methanol are those involved in oxidoreductase and transporter activities.

Genes down-regulated in methanol, as noted in previous microarray studies, include those for glycolytic enzymes and also genes for various anabolic and macromolecule biosynthetic processes (transcription, translation, DNA replication), kinase and phosphotransferase activities (Additional file 1: Figures S19-S22). This reduction in *H. polymorpha* biosynthetic activity is related to a general reduction in proliferation in the course of growth on a C1 compound.

**Figure 4:**
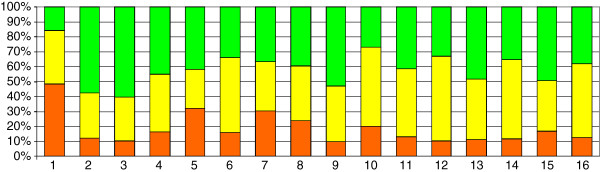
**Methanol up-regulation and down-regulation of *H. polymorpha *genes distributed to various KEGG categories.** Metabolism: 1 - Carbohydrate metabolism, 2 - Energy metabolism, 3 - Lipid metabolism, 4 - Nucleotide metabolism, 5 -Amino acid metabolism, 6 - Glycan biosynthesis and metabolism, 7 - Metabolism of cofactors and vitamins, 8 - Biosynthesis of other secondary metabolites, 9 - Xenobiotic biodegradation and metabolism. Genetic Information Processing: 10 - Transcription, 11 - Translation, 12 - Folding, sorting and degradation, 13 - Replication and repair. Environmental Information Processing: 14 - Signal transduction. Cellular Processes: 15 - Transport and catabolism, 16 - Cell growth and death. Fractions of methanol-induced genes are shown in green, glucose-induced genes in red, and genes without significant changes in expression in yellow.

In order to obtain a more integrated view of the patterns of *H. polymorpha* differential gene expression, we analysed the expression levels of genes functionally subdivided into KEGG groups and categories. In this analysis each gene may be classified into one or more groups, depending on its function (Additional file 2: Table S1). The percentages given in Figure 4 indicate the proportions of genes that are up-regulated, down-regulated, or have the same expression level during growth in glucose and methanol. As expected, genes involved in carbohydrate metabolism are mainly down-regulated during growth in methanol; 49% of these genes have an increased expression level on glucose, while only 16% of the genes show an increased expression level in methanol. The opposite situation is observed for the genes involved in energy metabolism, i.e., 12% and 58% of the genes are down- and up-regulated during growth in methanol compared to glucose, respectively. Generally, among the genes involved in metabolism, 20% are down-regulated and 39% are up-regulated in methanol. Most of the other KEGG groups comprised more genes up-regulated in methanol than in glucose (Figure 4).

Another characteristic feature of the *H. polymorpha* transcriptome revealed by this analysis is the presence of paralogous copies of housekeeping genes, involved in various metabolic, biosynthetic and cellular processes that are specifically expressed when cells are grown on either glucose or on methanol.

In order to validate RNA-seq data we performed qPCR analysis for three genes, with characteristic levels of differential expression under two conditions. The HPODL_02458 gene encoding superoxide dismutase is upregulated on methanol, the HPODL_01177 gene encoding glutathione reductase showed constitutive expression, and HPODL_01513 gene encoding pyruvate kinase is downregulated. The same cDNA samples used for RNA-seq experiments, and biological replicates (cDNAs from two cultures grown in glucose and two cultures grown in methanol) were analysed by qPCR. Very good correlation between RNAseq and qPCR data was observed (Additional file 3).

A more detail analysis of specific patterns of differential gene expression on the two carbon sources is provided in the sections below.

### Regulation of glucose metabolism

Glycolysis is the central pathway for carbohydrate metabolism in yeasts. Under conditions of glucose starvation (methanol grown cells) glycolytic enzymes have to catalyse reverse reactions of gluconeogenesis [47]. Expression levels of the majority of glycolytic enzyme genes do not change significantly between the two studied conditions (Additional file 2: Table S2). Glucokinase functions only in the direction of glycolysis and is down-regulated in methanol. Two genes from the “preparatory phase”, fructose bisphosphate aldolase and triosephosphate isomerase, responsible for the entry of two products of methanol metabolism, dihydroxyacetone and glyceraldehyde-3-phosphate, into the glycolytic pathway, are up-regulated in methanol. Also up-regulated is the “gluconeogenic” fructose-1,6-bisphosphatase.

A moderate increase in expression of genes from the “Pay-off phase”, namely glyceraldehyde-3-phosphate dehydrogenase, phosphoglycerate kinase, phosphoglycerate mutase, enolase, and pyruvate kinase was observed in glucose-grown cells. The expression of pyruvate metabolic enzymes shows multidirectional trends – while levels of pyruvate carboxylase and phosphoenolpyruvate carboxykinase are mostly unchanged, the level of pyruvate decarboxylase drops about 2-fold in methanol.

*H. polymorpha* is attractive cell factory for high-temperature ethanol production [29,48,49]. Cytosolic alcohol dehydrogenase (ADH), the key ethanologenic enzyme, is one of the most abundantly expressed proteins both in glucose and methanol grown cells. Expression of the two ADH genes vary – in contrast to the major ADH gene, that is slightly induced on methanol, the minor gene is induced about 10 fold in methanol-grown cells (Additional file 2: Table S2).

The balance between alcoholic fermentation and respiration is partially controlled by enzymes of pyruvate metabolism. The levels of key pyruvate metabolic genes differ in two conditions. While the two pyruvate dehydrogenase isoforms are expressed constitutively, pyruvate decarboxylase is slightly repressed on methanol. Up-regulated on methanol is the gene for major acetyl-coenzyme A synthetase subunit. Altogether these data justify upregulation of pyruvate dehydrogenase bypass in methanol-grown cells.

### Regulation of methanol metabolism

**Figure 5:**
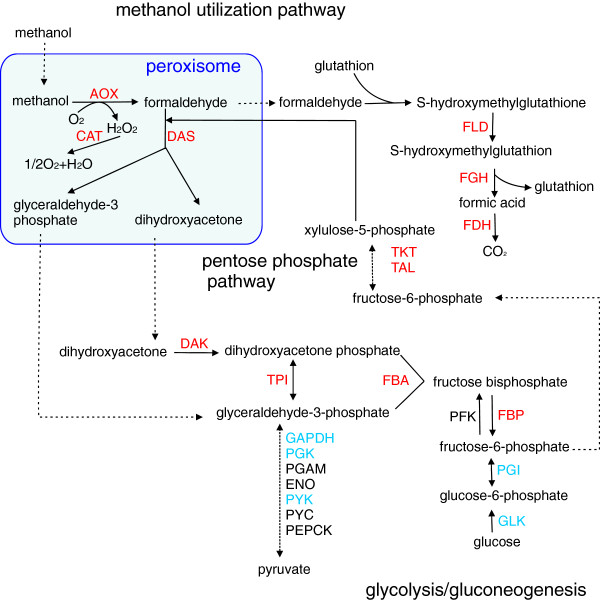
**Overview of methanol utilization and related metabolic processes in *H. polymorpha*.** Methanol- and glucose-induced genes are shown in red and blue, respectively. Enzyme abbreviations: AOX, alcohol oxidase; CAT, catalase; DAS, dihydroxyacetone synthase; DAK, dihydroxyacetone kinase; FLD, formaldehyde dehydrogenase; FGH, S-formyl glutathione hydrolase; FDH, formate dehydrogenase; GLK, glucokinase; PGI, glucose-6-phosphate isomerase; PFK, phosphofructokinase; FBP, fructose-1,6-bisphosphatase; FBA, fructose-bisphosphate aldolase; TPI, triosephosphate isomerase; GAPDH, glyceraldehyde-3-phosphate dehydrogenase; PGK, phosphoglycerate kinase; PGAM, phosphoglycerate mutase; ENO, enolase; PYK, pyruvate kinase; PYC, pyruvate carboxylase; PEPCK, phosphoenolpyruvate carboxykinase; TKT, transketolase; TAL, transaldolase.

The biochemistry, molecular genetics and enzymology of methanol utilization (MUT) in *H. polymorpha* and other methylotrophic yeasts have been well studied (reviewed in [2,38]). In the MUT pathway, peroxisomal alcohol oxidase (AOX), the first and most abundant among the enzymes of the pathway, oxidizes methanol to formaldehyde and hydrogen peroxide; the latter is broken down to oxygen and water by peroxisomal catalase. Formaldehyde is either fixed to xylulose 5-phosphate by the action of dihydroxyacetone synthase (DAS) or dissimilated in the cytosol to CO_2_ through glutathione-dependent formaldehyde dehydrogenase (FLD), S-formyl glutathione hydrolase (FGH) and formate dehydrogenase (FDH) (see Figure 5).

Genes involved in methanol metabolism are highly up-regulated. The magnitude of up-regulation varies from more than 10-fold for FDH to 4.88-fold for FLD (Additional file 2: Table S3). The obtained values are significantly higher than those reported using microarrays for *H. polymorpha* strain NCYC495 *leu*^*-*^[27]. These differences may be explained by strain characteristics, differences in cultivation conditions, or the higher sensitivity of RNA-seq as compared to hybridization methods [42].

### Pentose phosphate pathway

The pentose phosphate pathway (PPP) is important for methanol metabolism as a source of xylulose-5-phosphate (Xu5P) – a substrate of DAS for formaldehyde assimilation and further biosynthesis of sugars, nucleosides and amino acids. The generation of Xu5P via PPP involves the ATP dependent phosphorylation of dihydroxyacetone by dihydroxyacetone kinase (DAK) in the cytosol.

PPP is also required for the regeneration of NADPH, an important cofactor in redox metabolism. Genes for enzymes from the oxidative PPP phase, glucose-6-phosphate dehydrogenase, 6-phosphogluconolactonase, and 6-phosphogluconate dehydrogenase do not significantly alter their expression in methanol as compared to glucose-grown cells. In contrast, genes for enzymes from the non-oxidative phase, ribose-5-phosphate isomerase, ribulose-phosphate 3-epimerase, transketolase and transaldolase are significantly induced in methanol-grown cells. Levels of up-regulation vary from 1.92-fold (transketolase) to about 10-fold (ribose-5-phosphate isomerase). Most PPP genes are duplicated or triplicated and expression patterns of paralogous copies as compared to “master” copies show different trends (Additional file 2: Table S4). An overview of the expression patterns of key *H. polymorpha* methanol utilization and carbohydrate metabolism genes and the interrelationships of their respective pathways is shown in Figure 5.

### Peroxisome biogenesis, function and degradation

Peroxisomes are vital for methylotrophy, enabling adequate compartmentalization of formaldehyde assimilation and dissimilation pathways and providing a secure site to detoxify hydrogen peroxide and reactive oxygen species (ROS) generated in the course of oxidising methanol and other substrates [12]. *PEX* genes encode proteins, termed peroxins, required for the biogenesis and proliferation of peroxisomes [50]. Products of *PEX* genes form complexes that function cooperatively in the course of peroxisome biogenesis [51,52]. This cooperativity is partially reflected in the coordinated levels of up-regulation of PEX genes in the presence of methanol (Additional file 2: Table S5). Thus, the *PEX3* and *PEX19* genes, implicated in sorting newly synthesized peroxisomal membrane proteins to their target membrane [53], are up-regulated 3.63- and 2.84-fold, respectively.

Expression levels of the *PEX5* and *PEX7* genes, encoding PTS1 and PTS2 peroxisomal import receptors [50,54] vary. While *PEX5* is substantially induced by methanol (5.65-fold), *PEX7* is not up-regulated. This observation is consistent with a limited number of peroxisomal matrix proteins containing PTS2 receptors.

Most peroxins are involved in the transport of matrix proteins from the cytosol into the peroxisome lumen. The suggested docking and translocation complex involves ring finger proteins Pex2, Pex10 and Pex12 involved as ubiquitin ligases in receptor recycling, a dimeric Pex13p/Pex14p complex [55], linked by Pex8p, that also functions in the release of PTS1 cargo proteins from their receptor [56] and, probably, Pex17p [57]. All these genes are up-regulated in methanol. The Pex11p, Pex11cp, Pex23p, Pex24p, Pex25p, and Pex29p proteins are involved in peroxisome proliferation. These proteins are integral protein components of the peroxisomal membrane. The highest induction was observed for *PEX11* (6.94-fold). Peroxins implicated in recycling PTS receptors to the cytosol include Pex4p, Pex22p [58,59], Pex1p, Pex6p, and Pex26p [54]. The *PEX1* and *PEX6* genes are up-regulated more than 3-fold, while the *PEX4*, *PEX22*, and *PEX26* genes show modest up-regulation on methanol.

Our data shows that levels of up-regulation of *PEX* genes on methanol are higher than those reported earlier using microarrays and other approaches [27,60]. This difference, as noted above, may be explained by variations in cultivation conditions, sample preparation, or the known advantages of RNA-seq in sensitivity and dynamic range.

Peroxisome homeostasis is a balance between proliferation and degradation of these organelles [61]. Selective peroxisome removal in the vacuolar/lysosomal compartment (pexophagy) is mediated by components of the general autophagy core machinery [11,62].

In methylotrophic yeast pexophagy is induced upon change of carbon source (from methanol to glucose) and nitrogen starvation [63,64].

Pexophagy as other autophagic processes proceeds via a multistep pathway, controlled by about 30 genes, acting cooperatively and sequentially in autophagosome formation, vesicle fusion and vacuolar degradation [65,66].

Moderate increase in expression of ATG genes in methanol-grown cells was observed in the cited study of adaptation of *H. polymorpha* cells to methanol using microarray gene expression analysis [27]. Our results show more variation in ATG genes expression in methanol or glucose-grown cells (Additional file 2: Table S6). Thus, most significant downregulation on methanol was detected for ATG1 and ATG6 genes. ATG1 gene encodes serine/threonine kinase required for phagophore assembly site (PAS) formation, and ATG6 encodes subunit of phosphatidylinositol 3-kinase complexes, involved in autophagy and vacuolar protein sorting [67]. Upregulated on methanol were ATG17, ATG20, ATG21 genes. ATG17 encodes a regulatory subunit of ATG1 complex, and a scaffold for other ATG proteins during PAS organization, ATG20 and ATG21 encode sorting proteins required for vesicle formation in the cytoplasm-to-vacuole targeting (Cvt) pathway [67].

Significance of these observations requires further investigation. It should be noted, however, that we collected cells at the stage of rapid exponential growth, cells did not starve for carbon or nitrogen source, and these growth conditions should not be favorable for autophagy or pexophagy induction.

### Antioxidant system

Elimination of hydrogen peroxide and ROS generated in the course of methanol oxidation, oxidative phosphorylation and other metabolic processes is necessary in methylotrophic yeast cells to prevent irreversible oxidative damage to cell constituents. Peroxisomal catalase and peroxiredoxin Pmp20 are defensive enzymes required to protect the peroxisomal matrix and membranes from H_2_O_2_ and ROS [68,69]. These two genes are highly up-regulated (6.34- fold and 9.21-fold) in methanol.

ROS escaping from the peroxisomal defence system are detoxified by other enzymatic and non-enzymatic defence systems. The superoxide anion in yeast, as well as in other eukaryotes, is cleaved to H_2_O_2_ and O_2_ through the action of mitochondrially-located manganese superoxide dismutase (MnSod) and cytoplasmically-located copper-zinc superoxide dismutase [70-73]. Three *H. polymorpha* MnSod genes show marked up-regulation in methanol (Additional file 2: Table S7), while the Cu/Zn Sod, surprisingly, shows marked down-regulation.

The cytosolic thioredoxin and gluthathione-based defence system in *H. polymorpha* includes a number of genes, encoding two gluthathione biosynthetic enzymes, γ-glutamylcysteine synthetase and glutathione synthetase, glutathione peroxidase, glutathione reductase, multiple copies of glutaredoxin, glutathione S-transferase genes and paralogous pairs of thioredoxin and thioredoxin reductase genes. All these genes show variable but substantial induction in methanol, except glutathione peroxidase, which is down-regulated, and glutathione reductase and γ-glutamylcysteine synthetase, which did not change expression level during growth on methanol or glucose (Additional file 2: Table S6).

### β-oxidation of fatty acids

Fatty acid β-oxidation in yeast is restricted to peroxisomes [74]. Acyl-coenzyme A oxidase, a multifunctional enzyme, and 3-ketoacyl-CoA thiolase are involved in β-oxidation and their expression was induced in methanol (Additional file 2: Table S8). Besides these enzymes, other gene products are known to be required for efficient peroxisomal fatty acid oxidation. The list of these enzymes includes but is not limited to catalase, carnitine acetyltransferase, mitochondrial carnitine carrier protein, peroxisomal 2,4-dienoyl-CoA reductase, fatty acyl-CoA synthetase. Genes encoding all the proteins listed in Table S8 are significantly up-regulated in methanol.

### Transcription factors

Our current understanding of the mechanisms of methanol sensing and methanol –inducible gene expression in methylotrophic yeasts is far from being complete [38,39]. Several positive and negative cis-acting elements have been identified in the promoter regions of *P. pastoris*[75], *H. polymorpha*[76], *Candida boidinii*[77] MUT pathway genes . These elements are potential sites of interaction with trans-acting transcriptional regulators, activating or repressing transcription in methanol or glucose grown cells respectively. Genes encoding some of these factors, namely the *P. pastoris Mxr1* gene [75], *C. boidinii Trm1* and *Trm2* genes [78,79], *H. polymorpha Mpp1* gene [80], regulating both MUT and PEX genes transcription, were isolated and characterized.

To extend the list of candidate transcription factors controlling methanol-inducible gene expression in *H.polymorpha* we searched *H. polymorpha* annotated protein set for specific GO terms, like “sequence-specific DNA binding transcription factor activity”; “regulation of transcription, DNA-dependent” and analysed expression of corresponding genes in methanol and glucose grown cells. Orthologs of previously identified methanol-specific transcriptional regulators mentioned above were also included.

In the obtained dataset of 77 genes about half (thirty eight genes) were upregulated on methanol, twenty two genes did not change their expression and seventeen genes were downregulated in this conditions.

Among top 12 genes with highest level of upregulation ten genes are new and two genes encode previously known proteins (Additional file 2: Table S9) . One known gene is HPODL04601, encoding Mpp1 protein (upregulated 40-fold). Another gene is HPODL00650 – *H. polymorpha* ortholog of *P. pastoris Mxr1* gene, induced more than 80-fold on methanol. The patterns of regulation of the two orthologs differ, since in *P. pastoris* Mxr1p is constitutively expressed at low level and exerts its regulatory function by changing subcellular localization.

“Unknown” genes show upregulation levels ranging from 8-fold to more than 130-fold (Additional file 2: Table S9).

These genes are attractive targets for further genetic and biochemical investigation.

Among the downregulated genes are orthologs of *S. cerevisiae* transcription factors, regulating nitrogen metabolism (GCN4, GLN3), unfolded protein response (HAC1), several uncharacterized proteins.

It should be noted, that the level of induction *per se* cannot be considered as the sole criterion for identification of potential regulators. For instance, the *H. polymorpha* orthologs of *C. boidinii* Trm1 gene, a proposed master transcriptional regulator of methanol-specific gene activation, or *S. cerevisiae* CAT8 gene, encoding ADR1 coregulator, are only slightly induced on methanol.

Expression levels of *SWI/SNF* subunits of chromatin remodeling complex previously shown to play significant role in methanol-inducible gene expression [81] varied slightly (Data not shown).

### Specific metabolic features

Several metabolic traits are used in traditional taxonomic descriptions of *H. polymorpha* strains. Among these traits is the important ability to assimilate nitrate and nitrite as nitrogen source [82]. Distinct metabolic features of strain DL1 related to carbohydrate metabolism include the capability to utilise maltose, directed by the MAL gene cluster [83], and the capability to utilise xylose, arabinose and cellobiose. All the corresponding genes were identified in the genome and were found to be expressed at variable levels in glucose and methanol (Additional file 2: Table S10). A notable feature of the genetic control of xylose metabolism is the presence of paralogous copies of xylose reductase and xylitol dehydrogenase genes specifically expressed in either methanol or glucose.

### Alternative splicing sites

Alternative splicing (AS) is one of the major contributors towards proteome variation in higher eukaryotes. In yeast the role of AS in mRNA diversity is less significant since the majority of predicted genes do not harbour more than one exon. Intron frequency in *H. polymorpha* genome is also low, its 457 intron-containing genes corresponding to only 8.5% of the total protein coding genes. In contrast, in the *P. pastoris* genome 633 intron-containing genes constitute about 12% of all the protein-coding genes [15].

Ninety-four AS events detected in *H. polymorpha* based on RNA-seq analysis and computational predictions belong to the “retained intron” variant (Additional file 2: Table S11). We detected only one example of an alternative 3′-splice site (A3SS). In comparison, 270 AS events were reported in the *P. pastoris* genome, including 261 cases of a retained intron, two cases of an alternative 3′-splice site, four cases of an alternative 5′-splice site, and two cases of a skipped exon [60].

RNAseq data for selected AS event was validated by PCR analysis. For HPODL_03187 gene mapping of RNAseq reads indicated approximately equal levels of two transcripts – the correctly spliced variant and the variant with retained intron. The presence of the two transcripts with comparable abundance was detected by RT-PCR using exon-specific flanking primers (Additional file 4).

### General genomic features, transposons, genetic code

Several draft and near complete annotated genomes available for the *D. bruxellensis*[31,84], *P. pastoris*[15,16,18] and *H. polymorpha*[24] strains constitute a valuable resource for comparative genomics and were used by us for *ab initio* analysis of genomic changes related to the evolution of clade- and species-specific characteristics and traits in this subdivision of *Saccharomycotina*.

The general features of the *H. polymorpha* and *P. pastoris* genomes are rather similar (Table 3) and are close to those of “protoploid *Saccharomycetaceae*” [85], a group that did not experience ancestral whole-genome duplication. Chromosome numbers range between 4 and 7, genome size varies between 9 and 13 Mbp; they have about 5000 genes per genome, and few splicosomal introns. The *H. polymorpha* genome is denser (84% of the coding sequence as compared to 80% in *P. pastoris* and 72% in *D. bruxellensis*). Comparisons with the *D. bruxellensis* genome are more complicated, since published papers [31,84] indicate the complex heterozygous poliploid nature of the sequenced genomes for two strains, which are not complete.

**Table 3:** General features of compared yeast genomes

**Species**	**Chromosome number**	**Genome size (Mbp)**	**Average GC content**	**Total CDS number**	**Avreage CDS size (codons)**	**CDS/10 kbp**
*Dekkera bruxellensis* CBS 2499	unknown	13.39	39.9	5636	440	4.21
*Pichia pastoris* GS115	4	9.22	41.1	5040	476	5.46
*Hansenula polymorpha* DL1	7	9.06	47.8	5325	469	5.88

The *H. polymorpha* genome has markedly higher G + C content in non-coding and coding sequences – a feature that may be directly related to its thermotolerance. This difference is reflected in difference in codon usage between the three yeast species (Table S12). There is an obvious bias for codons having G or C at the second and third positions in the *H. polymorpha* genome as compared to *P. pastoris* and *D. bruxellensis*. This codon bias should be considered in designing synthetic genes for applications of *H. polymorpha* as a protein production host.

The nucleotide composition along *H. polymorpha* chromosomes is not uniform (Additional file 1: Figure S23) and extended (10–20 kbp) AT-rich regions can be identified in the middle of each chromosome. These AT-rich regions are mostly devoid of protein coding genes and thus could potentially correspond to centromeres [86], which until now have been only poorly characterized in yeast clades other than *Saccharomycetaceae*, where they are known to possess point centromeres with three characteristic conserved regions [87]. We found that in the *H. polymorpha* genome these AT-rich “centromeric” regions contain clusters of direct and inverted repeats of 290 bp “solo LTR elements” belonging to the Ty1/Copia group. In several cases these repeats are in the vicinity of “master” full-length Ty/Copia elements (Additional file 1: Figure S24). This invasion of Ty/Copia elements is an event specific for *H. polymorpha* DL-1, as revealed by a comparison of the set of repetitive elements in *H. polymorpha* and *P. pastoris* genome (Additional file 2: Table S13). About half of *H. polymorpha’s* Ty/Copia sequences are located in these regions. Thus, similar to *Debarymoyces hansenii* and other CTG yeast members [86], *H. polymorpha* centromeres are likely unique for each chromosome and marked by clusters of LTR-sequences.

All three species harbour mating type loci with a very similar organization (Additional file 1: Figure S25). In addition to the “main” MAT locus, identical in structure to the previously reported MAT locus from the *H. polymorpha* strain CBS4732 [88], strain DL-1 possesses a probable “silent” inverted copy of the MATa2 gene. This copy is located 20 kbp away from the main locus on chromosome 4 and is flanked by inverted repeat of the SLA2 gene, a conserved gene found adjacent to MAT loci in many yeasts and fungi.

### Genome redundancy, gene duplications

The rate of genome redundancy in “non-WGD” species is usually lower than in the “post-WGD” group. Still, up to 34% of their genome may be occupied by ancestral dispersed and tandem duplications [85]. Following the approach previously used to estimate the overall rate of genome duplication in *D. bruxellensis*[84], we calculated the number of segmental duplications in the *H. polymorpha* DL-1 and *P. pastoris -* GS115 genomes. The performed analysis (Additional file 2: Table S14) shows that the level of segmental duplications in the *H. polymorpha* and *P. pastoris* genomes are comparable and lie within the level of non-WGD species. Figures obtained for *D. bruxellensis* were higher than reported before and reflect the heterozygous nature of the CBS2499 genome [89].

To estimate genome redundancy at the protein-coding level the predicted proteome was analysed using the OrthoMCL server [90]. This approach yielded the most extensive classification of predicted proteins compared to other annotation methods and was independent from functional annotation. From the 4833 identified clusters 3762 were unique, and 1071 paralogs (22%) were distributed in families containing from 2 to 9 members (Additional file 1: Figure S26). The “raw genome redundancy”, identified as the ratio of the total number of protein genes (5325) versus the total number of unique protein families (4217) in *H. polymorpha* DL-1 was 1.26 - a value similar to that calculated for “protoploid *Saccharomycetaceae*” [85] (from 1.2 to 1.3).

### Comparative gene content

**Figure 6:**
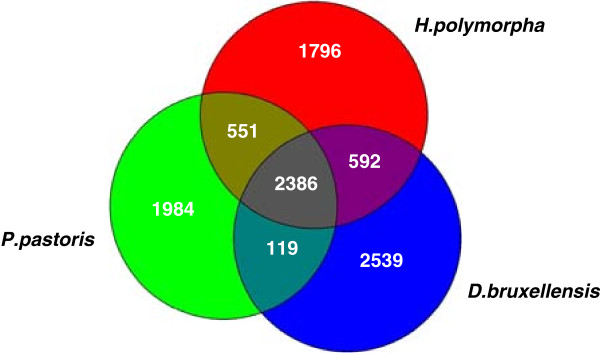
Distribution of shared and unique protein families in 3 yeast genomes.

The predicted *H. polymorpha DL-1*, *D. bruxellensis* CBS2499 and *P. pastoris* GS115 proteomes were subjected to comparative analysis with EDGAR [91] to identify “core gene set” and species-specific paralogous gene sets and expanded protein families as related to the evolutionary history and life style of compared yeasts. The distribution of shared and unique proteins in the three genomes is summarized in Figure 6. Though the *D. bruxellensis* genome is not complete, it is evident that the number of orthologous pairs in *H. polymorpha* and *D. bruxellensis* (2978) is higher for *H. polymorpha* and *P. pastoris* (2937). Common to the three species is the 2386 “core set”, and about half of each species’ proteome is represented by unique paralogs. The actual difference between the three proteomes, however, may be not so dramatic, since the majority of species-specific proteins fall in categories like “hypothetical protein”, “uncharacterized/unnamed protein”, “putative protein of unknown function” etc. The list of characteristic abundant species-specific paralogous protein families is shown in Table S15.

In order to evaluate the degree of sequence variation between the three genomes we performed a pairwise BLAST comparison of all shared orthologous genes for all possible genome pairs. Thus, the established degree of sequence variation between *H. polymorpha* and *D. bruxellensis* genomes is 52.2%, between the *H. polymorpha* and *P. pastoris* genomes it is 49%, and between *D. bruxellensis* and *P. pastoris* it is 47.3%. These values are typical of the genera-level divergence observed between yeast species belonging to other lineages [92]. It is thought that this high-level sequence variability, accompanied by conservation of many yeast-type physiological and morphological traits, is due to stochastic genetic drift, characteristic of the evolution of unicellular *Saccharomycotina* species [85,92].

Synteny between the H. polymorpha, D. bruxellensis and P. pastoris genomes.

**Figure 7:**
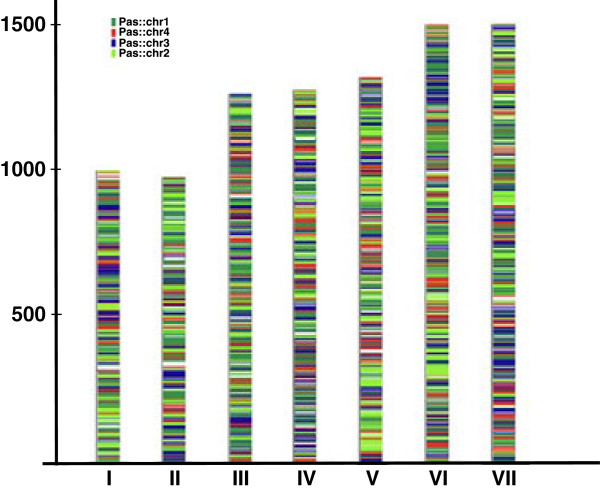
**Distribution of syntenic regions between *P. pastoris *** **chromosomes 1–4 and *H. polymorpha *** **chromosomes.**

The established rate of sequence divergence between the *H. polymorpha*, *D. bruxellensis* and *P. pastoris* genomes (see above) excludes expectations of the existence of extended syntenic regions between the three genomes. In other yeast lineages this level of sequence divergence is usually accompanied by extensive chromosomal rearrangements, leaving rather short recognizable syntenic blocks, though of course sequence divergence and synteny conservation are two independent measures of genetic distance [92]. In accordance with this we found significant gene reshuffling between the *P. pastoris* and *H. polymorpha* genomes (Figure 7). Application of a similar type of analysis towards the *D. bruxellensis* genome is complicated since both available genomic sequences are currently represented by multiple contigs and scaffolds [31,84].

Therefore, to gain a global view of the extent of synteny conservation between the three genomes we have used whole-genome dot-plot comparisons that are less sensitive to the quality of a genomic assembly. The obtained data (Additional file 1: Figure S27) shows that the *D. bruxellensis* and *H. polymorpha* genomes share a higher degree of synteny conservation relative to the *P. pastoris* and *H. polymorpha* pair. This notion was further confirmed in the course of an analysis of gene order in chromosomal loci encompassing methanol utilization pathway enzymes in the three yeast species (see below).

### Genome comparison reveals patterns of evolution in MUT-pathway genes

Phylogenetic analysis as well as estimation of the rate of synteny conservation clearly shows that *H. polymorpha*, a methylotrophic yeast, is phylogenetically closer to the non-methylotrophic *D. bruxellensis* than to the methylotrophic species *P. pastoris*. This observation prompted us to investigate more closely the molecular basis of the “MUT-plus” and “MUT-minus” genotypes in these yeasts and to look at the genomic status of MUT pathway genes in the three species. To achieve this goal we checked the two available *D. bruxellensis* genomes for the presence of genes encoding known MUT pathway enzymes and performed a comparative gene order analysis of extended *H. polymorpha* chromosomal loci surrounding several of these genes.

**Figure 8:**
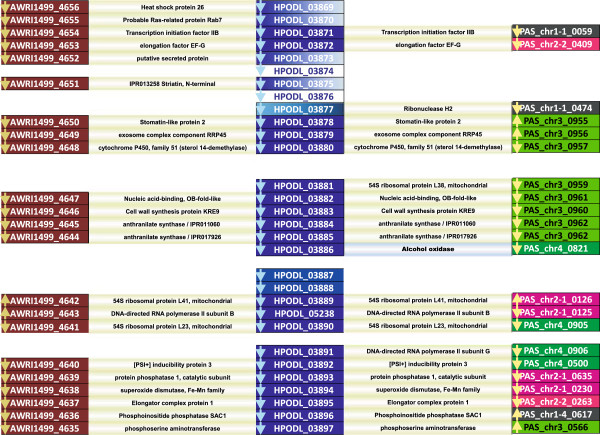
**Synteny maps around the *H. polymorpha *MOX gene and corresponding regions in the genomes of *D. bruxellensis *and *P. pastoris *GS115. ***H. polymorpha* genes (HPODL) are shown in dark blue, *D. bruxellensis* genes (AWRI) in purple, *P. pastoris* genes (PAS) are coloured depending on their chromosomal origin (chr1- black, chr2- pink, chr3- light green, chr4- dark green). Orthologous genes are connected with lines showing qualifiers taken from corresponding annotations. Gene names correspond to GenBank nomenclature.

For the *H. polymorpha* MOX gene, encoding the first enzyme in the pathway, we immediately obtained a striking result, showing a high degree of synteny conservation between the *H. polymorpha* “MOX locus” and orthologous loci in the genomes of two *D. bruxellensis* species, with a clear gap at the position of the MOX gene itself and a short adjacent region, indicating a gene loss event (Figure 8). The *P. pastoris* genome displayed a less pronounced degree of gene order conservation in the compared loci. Detected synteny breaks included a clear chromosome rearrangement event leading to apparent relocation of the AOX gene from the extended syntenic block on *P. pastoris* chromosome 3 to *P. pastoris* chromosome 4.

The higher degree of synteny conservation in the *D. bruxellensis* and *H. polymorpha* genomes as compared to the *P. pastoris*/*H. polymorpha* pair is also evident from a gene order comparison of chromosomal loci encompassing genes for other MUT pathway enzymes, namely the formaldehyde dehydrogenase (FLD), formate dehydrogenase (FDH) and dihydroxyacetone synthase (DAS) genes (Figure S28). Apparently functional copies of all these genes are present in the *D. bruxellensis* genome, imposing an important question about their possible metabolic roles in the absence of the “upstream” MOX gene.

From this comparison it also became clear that the capacity for methanol utilization could be lost in a particular yeast lineage due to a simple chromosomal deletion event, without obvious effects on strain viability.

**Figure 9:**
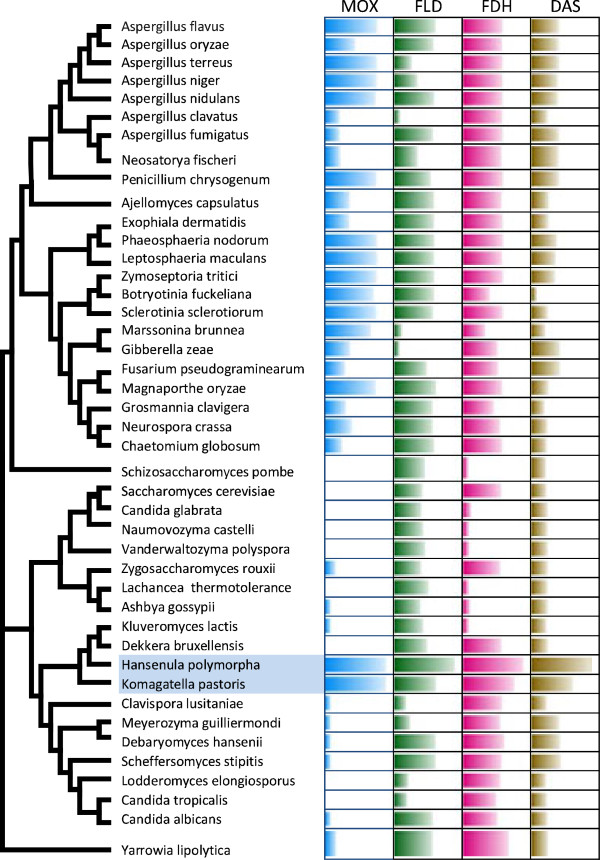
**Distribution and similarity of MUT pathway proteins in Ascomycetes.** On the left is a tree of *Saccharomycotina* and *Pezizomycotina* species with known genomes, based on a comparison of concatenated sequences of 153 proteins. On the right is a table showing the presence of orthologs of *H. polymorpha* methanol oxidase (MOX), formaldehyde dehydrogenase (FLD), formate dehydrogenase (FDH) and dihydroxyacetone synthase (DAS). Bar length is proportional to the degree of identity between the *H. polymorpha* protein and the corresponding orthologous protein.

To gain insight into the origin and distribution of MUT pathway genes in different yeast and fungal lineages, we analysed the presence of these encoded proteins in the proteomes of all sequenced ascomycetes yeast and fungi. The obtained pattern (Figure 9) shows a highly uneven distribution of alcohol oxidase and “downstream” metabolic genes in the compared genomes. The presence of MOX orthologs in the genomes of several *Pezizomycotina* species and in the genomes of *Y. lypolitica* and *Zigosaccharomyces rouxii* is not surprising, and is supported by biochemical data proving the capacity of short-chain alcohol oxidases from various *Aspergillus* and *Penicillium* species to use methanol as substrate [93-96] and documented activity of long-chain alcohol-oxidases in *Y. lypolytica* and *Z. rouxii*.

Less homologous to alcohol oxidases encoded by *H. polymorpha* and *P. pastoris* are members of the same glucose-methanol-choline-oxidase (GMC oxidoreductase, Pfam: PF00732) superfamily found in several *Pezizomycotina* genomes (Figure 9). While the presence of AOX genes is always accompanied by the presence of “downstream” genes, these genes, responsible for FA assimilation and oxidation and genes for peroxisomal antioxidative enzymes (catalase, peroxiredoxins) are also found in “AOX-minus” species. This may be explained by the established role of the FA dissimilation branch in the metabolism of methylated nitrogen compounds, detoxification of formaldehyde and other short-chain aldehydes and alcohols. FA assimilation enzymes (DAK and downstream enzymes) also function in the glycerol assimilation and xylose-5-phosphate pathways, and peroxisomes are important for numerous oxidative processes.

Functional expression of endogenous *S. cerevisiae* genes for FA dissimilation or assimilation is supported by biochemical evidence [97], and overexpression of endogenous or exogenous FDH and FLD genes in *S. cerevisiae* can be used to create yeast strains capable of formaldehyde or DHA utilization [98,99] or to develop novel dominant selection markers [100].

Parasitic yeast and fungal species are completely devoid of MUT pathway genes, as are members of the *Saccharomyces sensus stricto* clade, isolated from carbohydrate-rich niches.

To obtain a broader evolutionary retrospective of MUT pathway genes we constructed and compared phylogenetic trees for analysed MUT pathway proteins present in complete *Ascomycetes* genomes. The topology of the trees constructed for “MOX-proteins” and corresponding “MOX genomes” was similar, indicating that MOX-gene evolution in general parallels the evolution of their corresponding genomes without detectable horizontal gene transfer events (Figure S29). Similar results were obtained in the course of phylogenetic analysis of FDH, FLD, DAS and DAK proteins, encoded by yeast and fungal genomes. The topology of the obtained trees was similar for different proteins, and showed clear separation of the *Pezizomycotina* and *Saccharomycotina* branches with no evidence of lateral gene transfer events (Figures S30-32).

## Conclusions

A combination of whole-genome and cDNA pyrosequencing with gap closure enabled us to create a high quality near complete genome sequence of *H. polymorpha* strain DL-1 and to determine the transcription patterns of this strain grown in either methanol or glucose. Transcriptome analyses performed with RNA-seq technology revealed abundant gene expression in methanol and a high level of up-regulation of about 40% of the genes. A notable feature of our analysis as compared to similar studies in other methylotrophic yeast species is a significantly higher level of up-regulation of key methanol utilization, peroxisome biogenesis and antioxidant defence genes compared to microarray data. Phylogenetic analysis revealed that *H. polymorpha*, together with *D. bruxellensis* and *P. pastoris*, is a member of a separate clade of *Saccharomycotina* distinct from the CTG and WGD clades. Comparative analysis of these three yeast species enabled us to identify several shared and unique features of this yeast group related to clade- and species-specific genomic characteristics.

With a compact 9 Mbp genome containing 5325 genes, *H. polymorpha* shows a low level of genome redundancy and duplications, similar to that of *P. pastoris*, indicating that it did not experience an ancestral whole genome duplication. Intergenome comparisons revealed extensive reshuffling of gene order between the three yeasts and a higher level of syntheny was observed between *H. polymorpha* and the non-methylotrophic yeast species *D. bruxellensis*. Closer examination of gene order conservation in the extended *H. polymorpha* chromosomal regions spanning the *H. polymorpha* AOX gene and orthologous *D. bruxellensis* chromosomal loci enabled us to identify a gene loss event including AOX gene deletion that likely occurred during the evolution of *D. bruxllensis* from an apparently methylotrophic common ancestor of *H. polymorpha* and *D. bruxllensis*. Comparative phylogenetic analysis showed that MUT pathway genes are conserved in several *Pezizomycotina* lineages, indicating their potential capability to use methanol as a carbon and energy source.

The availability of genomic sequences of DL-1 and other *H. polymorpha* strains opens many new opportunities to improve our understanding of many still insufficiently characterized aspects of *H. polymorpha* life cycle, physiology and metabolism, including mechanisms of methanol sensing, regulation of methanol-induced gene expression, peroxisome biogenesis, and autophagy. Further application of whole-genome analytic techniques may help to identify new important *cis* elements regulating gene expression, chromosome replication and segregation, - constitutive and regulated promoters, chromosomal replication origins and centromeres. Combined with recently developed new tools for genetic manipulation in *H. polymorpha*[101], such intrinsic *H. polymorpha* traits as thermotolerance and more tunable control of methanol induced gene expression as compared to *P. pastoris*, this knowledge may lead to further improvements of *H. polymorpha* as a “microbial cell factory”, especially in the field of metabolic engineering towards high-temperature ethanol production and the creation of new hosts for the production of complex and multisubunit proteins, including the challenging task of developing glycoengineered *H. polymorpha* strains [9] capable of producing humanized glycoproteins, similar to what was achieved for *P. pastoris*.

## Methods

### H. polymorpha strain and DNA isolation

The *H. polymorpha* strain DL-1 (ATCC26012) was kindly provided by Prof. Michael Ter-Avanesyan from the N. Bach Institute of Biochemistry RAS. Genomic DNA was isolated from 1.5 ml of fresh overnight culture. Cells were collected by centrifugation and resuspended in 0.3 ml lysis buffer (1% SDS, 0.1 M NaCl, 0.01 M Tris–HCl, 0.001 M EDTA, 2% Triton X-100), and glass beads (Sigma #G-8772) were added. The mixture was shaken for 4 min. Total DNA was purified by chloroform extraction, and finally precipitated with isopropanol and dissolved in 0.05 ml of water for further use.

### Genome sequencing and assembly

The genome was sequenced using a pyrosequencing approach on a GS FLX genome sequencer (Roche, Switzerland). A shotgun genome library was generated using *H. polymorpha* DL-1 genomic DNA and the GS FLX Titanium Rapid Library Preparation Kit (Roche) according to the protocol provided by the manufacturer. Second, an 8-kbp Paired End library was generated according to the GS FLX Paired-end Library Preparation Kit (Roche). The DNA libraries were amplified by emulsion PCR and sequenced applying the Titanium sequencing chemistry and PicoTiterPlate (454 Life Sciences, Roche). The GS FLX reads were *de novo* assembled into contigs and then ordered into scaffolds using Newbler Assembler 2.0 (454 Life Sciences, Branford, CT).

### Transcriptome analysis

*H. polymorpha* DL-1 was grown up to OD_660_ ~2.0 in 0.67% YNB medium containing leucine (20 mg/l) and either 1% glucose or 1% methanol at 37°C while shaking at 250 rpm. Cells were harvested by centrifugation (4000 rpm, 10 min, 4°C) and taken up in AE-buffer (50 mM sodium acetate, 10 mM EDTA, pH 5.0). The total RNA was extracted by a hot phenol method followed by purification using RNeasy Mini Kit (Qiagen).

Two total RNA samples were used for cDNA synthesis employing the SMART approach [102]. Synthesis and amplification of cDNA was performed by Evrogen Ltd (Moscow, Russia). cDNA samples were sequenced using a pyrosequencing approach on a Roche GS FLX genome sequencer according to the standard protocol for a shotgun genome library. GS FLX reads were mapped to the genome using GS Reference Mapper 2.8 and the number of reads mapping to each gene was calculated with BEDTools 2.12.0. The expression level of each particular gene was normalized by library size: the normalized expression level of each particular gene was calculated as the number of reads mapped to this gene divided by the total number of reads mapped to the whole genome. The RNA seq data obtained for glucose and methanol-grown cells are available in the SRA database - Acc## SRX365635 and SRX365636 respectively.

### Genome annotation and analysis

Prediction of coding sequences was done by applying AUGUSTUS software version v2.7 [103,104] using training set and hints obtained from transcriptome assembly. tRNA genes were predicted with tRNAscan-SE [105] and rRNA genes with RNAmmer [106]. The transcriptome was assembled by GS *De Novo* Assembler 2.8 (454 Life Sciences, Branford, CT), then open reading frames corresponding to genes were extracted from the assembled transcripts by the EST/cDNA version of GeneMarkS [107].

Redundant genes, transcripts with partially assembled 5' ends or incorrect gene start should be excluded before Augustus training. We used BLATCLUST to make a non-redundant training set [108] and BLAST to find homologs for our genes in the NCBI protein database. Only genes that had the same start as three or more blast homologs were kept, then mapped to the genome by BLAT [109] with default parameters and transformed into intron-exon structures by Scipio [110] and used for optimizing Augustus parameters. The transcriptome assembly was mapped to the *H. polymorpha* DL-1 genome using BLAT and was used as hints for Augustus gene prediction.

Furthermore we mapped reads to the genome by TopHat [111] and assembled them into transcripts by Cufflinks [112]. The second assembly was used for additional hints and for the following curation. Augustus prediction, reading and transcript mapping were visualized in IGV browser [113] for manual curation of problematic cases, when prediction is inconsistent with transcript assemblies.

The integrated RAPYD bioinformatic platform, covering eukaryotic gene prediction, genome annotation and comparative genomics was applied for global and regional functional annotation [114]. The RAPYD functional annotation pipeline was used to assign predicted proteins with InterPro domains, KOG categories and mapping of GO terms. Final annotation was built based on the RAPYD pipeline and manually curated using BLASTP search against NCBI protein database.

In order to validate the completeness of the obtained sequence we checked it for the presence of a set of 248 core eukaryotic genes identified by comparative analysis of 6 model organisms [115]. All these genes were shown to be present with full domain coverage.

Repetitive DNA sequences, including interspersed and simple repeats and low complexity regions were identified with Repeatmasker [116] using default settings for yeast genomes.

BLAST2GO [117] was also used for mapping of Gene Ontology terms, INTERPRO domains and subsequent GO enrichment analysis of subtelomeric genes and genes specifically overexpressed and up-regulated in glucose-grown and methanol-grown cells.

### Phylogenetic analysis

Phylogenetic analysis was performed for a concatenated alignment of 153 universally distributed orthologs previously identified in 42 sequenced fungal genomes (Additional file 2: Table S16). A multiple sequence alignment was constructed using the MUSCLE program contained within the MEGA5 package [118] and poorly aligned positions and gap positions were removed with gblocks [119]. We used RAxML v7.3.5 [120] to compute the maximum likelihood phylogenetic tree with a gamma model of rate heterogeneity (4 discrete rate categories, an estimated alpha-parameter) and JTT substitution matrix. We conducted 100 bootstrap replicates to define the support values on the tree. Phylogenetic tree is available from TreeBASE (TB2:S14826).

A phylogenetic analysis of methanol-utilization pathway genes was performed using NCBI databases and tools. Briefly, orthologs of *H. polymorpha* alcohol oxidase (AOX), formaldehyde dehydrogenase (FLD), formate dehydrogenase (FDH), digydroxyacetone kinase (DAK) and dihydroxyacetone synthase (DAS) were identified by BLAST search against the NCBI fungal genomes database. Orthologs were aligned with online COBAL tools and used to generate Newick trees using fast minimum evolution algorithms. Trees were visualized and formatted using MEGA5 tree viewer. Phylogenetic analysis of *H. polymorpha* MFS transporters was performed with Ugene tools [121].

### Genome redundancy estimation and comparative genomic analysis

Identification of shared and specific protein sets for three compared genomes (*H. polymorpha, P. pastoris, D. bruxellensis)* was performed using the EDGAR tool [91]. Whole-genome alignments between *H. polymorpha* genome and *P. pastoris* chromosomes were performed using the Promer program of the MUMmer package [122]. For pair-wise comparisons between the *H. polymorpha* and *D. bruxellensis* genomes, *D. bruxellensis* contigs larger than 100 kb were used.

For estimation of the degree of synteny conservation between compared genomes we made a dot-plot using blast and custom perl scripts, that visualizes pairs of protein homologs that are symmetrical best hits between two genomes. Synteny maps for selected *H. polymorpha* loci spanning methanol-utilization genes were created with in-house scripts. Custom scripts were also used to create *P. pastoris, D. bruxellensis* and *H. polymoprha* codon frequency tables.

To evaluate genome redundancy at the DNA level we used the same approach described for analysis of *D. bruxellensis* duplicated sequences [84]. The *H. polymorpha* genome was split into non-overlapping 2000 bp or 5000 bp fragments that were used for local BLAST search (e-value 1e^-10^, at least 1/3 of the fragment aligned) against the whole *H. polymorpha* genome regions spanning 2000 or 5000 nucleotides. Only sequences with 2 or 3 hits and similarity levels higher than 70%, 80%, and 90% were recorded. A similar analysis was performed for the *P. pastoris, D. bruxellensis and S. cerevisiae* genomes.

The extent of genome redundancy at the protein level was estimated as the ratio of the total number of predicted CDS to the number of protein families. The latter were identified by subjecting the predicted *H. polymorpha* proteome to OrthoMCL clustering [123]. Protein families defined after mapping the proteome to OrthoMCL-DB were used to calculate the number of protein families with one, two, three, or more paralogous genes per family.

### Availability of supporting data

All the supporting data are included as additional files.

## Abbreviations

ARS: Autonomously replicating sequence; ATG genes: Autophagy-related genes; CTG group: Yeast clade with genetic code alteration (CTG coding for serine instead of leucine); LTR: Long terminal repeat; RAPYD: Rapid annotation platform for yeast data; MFS: Major facilitator superfamily; WGD group: Yeast clade with ancestral whole genome duplication.

## Competing interests

The authors declare that they have no competing interests.

## Authors’ contributions

NR conceived of the research and designed the study, ME wrote the paper, and EM and MZ helped with the manuscript preparation. EM cultured the cells, isolated DNA and RNA and analysed transcriptome data, OD, ES and MZ analysed telomeres and subtelomeric sequences, AM and VK performed DNA sequencing and assembly, AB and JS performed sequence annotation and bioinformatics analysis, KS was general supervisor and involved in writing the paper. All authors read and approved the final manuscript.

## Supplementary Material

Additional file 1 Supplementary figures. Click here for file

Additional file 2 Supplementary tables. Click here for file

Additional file 3 Validation of RNA-seq data by quantitative PCR. Click here for file

Additional file 4 Confirmation of alternative splicing events. Click here for file
