# Supplementary material for: Genome sequence and analysis of methylotrophic yeast Hansenula polymorpha DL1
Source: BMC Genomics. 2013 Nov 27;14:837. doi: 10.1186/1471-2164-14-837 (PMC3866509; doi:10.1186/1471-2164-14-837)
Supplement: Additional file 1 — Supplementary figures. [file 1471-2164-14-837-S1.pdf]

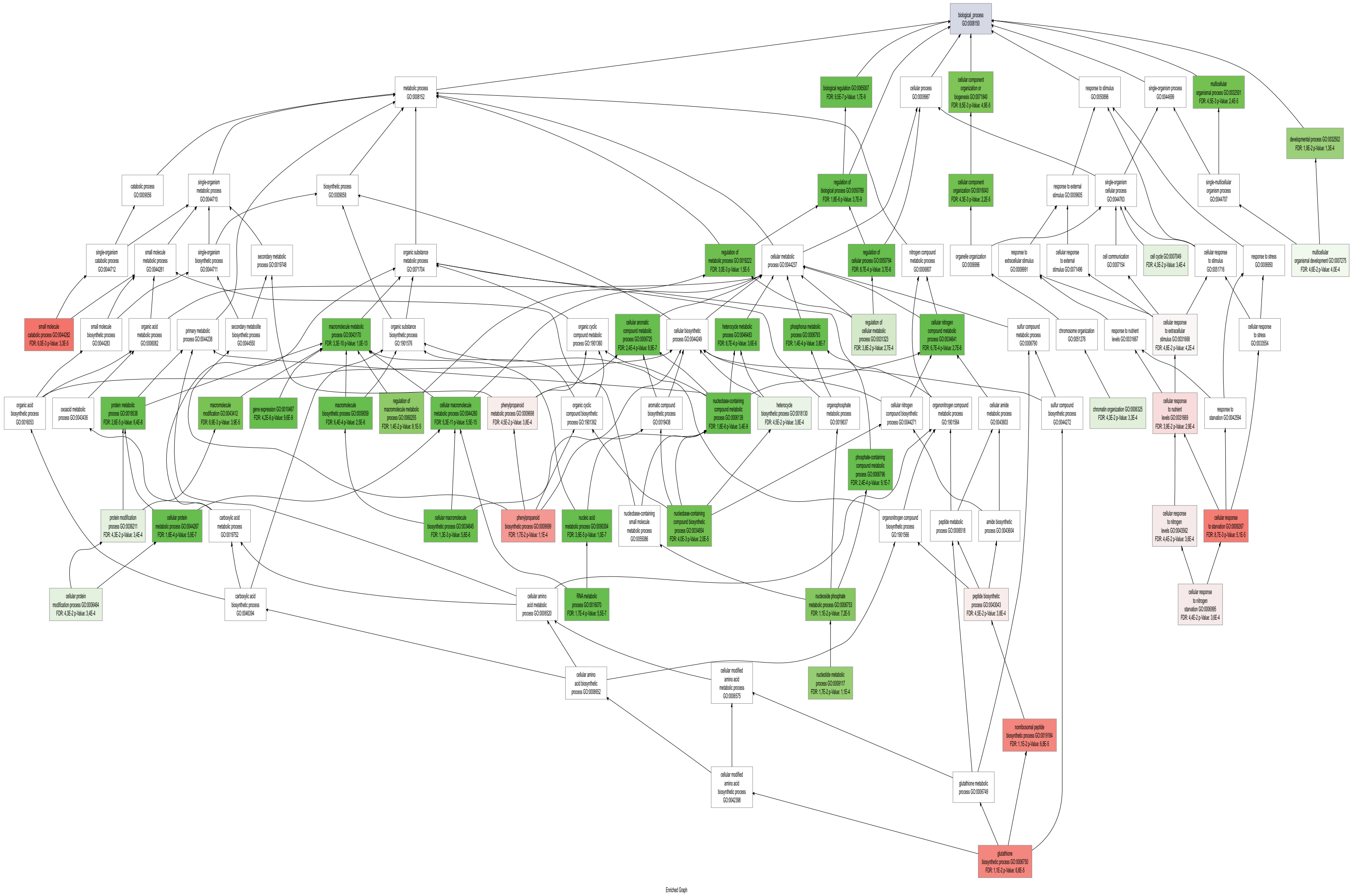

Figure S1. GO enrichment analysis of subtelomeric genes. Biological process.
Directed Acyclic Graph of the enriched GO categories. The 280 -member set of subtelomeric genes (20 genes at the telomere proximal regions of the each of seven chromosomes) was put to gene ontology analysis. The graph shows the significance of overexpressed genes and non-randomly present GO terms. Categories with significantly enriched gene numbers as determined by hypergeometric test are indicated in red, those with depleted - in green.

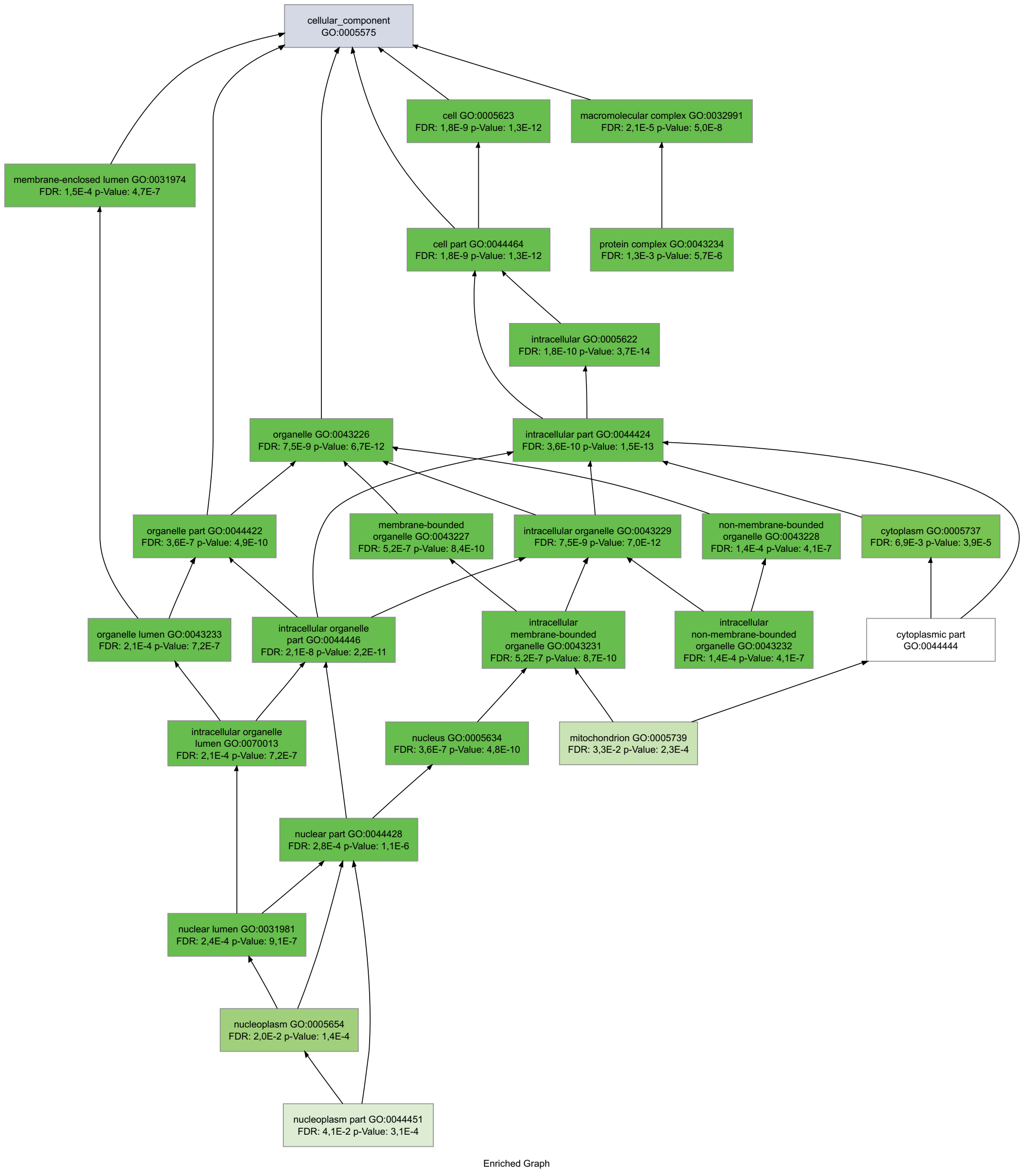

Figure S2. GO enrichment analysis of subtelomeric genes.Cell component.
Graph explanation as on figure S1.

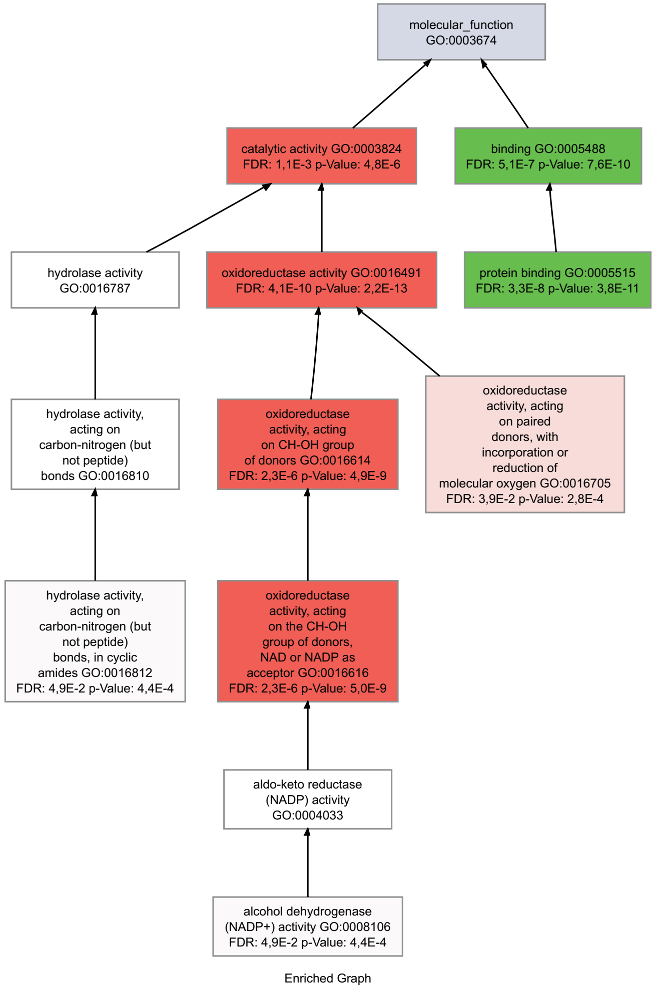

Figure S3. GO enrichment analysis of subtelomeric genes. Molecular function.
Graph explanation as on figure S1.

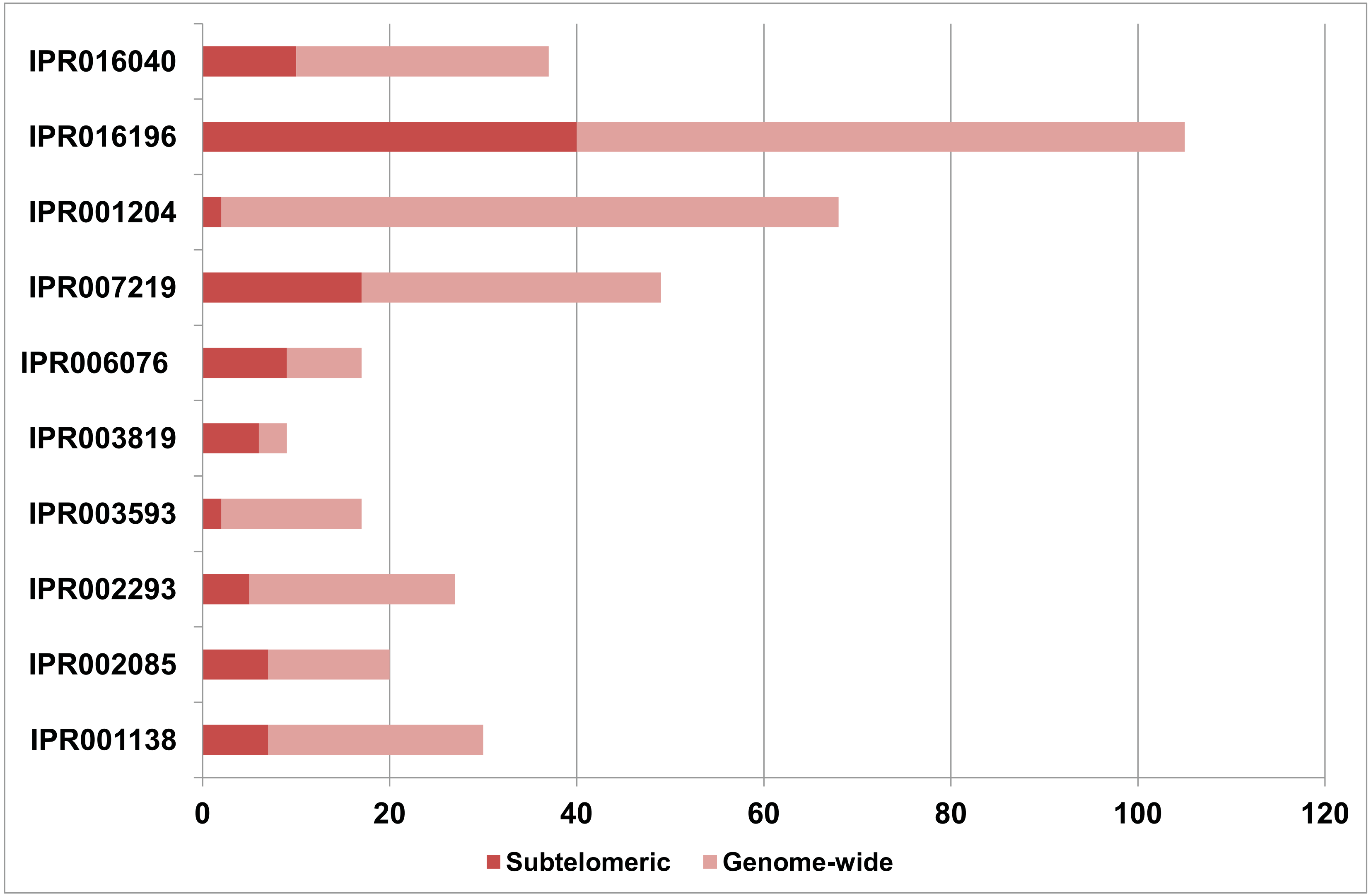

Figure S4. Characteristic subtelomeric and non-subtelomeric genes in *H.polymorpha* genome.
For each protein family the number of subtelomeric (dark purple) and "internal" (light purple) genes is plotted. Classification is based on INTREPRO categories: IPR003819 Taurine catabolism dioxygenase TauD/TfdA, IPR006076 - FAD dependent oxidoreductase, IPR007219 - Transcription factor, fungi, IPR003692- Hydantoinase B/oxoprolinase, IPR016196 - Major facilitator superfamily domain, general substrate transporter, IPR016040 - NAD(P)-binding domain, IPR001138- Zn(2)-C6 fungal-type DNA-binding domain, IPR002085 - Alcohol dehydrogenase superfamily, zinc-type, IPR002293 - Amino acid/polyamine transporter I, IPR003593 - proteins with AAA+ ATPase domain.

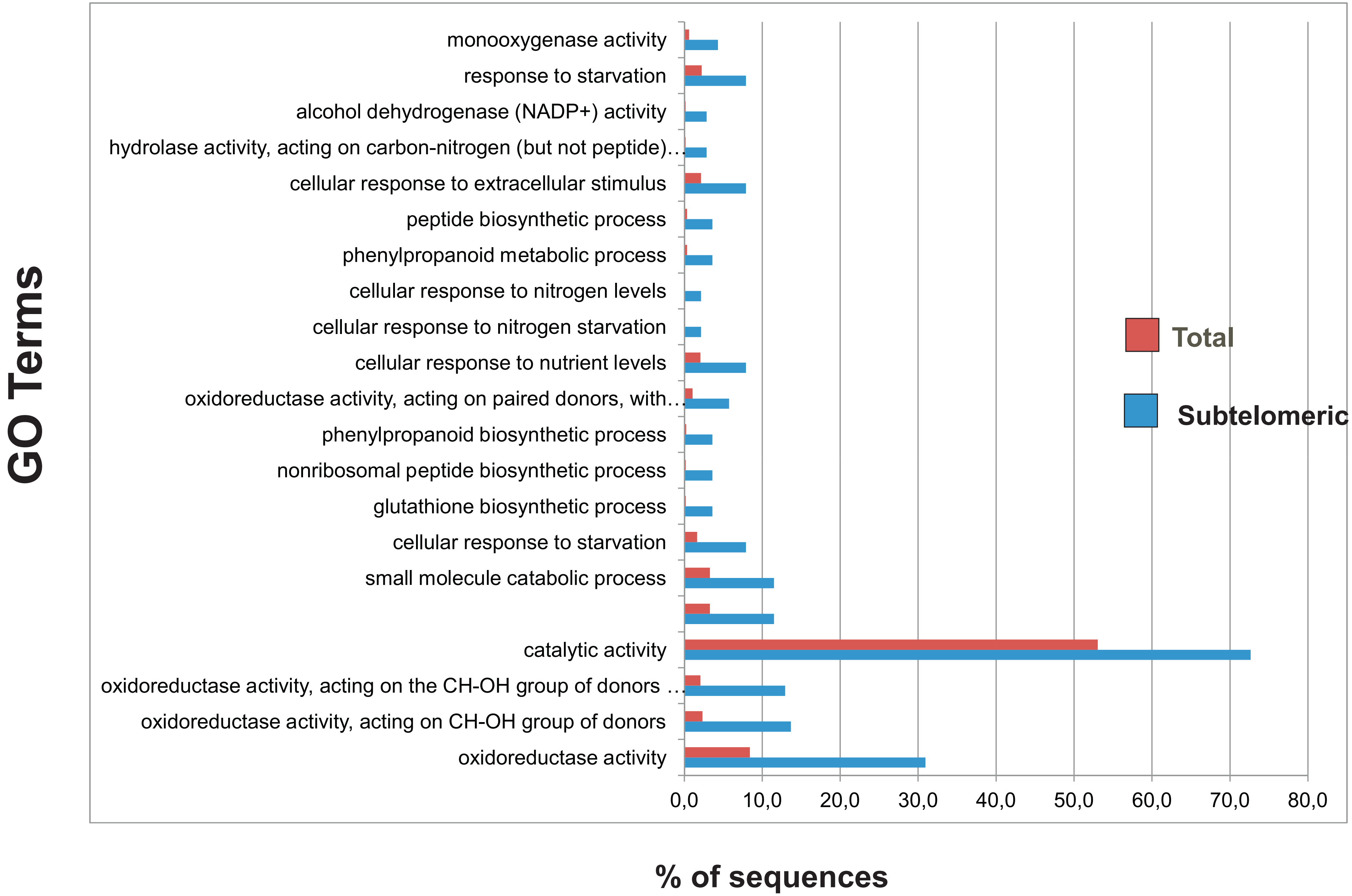

Figure S5. Differential GO term distribution. Subtelomeric genes.
Bar diagram of functional category enrichment analysis (Fisher's Exact Test) of *H.polymorpha* subtelomeric genes (Blue) and total set of H.polymorpha genes (Red). The Y-axis is the percentage of genes mapped by the term, and represents the abundance of the GO term. The percentage for the subtelomeric genes is calculated by the number of genes mapped to the GO term divided by the number of all genes in the test set. The same calculation was applied to the reference list to generate its percentage. The X-axis is the definition of GO terms.

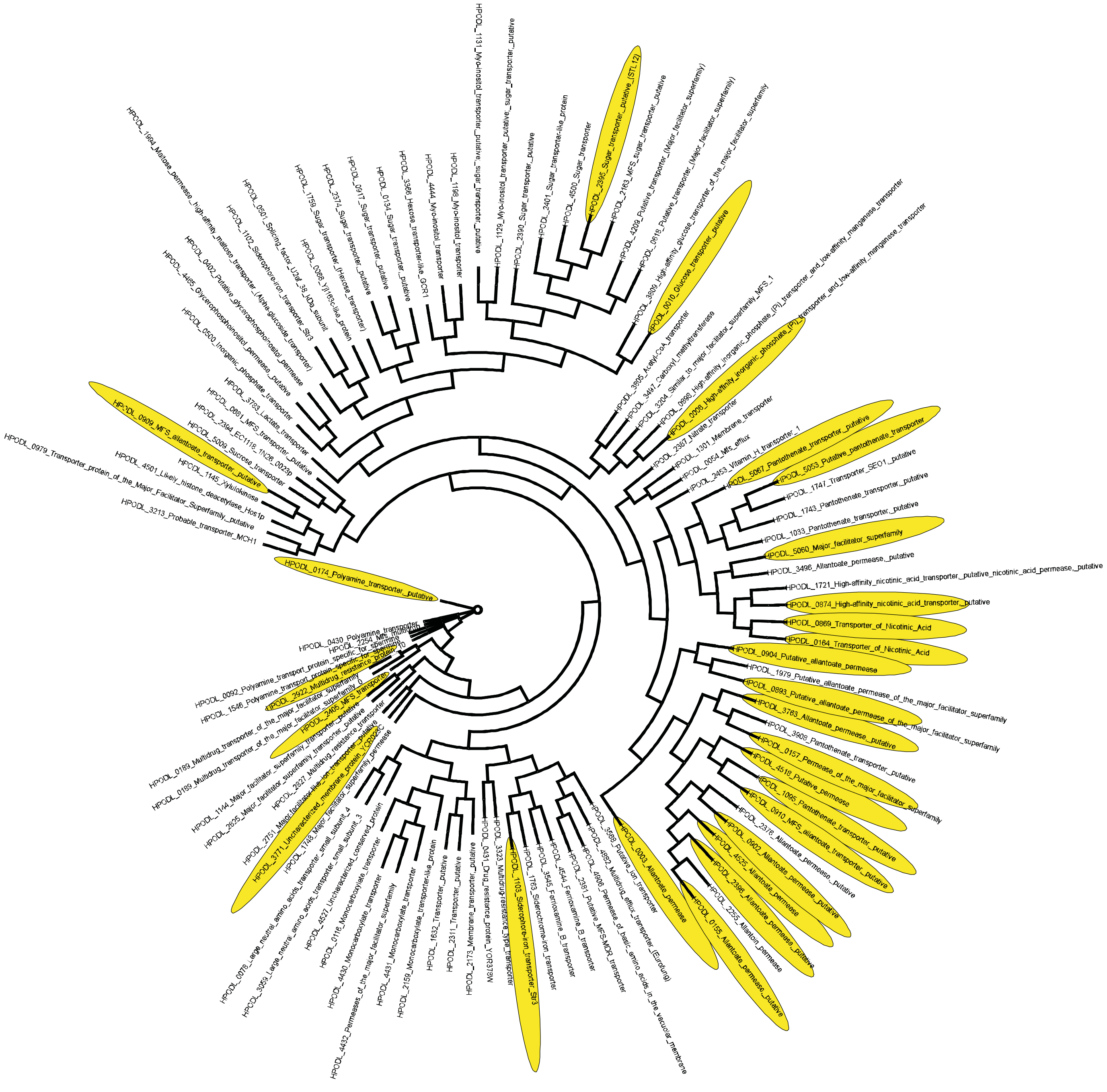

Figure S6. Phylogenetic tree of MFS transporters encoded by *H.polymorpha* genome. Colored in yellow are genes located in subtelomeric regions.

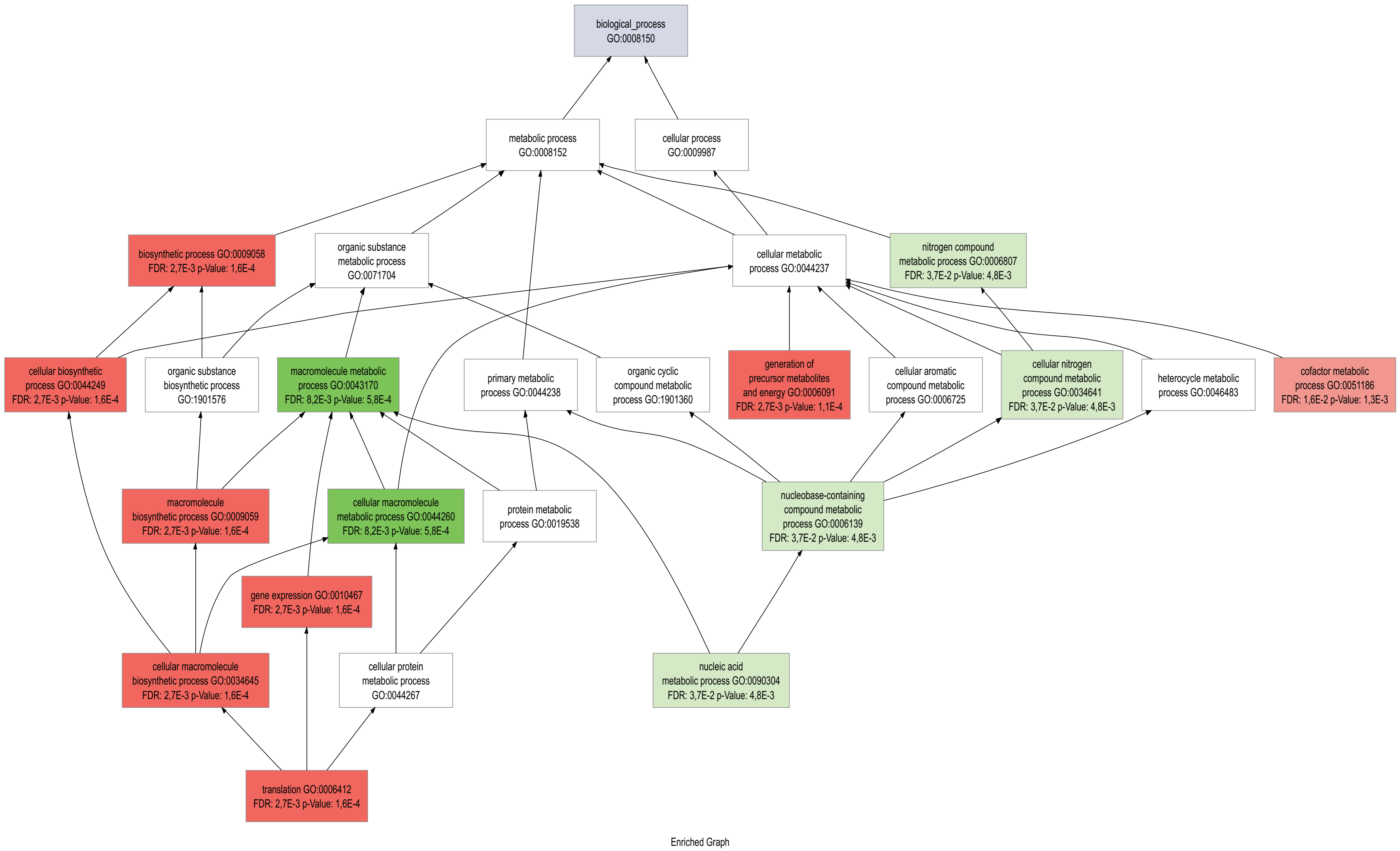

Figure S7. Genes, overexpressed on glucose. Biological process
Directed Acyclic Graph of the enriched GO categories. The 600-member set of genes over expressed on glucose (log2tag count >8) was put to the gene ontology analysis. The graph shows the significance of overexpressed genes and non-randomly present GO terms. Categories with significantly enriched gene numbers as determined by hypergeometric test are indicated in red

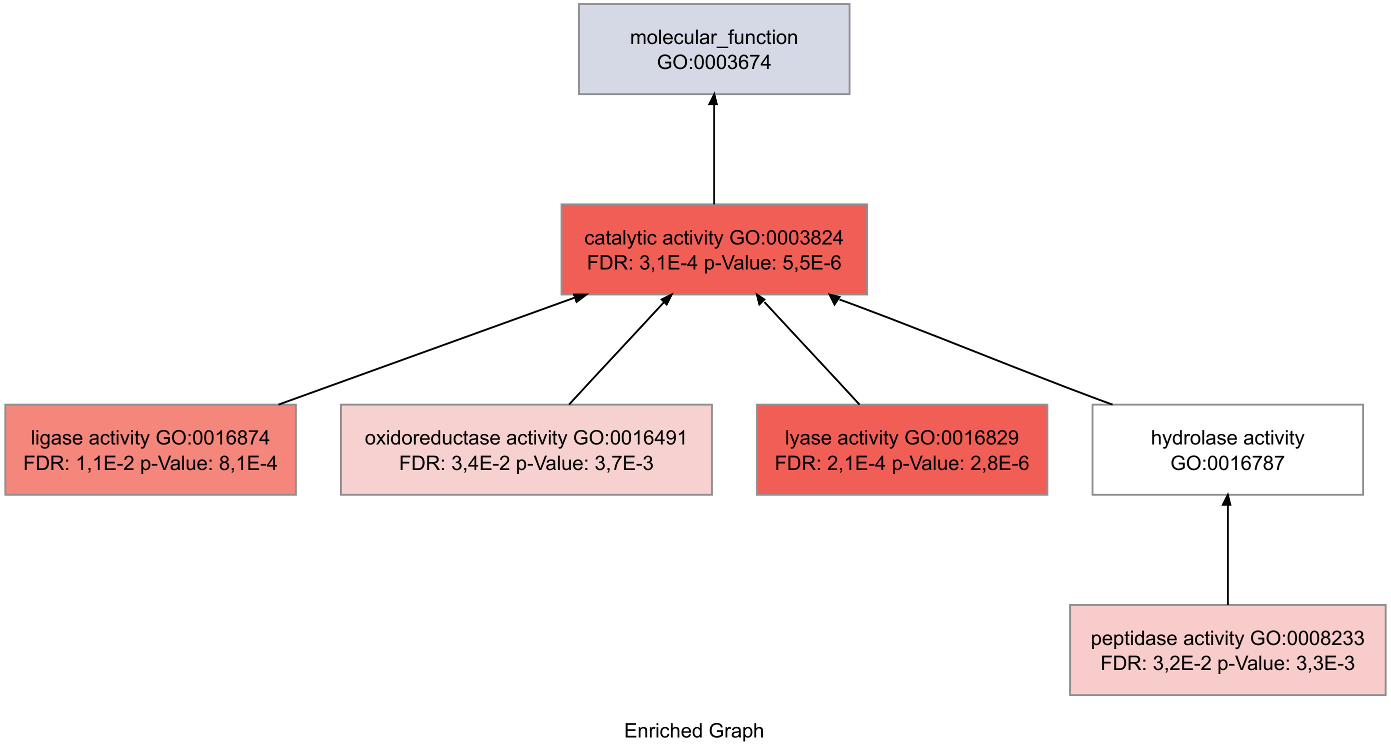

Figure S8. Genes, overexpressed on glucose. Molecular function.
Graph explanation - same as on Figure S7.

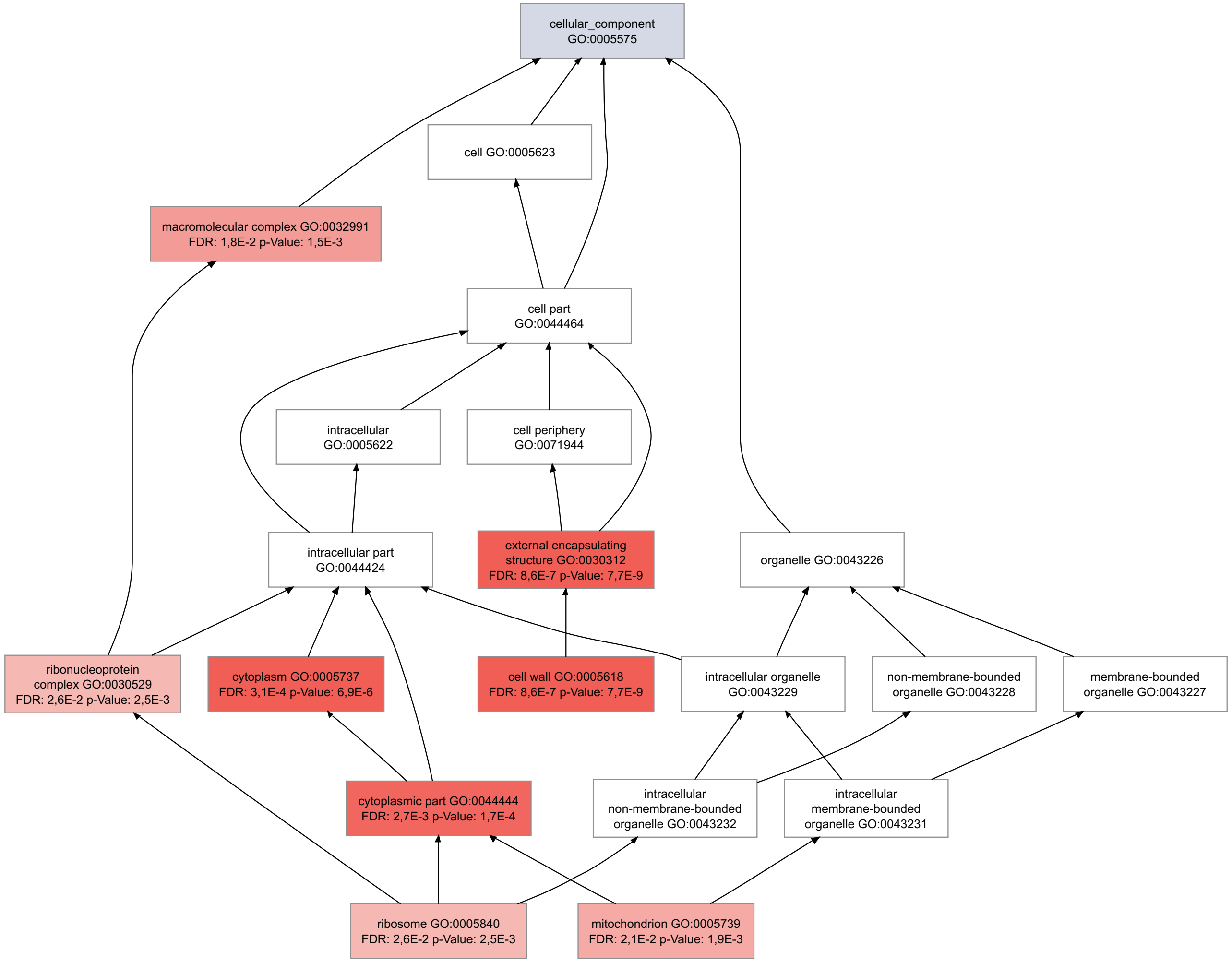

Enriched Graph

Figure S9. Genes, overexpressed on glucose. Cell component.
Graph explanation - as on Figure S7.

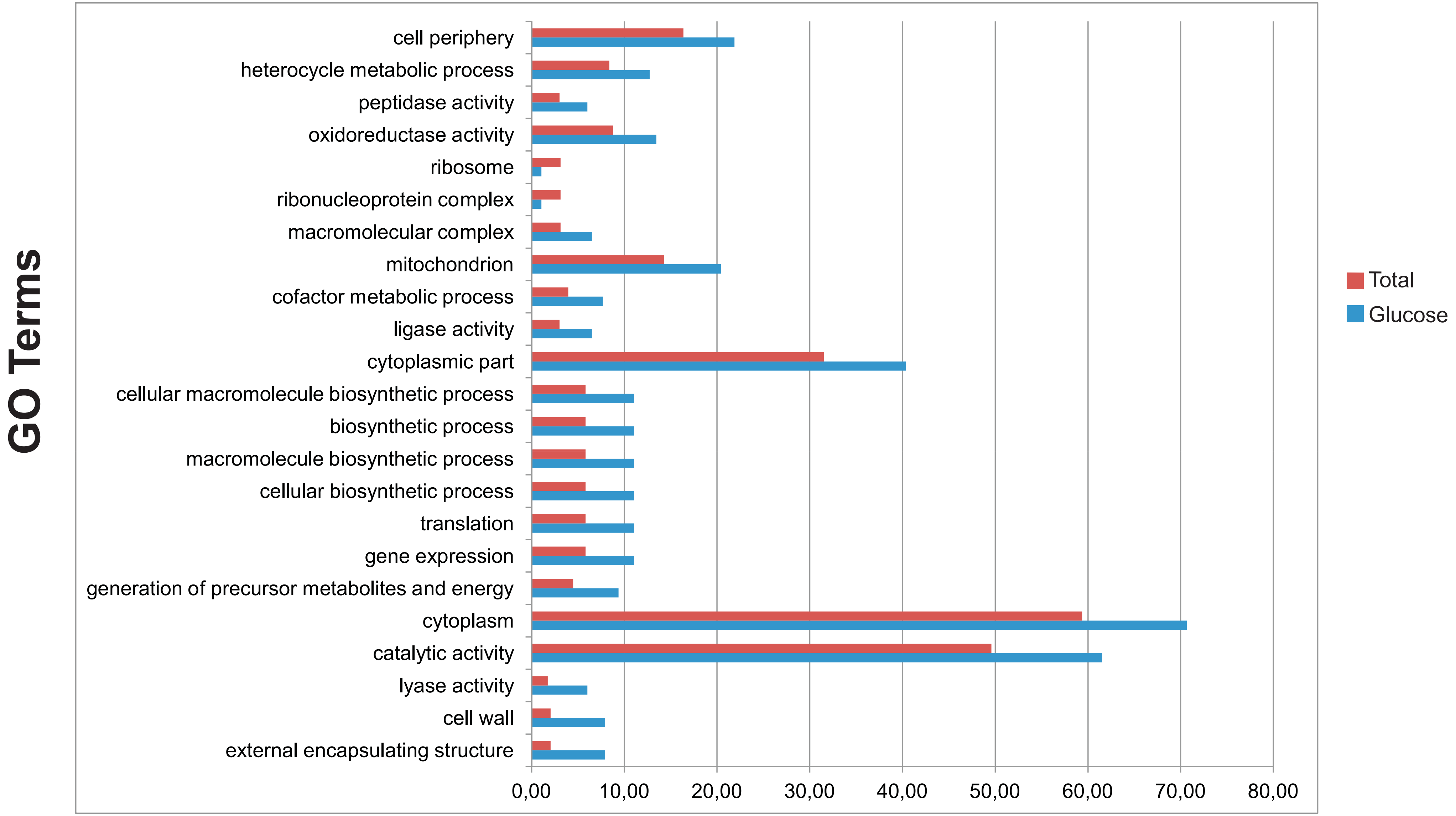

Figure S10. Differential GO term distribution. Genes overexpressed on glucose
Bar diagram of functional category enrichment analysis (Fisher's Exact Test) of H.polymorpha glucose overexpressed genes (Test Set) and total set of H.polymorpha genes (Reference Set). The Y-axis is the percentage of genes mapped by the term, and represents the abundance of the GO term. The percentage for the (Test Set) is calculated by the number of genes mapped to the GO term divided by the number of all genes in the test set. The same calculation was applied to the reference list to generate its percentage. These two lists are represented using different custom colors. The X-axis is the definition of GO terms..

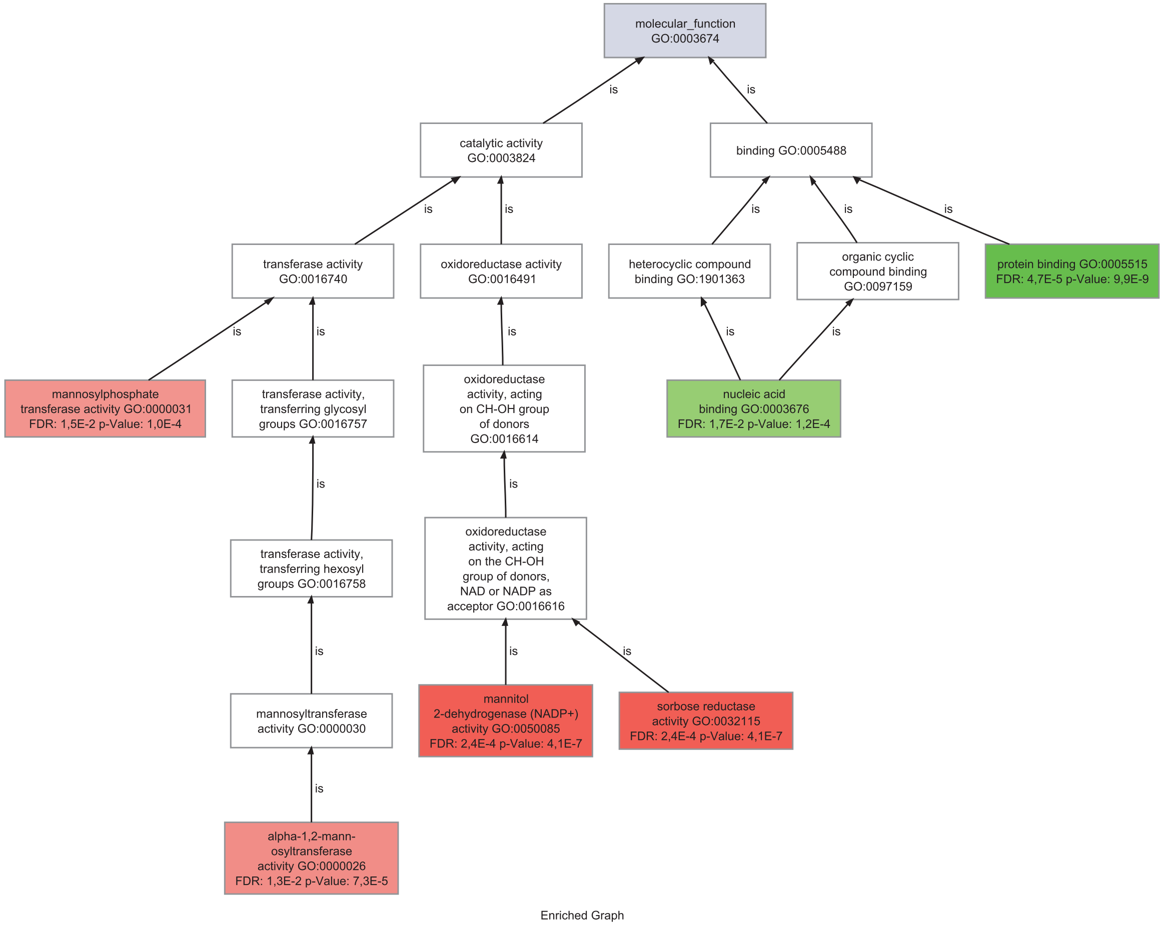

Figure S11. Genes, overexpressed on methanol. Molecular function.
Directed Acyclic Graph of the enriched GO categories. The 500-member set of genes over expressed on methanol (log2tag count >8) was put to the gene ontology analysis. The graph shows the significance of overexpressed genes and non-randomly present GO terms. Categories with significantly enriched gene numbers as determined by hypergeometric test are indicated in red.

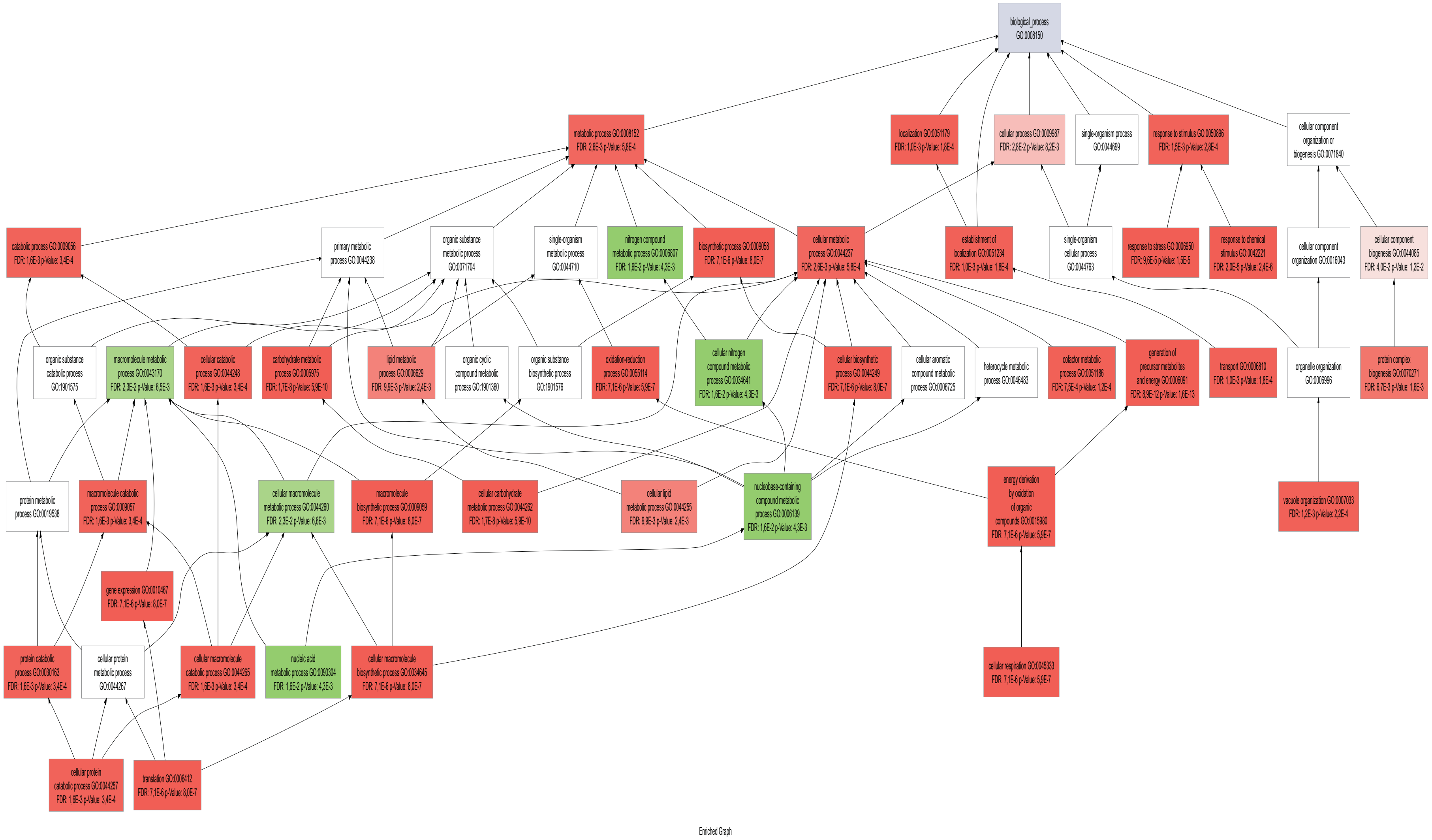

Figure S12. Genes, overexpressed on methanol.Biological process.
Graph explanation - as on Figure S7.

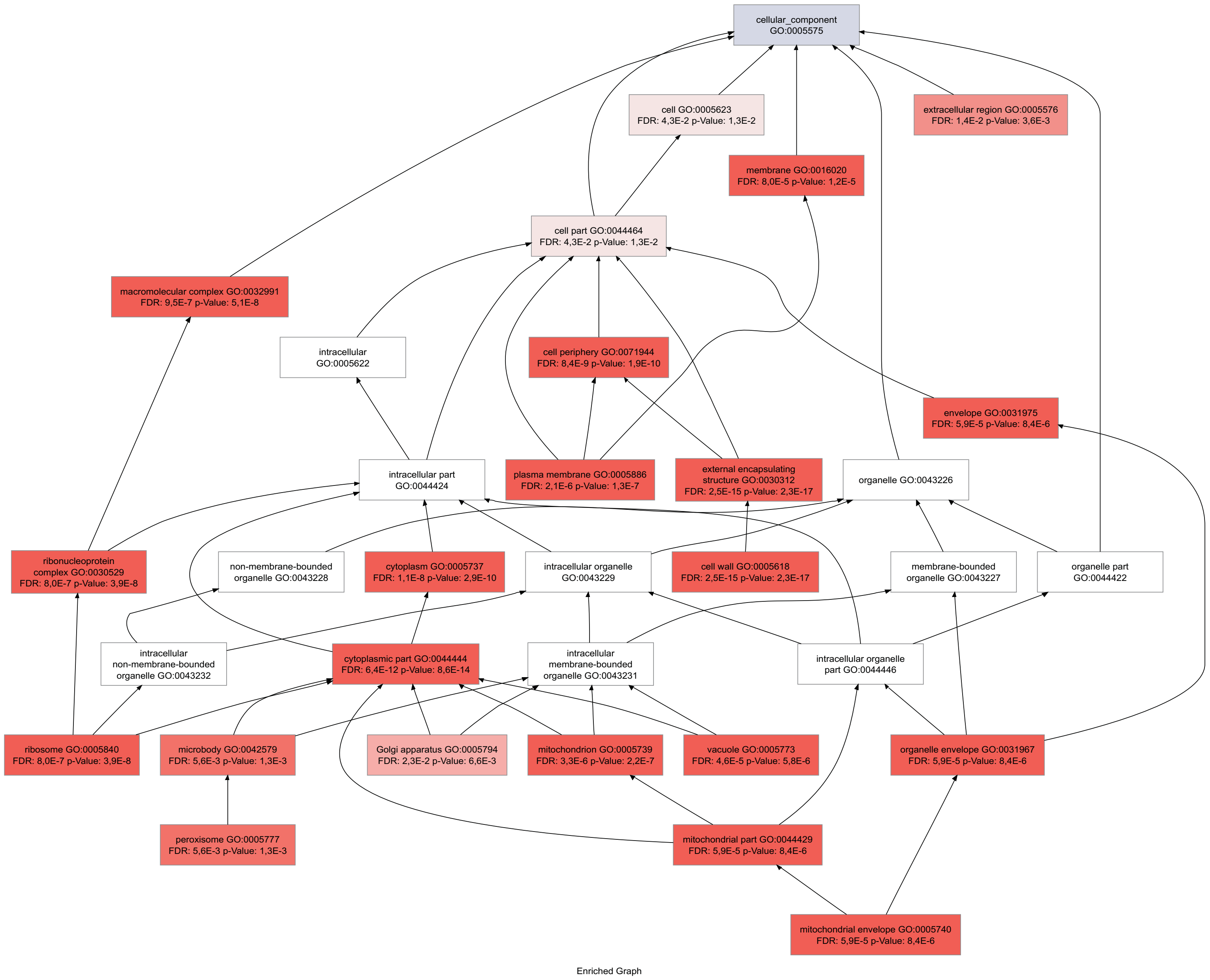

Figure S13. Genes, overexpressed on methanol. Cell component.
Graph explanation - same as on Figure S7.

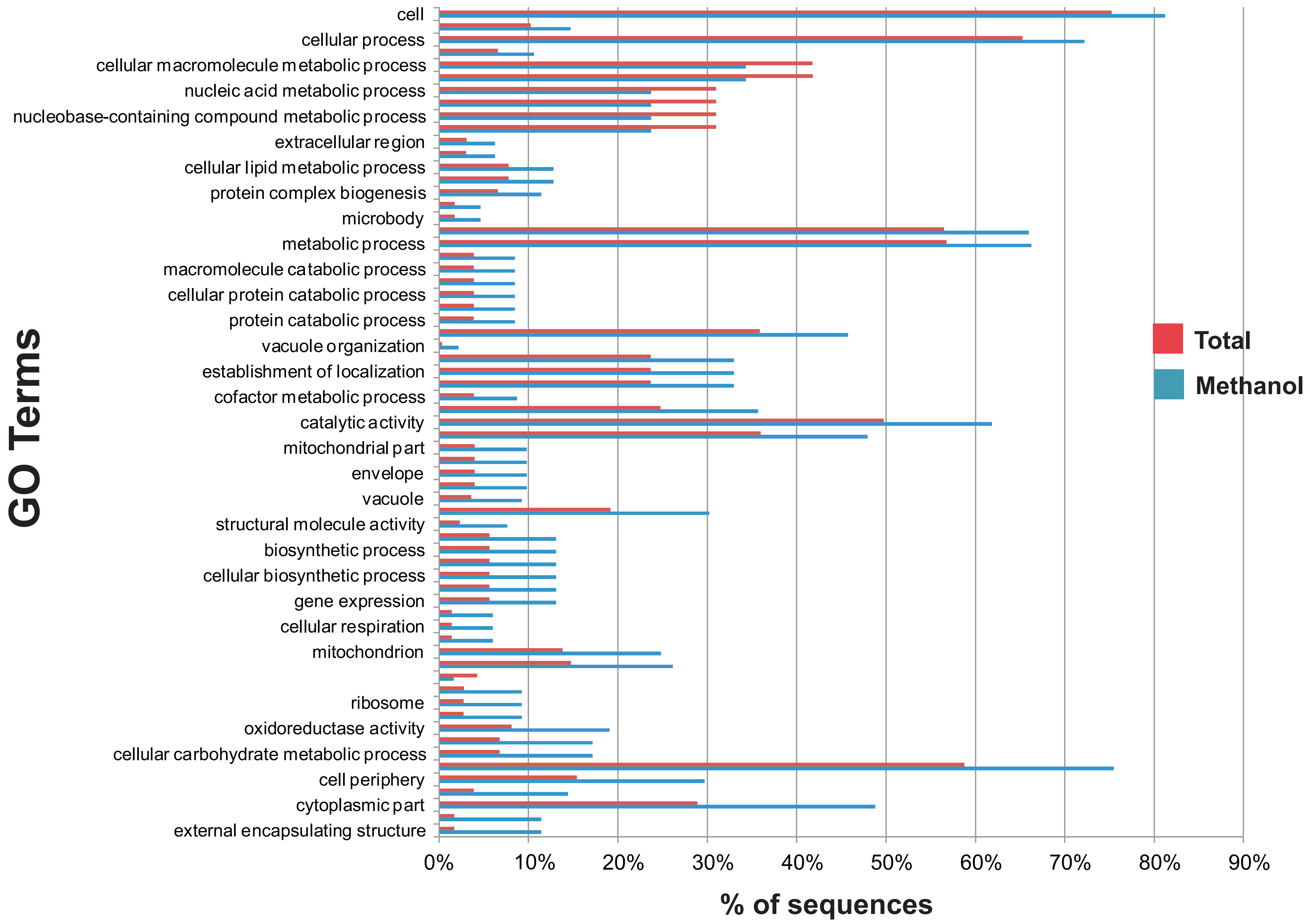

Figure S14. Differential GO term distribution. Genes overexpressed on methanol.
Chart explanation - as on Figure S10.

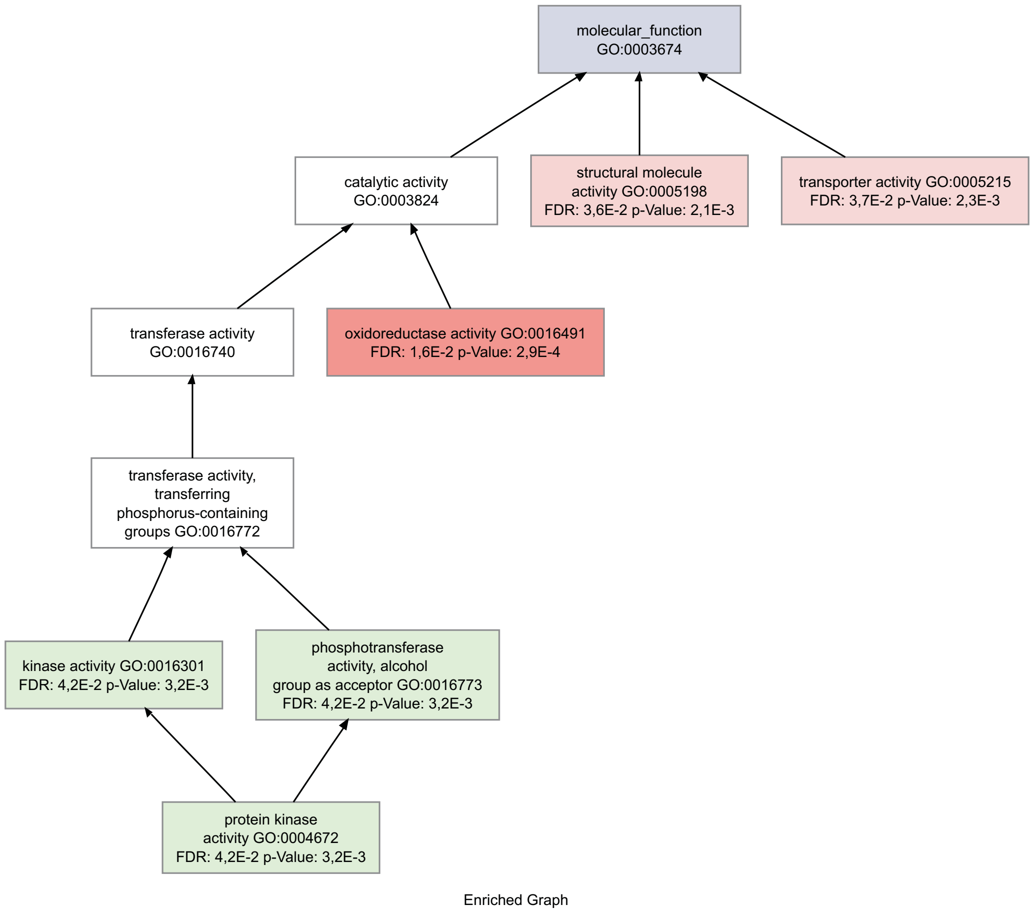

Figure S15.Genes, upregulated on methanol. Molecular function.
Graph explanation - same as on Figure S7.

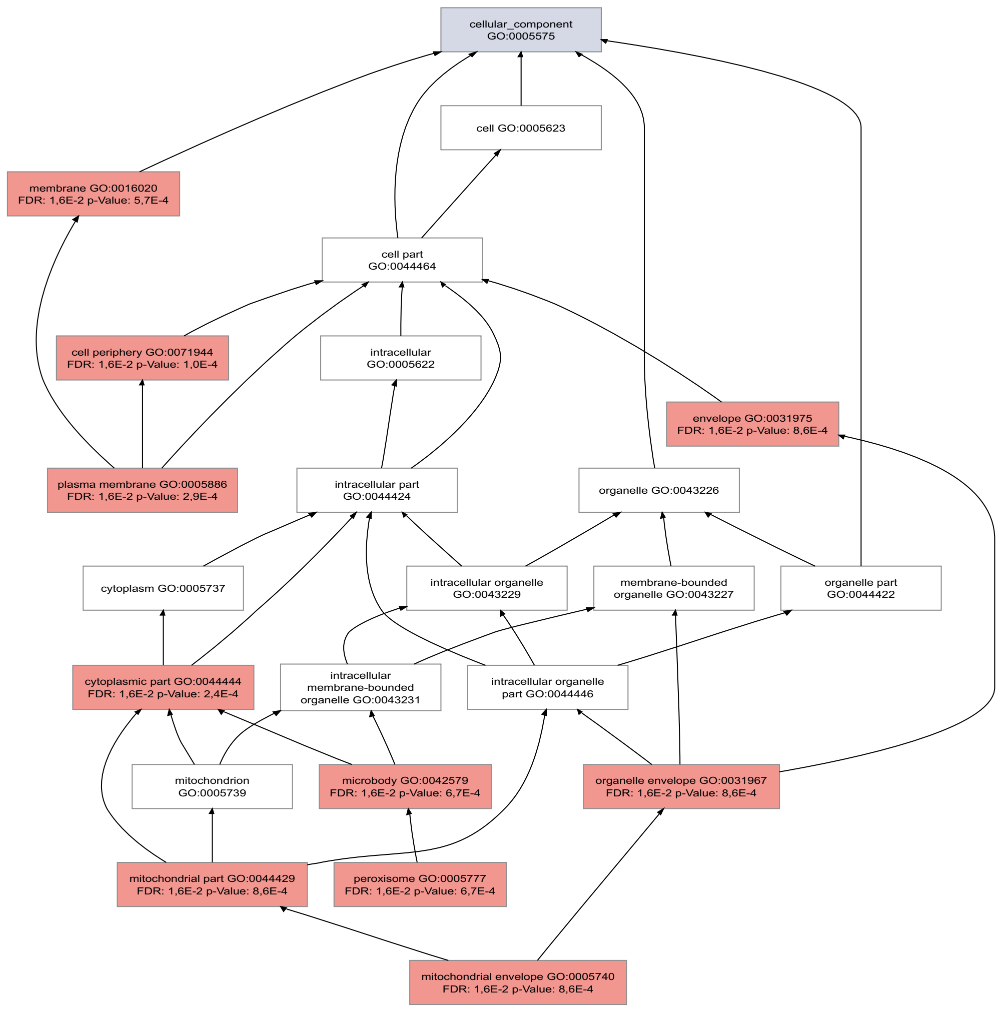

Figure S16. Genes, upregulated on methanol. Cell component.
Graph explanation - same as on Figure S7.

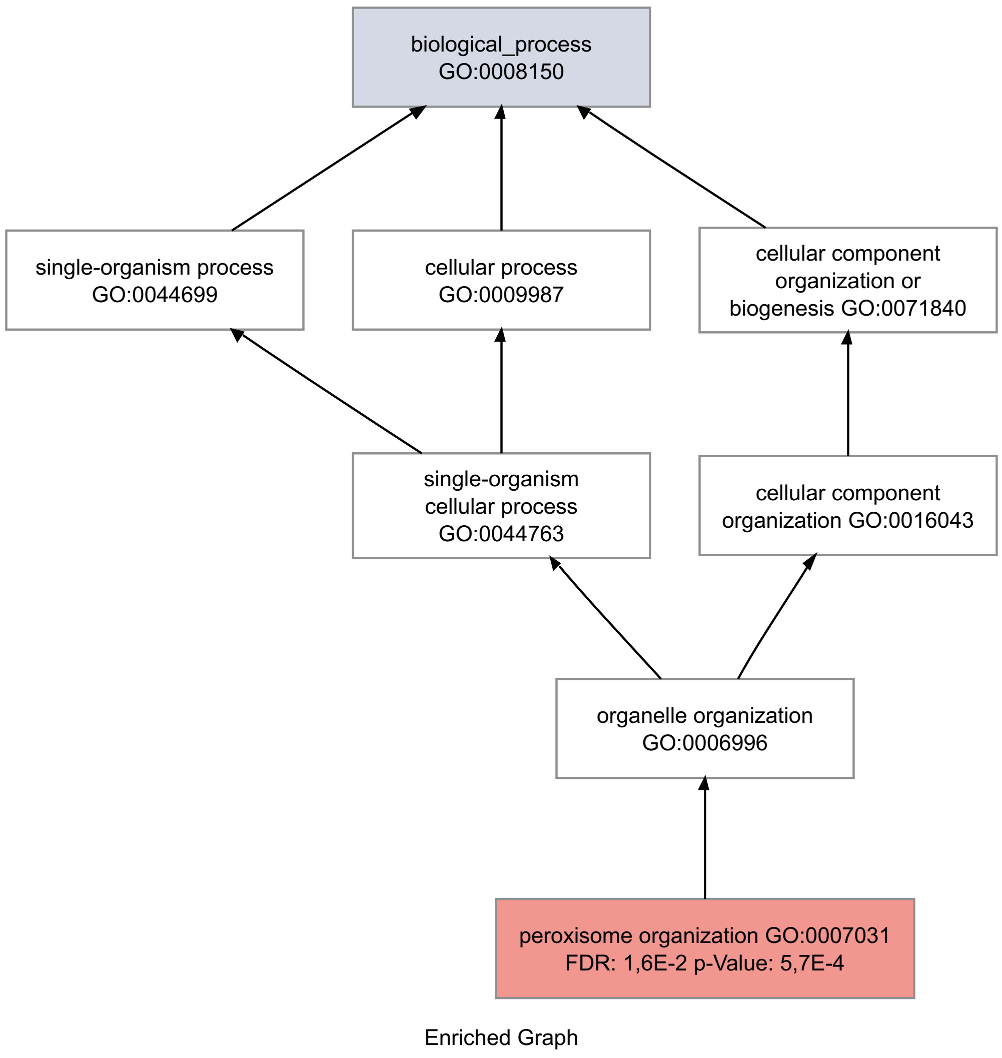

Figure S17. Genes, upregulated on methanol.Biological process.
Graph explanation - as on Figure S7.

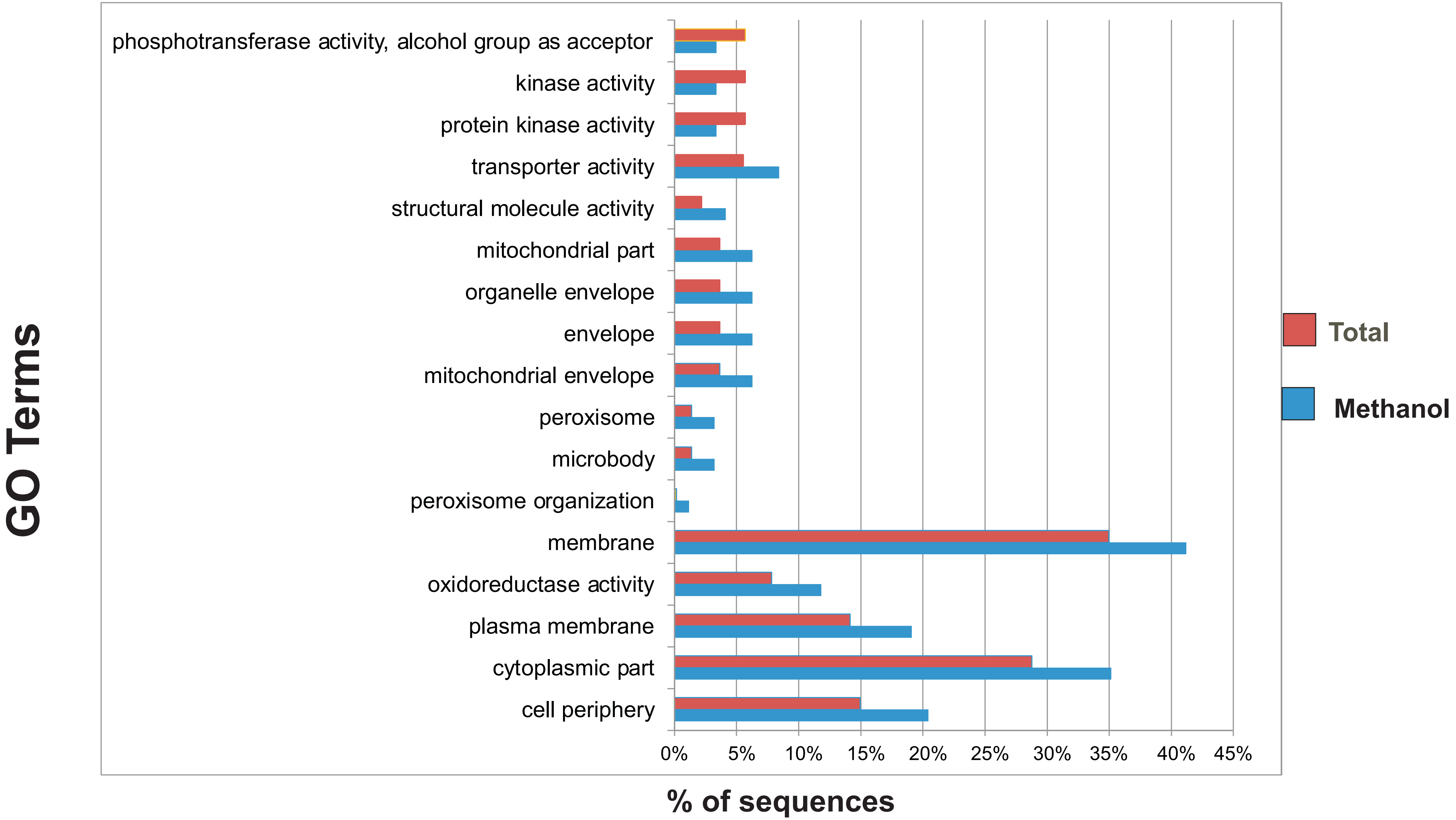

Figure S18 Differential GO term distribution. Genes upregulated on methanol
Chart explanation - same as on Figure S10.

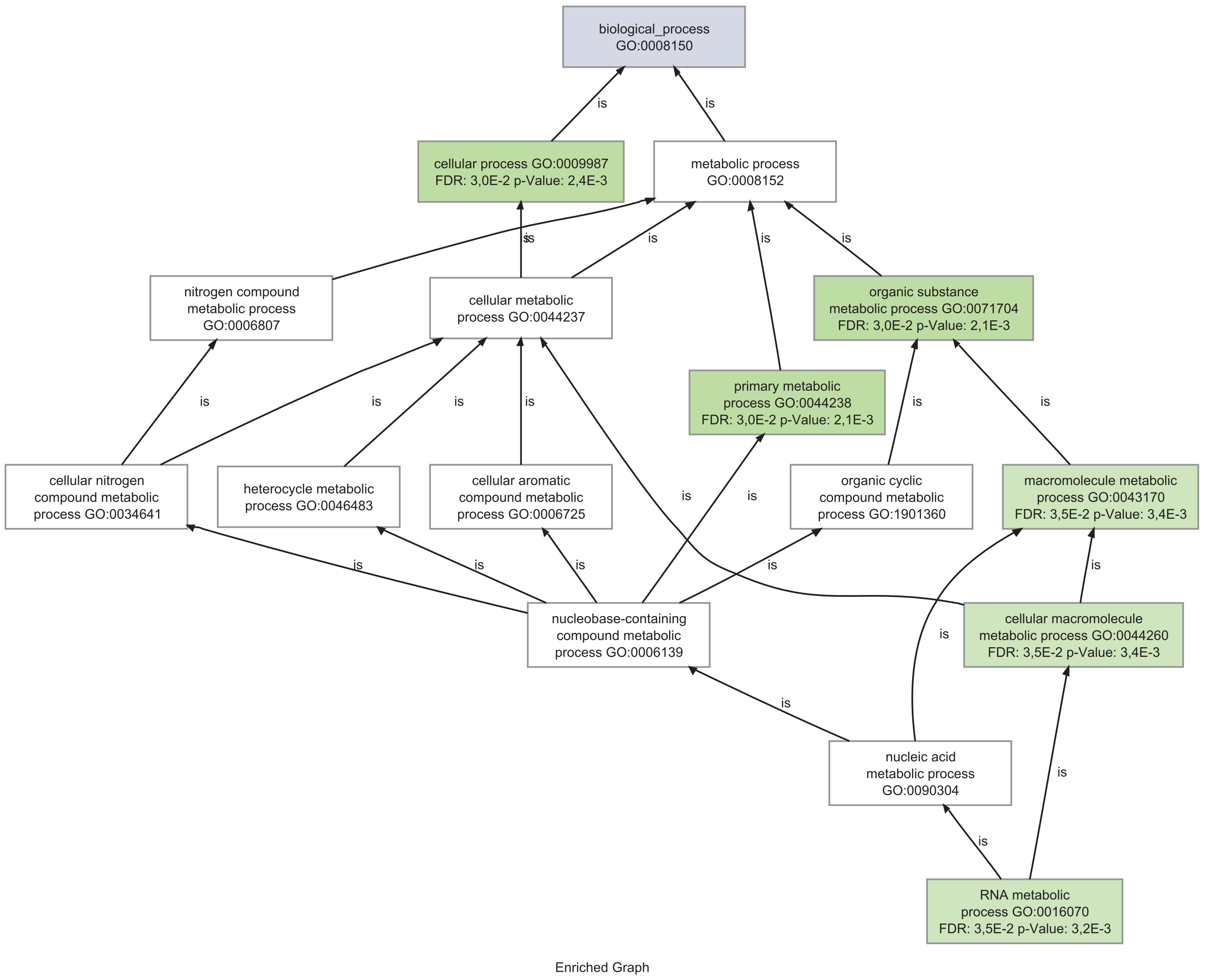

Figure S19. Genes, downegulated on methanol. Biological process
Graph explanation - same as on Figure S7.

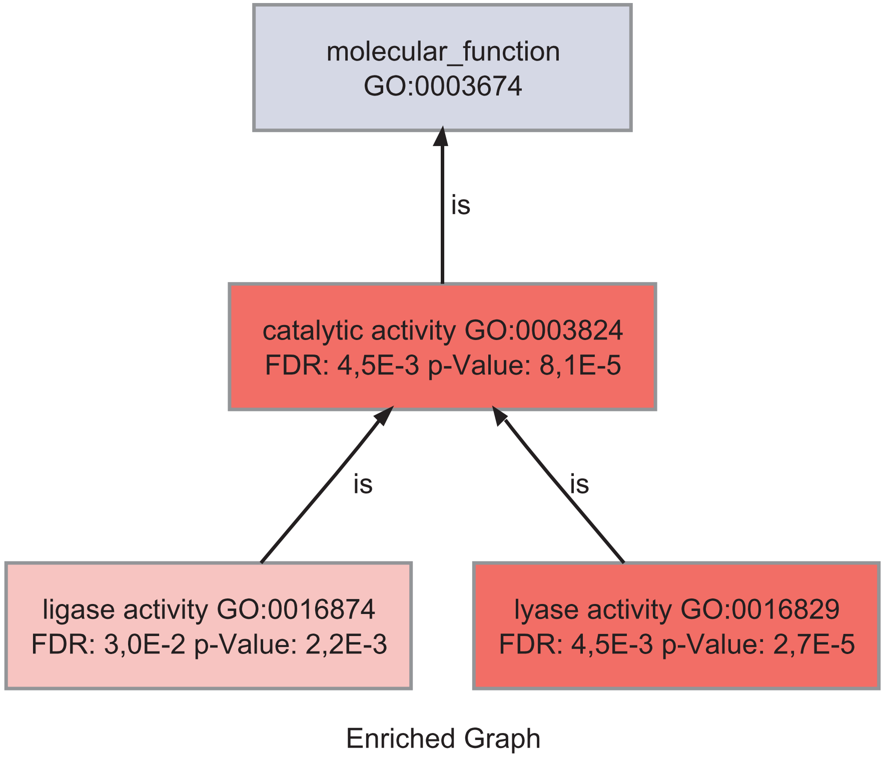

Figure S20. Genes, downregulated on methanol. Molecular function.
Graph explanation - same as on Figure S7.

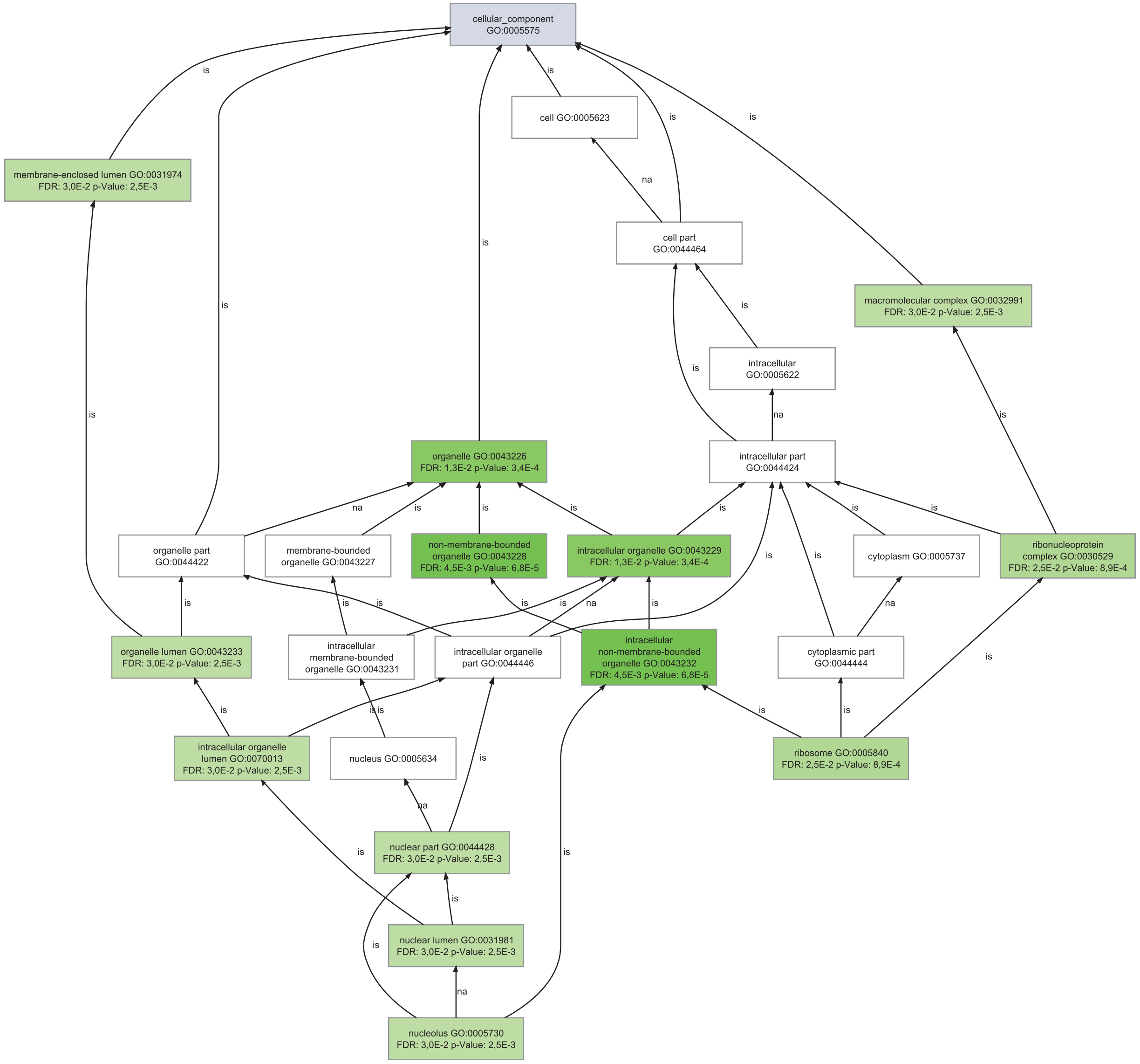

Enriched Graph

Figure S21. Genes, upregulated on methanol. Molecular function.
Graph explanation - as on Figure S7.

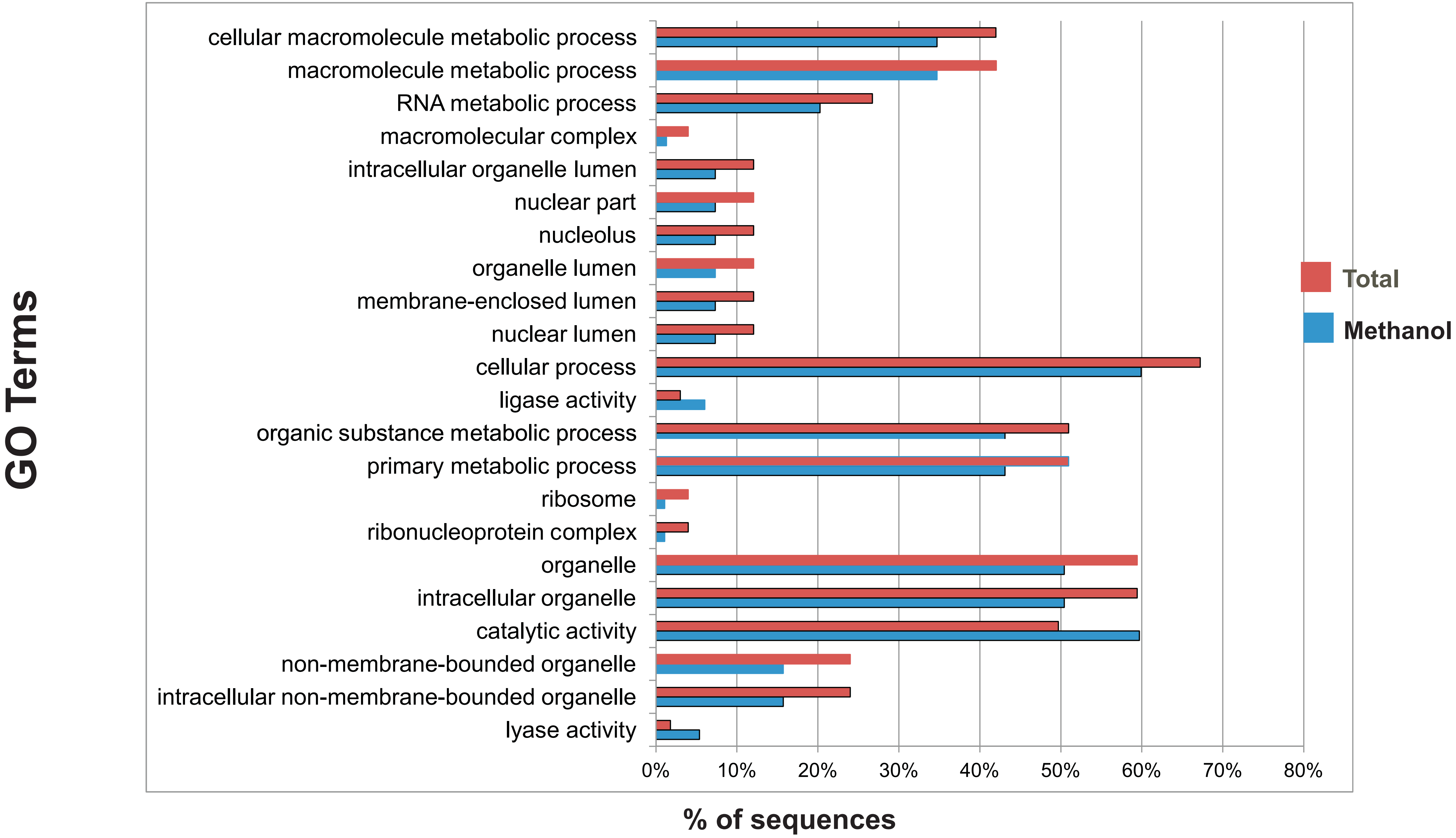

Figure S22.Differential GO term distribution.Genes downregulated on methanol.
Chart explanation - as on Figure S10.

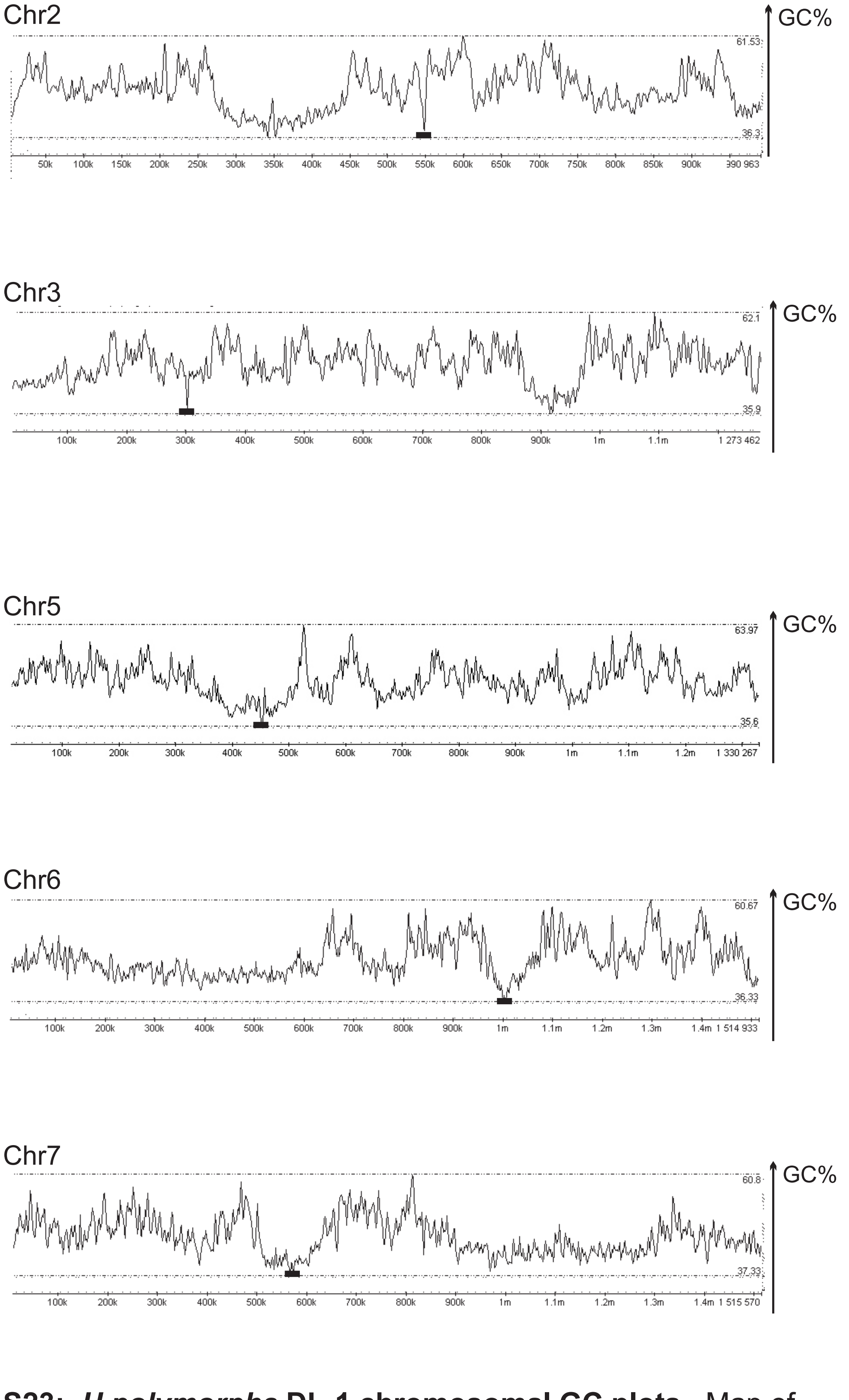

Figure S23: ***H.polymorpha*** **DL-1 chromosomal GC plots.** Map of calculated G+C content (ordinate) along 5 intact chromosomes with a 3000 bp sliding window and 100 bp step. Dashed lines indicate upper and lower G+C values. Proposed centromeres are indicated by filled bars.

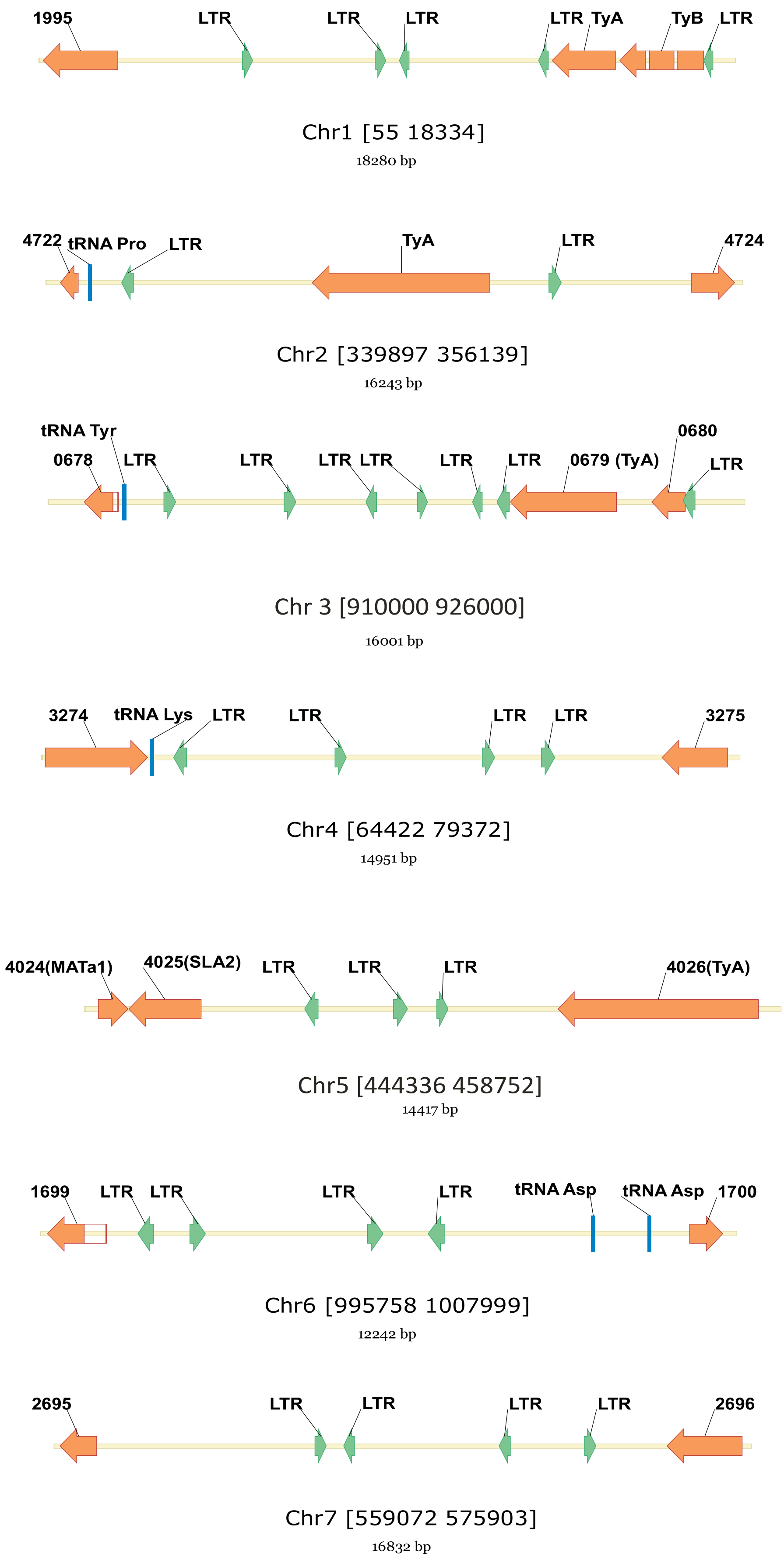

Figure S24: Clusters of LTR elements at proposed *H.polymorpha* DL-1 centromeric regions. LTRs - green arrows, numbers below maps correspond to chromosomal positions of corresponding loci, protein coding genes - orange arrows,numbers correspond to IDs of annotated genes.

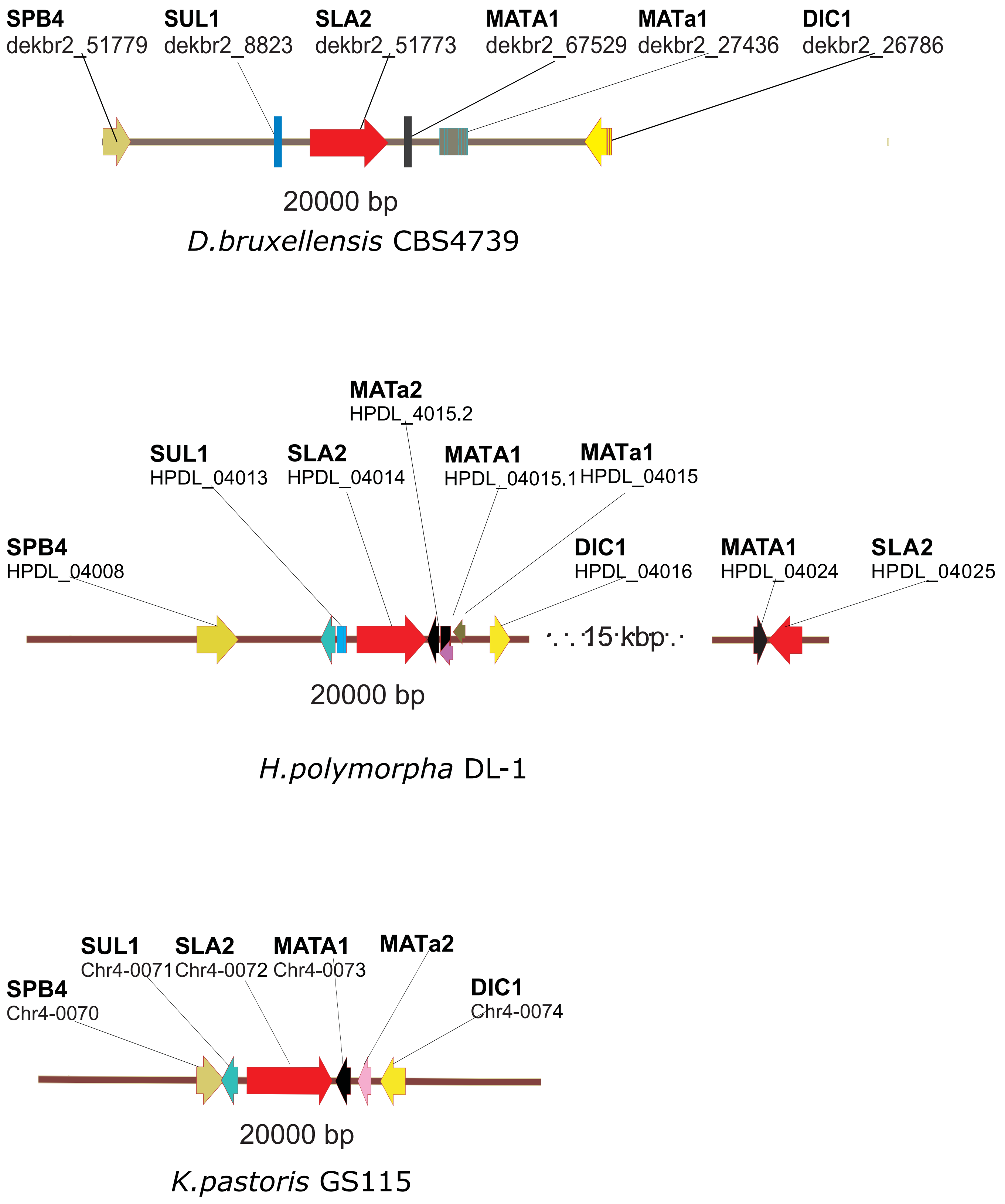

Figure S25. Comparative organization of MAT loci in the three yeast species. Genes are shown as arrowed boxes, identical coloring indicates ortology, gene names correspond to estasblished nomenclature of MAT loci associated genes (Butler et al., 2004). “DIC” gene encodes mitochondrial oxoglutarate/malate carrier protein, “SUL1”- eukaryotic translation initiation factor eIF-1, “SLA2”- Actin-binding protein SLA2/Huntingtin-interacting protein Hip1, “MATA1”- MATa1 transcription factor , ”MATa1” - MATalpha1 transcription factor, “MATa2” - MATalpha2 transcription factor, “SPB4” - ATP-dependent RNA helicase.

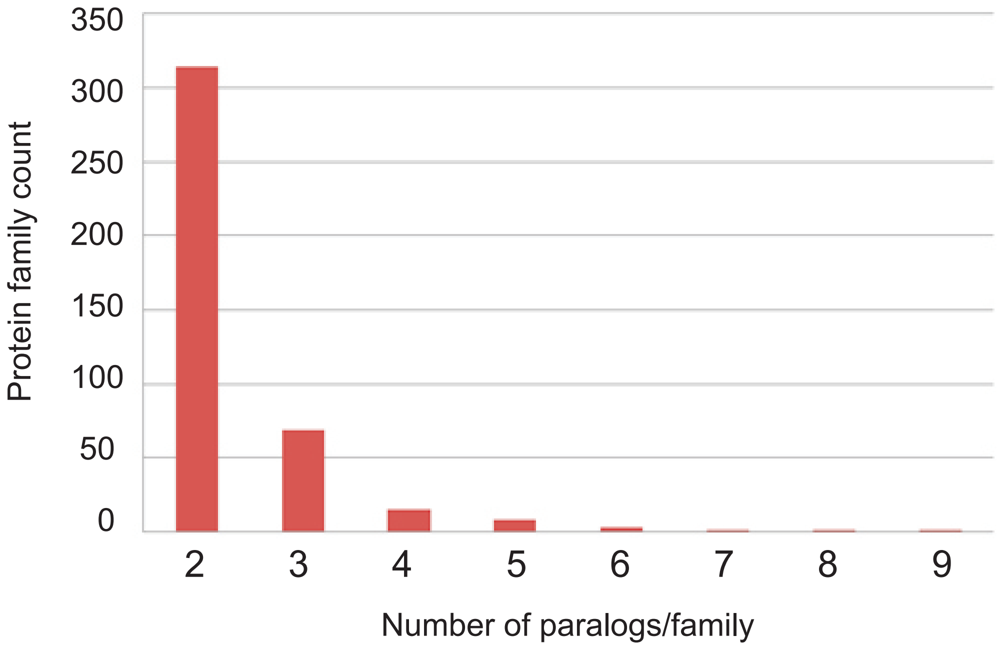

Figure S26. Evaluation of degree of paralogy in *H.polymorpha* genome. Distribution of *H.polymorpha* protein families identified using OrthoMCL cluster analysis according to the number of paralogs per family (X axis).

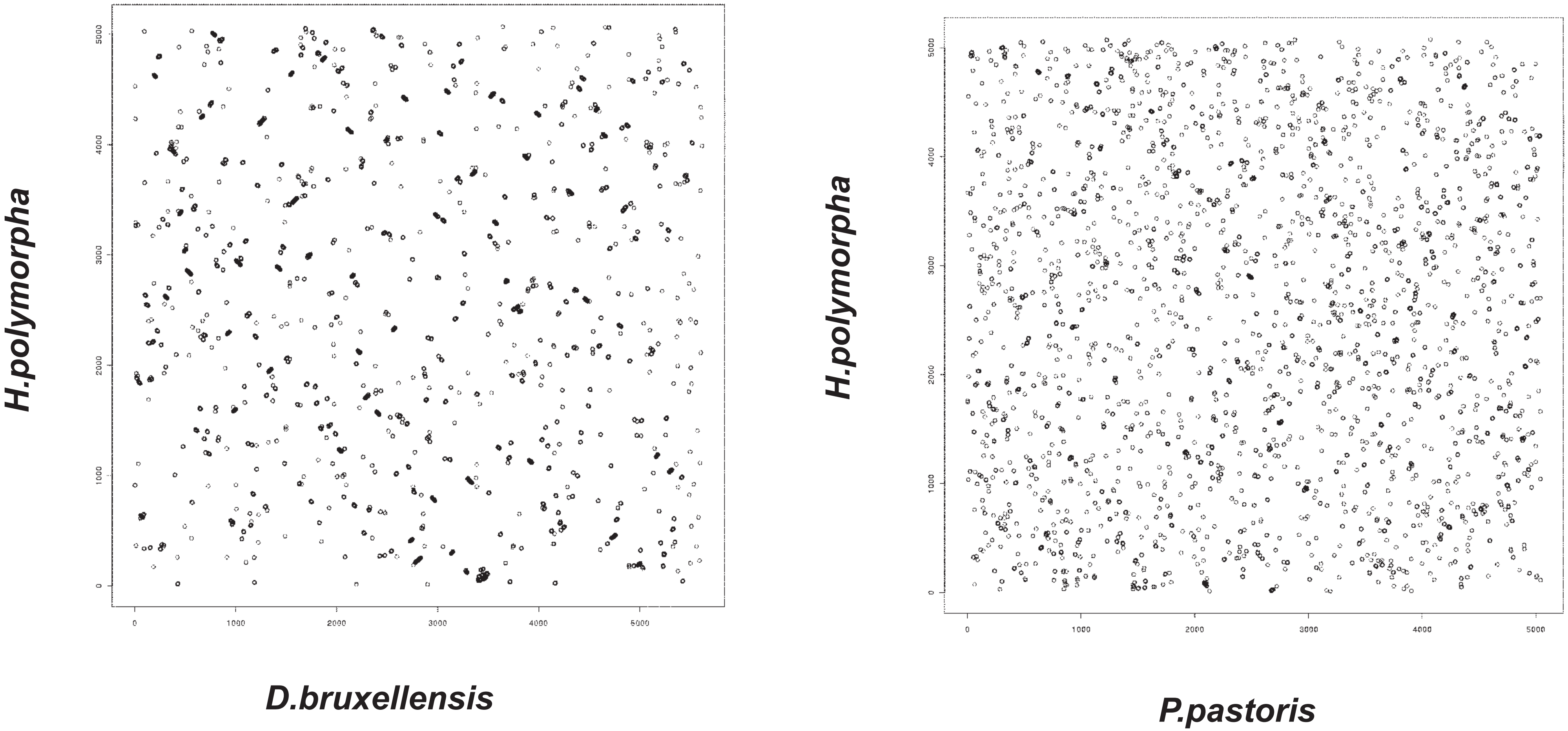

Figure S27. Genomic dot-plot for estimation of degree of synteny conservation in three yeast genomes.   .

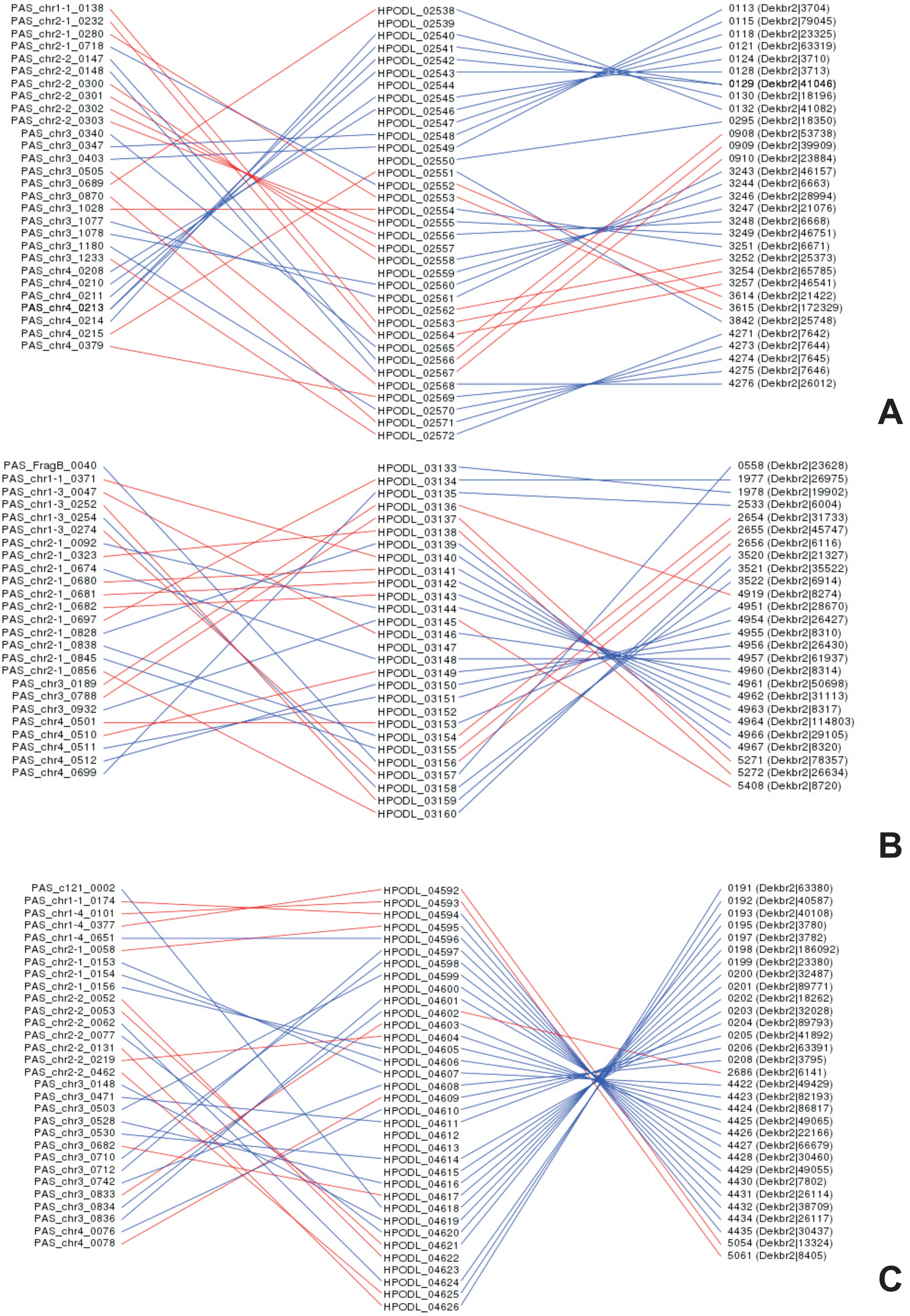

Figure S28. Synteny maps of H.polymorpha “MUT” loci (central column) and orthologous loci in D.bruxellensis (right column) and P.pastoris (left column) genomes
A- FLD locus B - FDH lous C- DAS .

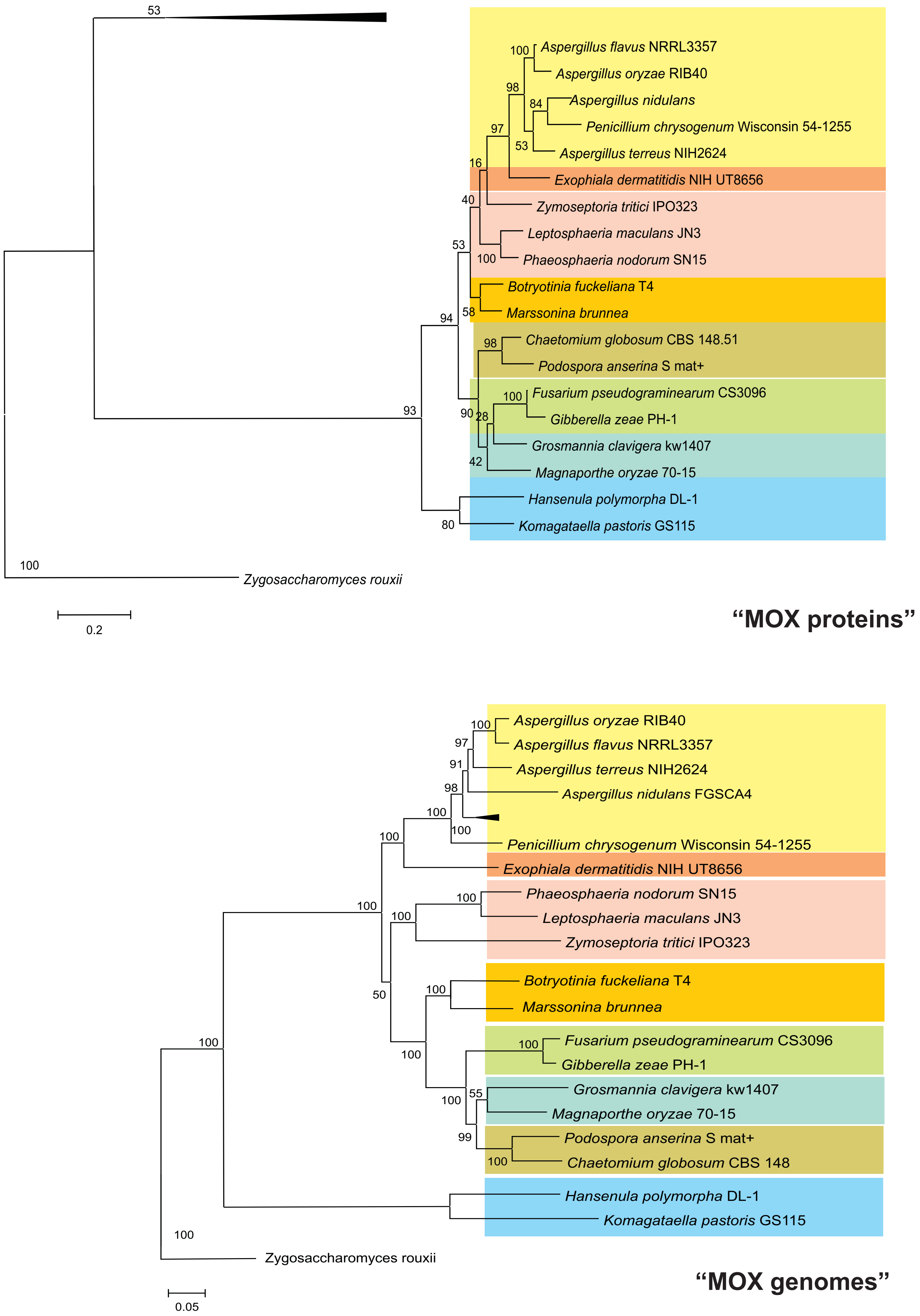

Figure S29. Comparison of phylogenetic trees of MOX proteins and phylogenomic trees of MOX genomes in Ascomycetes.

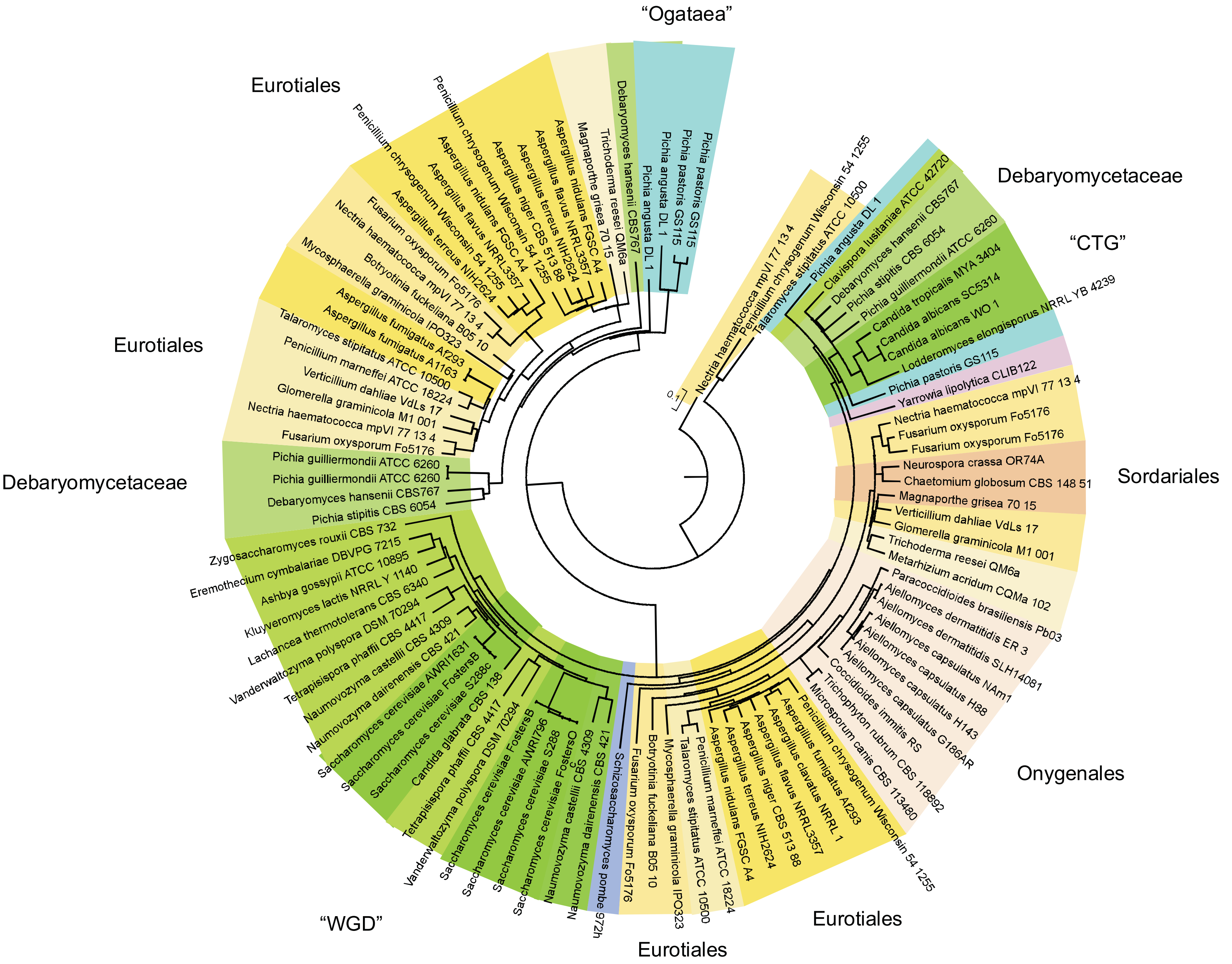

Figure S30. Phylogenetic tree of f DAS homologs in Ascomycetes Ortologs were identified in complete Ascomycetes genomes using BLAST search at NCBI Fungal BLAST page, obtained multiple alignments were realigned with COBALT and used to construct Newick trees online using Fast Minimum Evolution algorithm. Tree images were reformatted using MEGA tree editing options. Pezizomycotina clades are in yellow and brown colours, Saccharomycotina clades – in green.

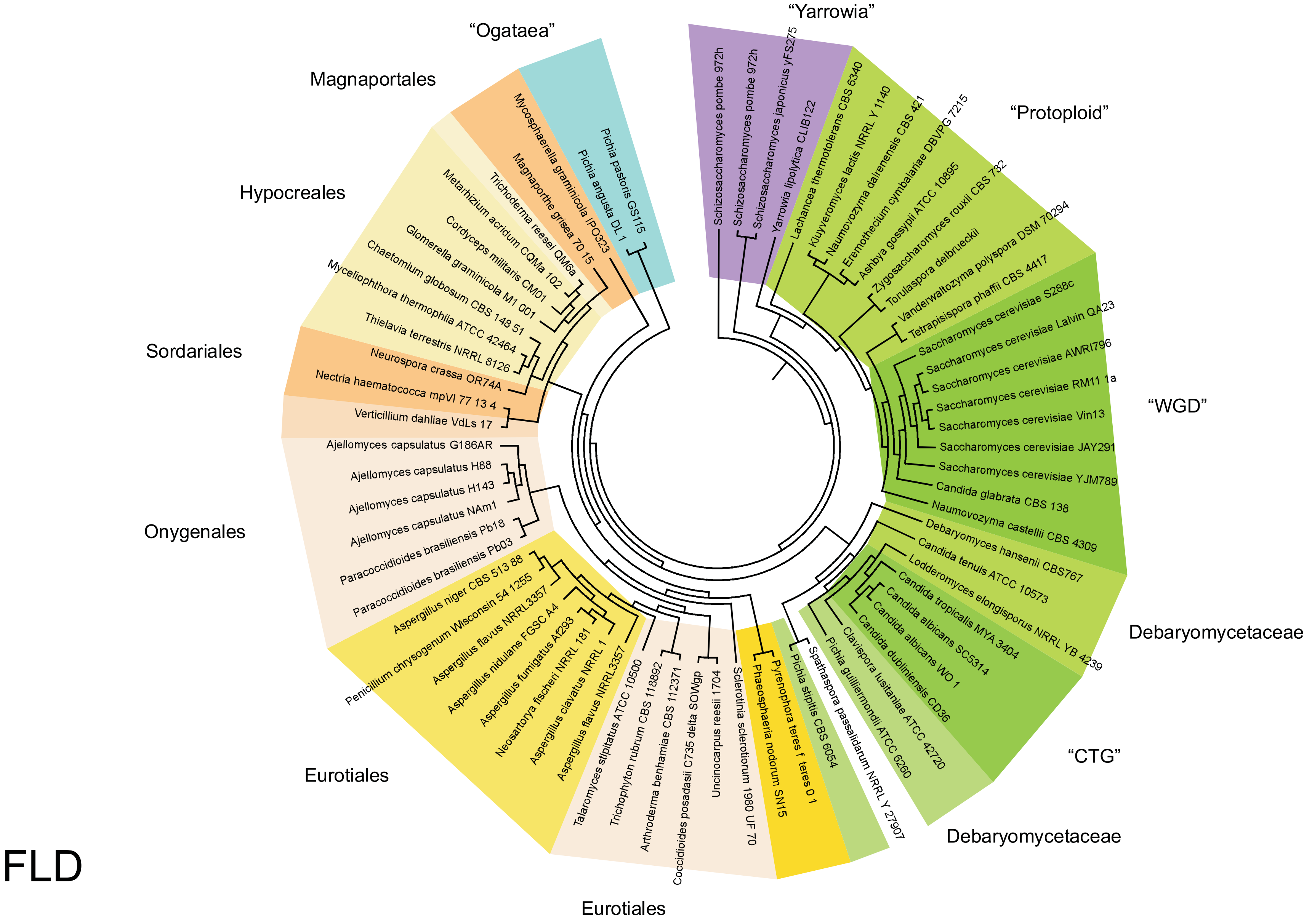

Figure S31. Phylogenetic tree of FLD orthologs in Ascomycetes.
Tree construction procedure and color code as on Fig.30

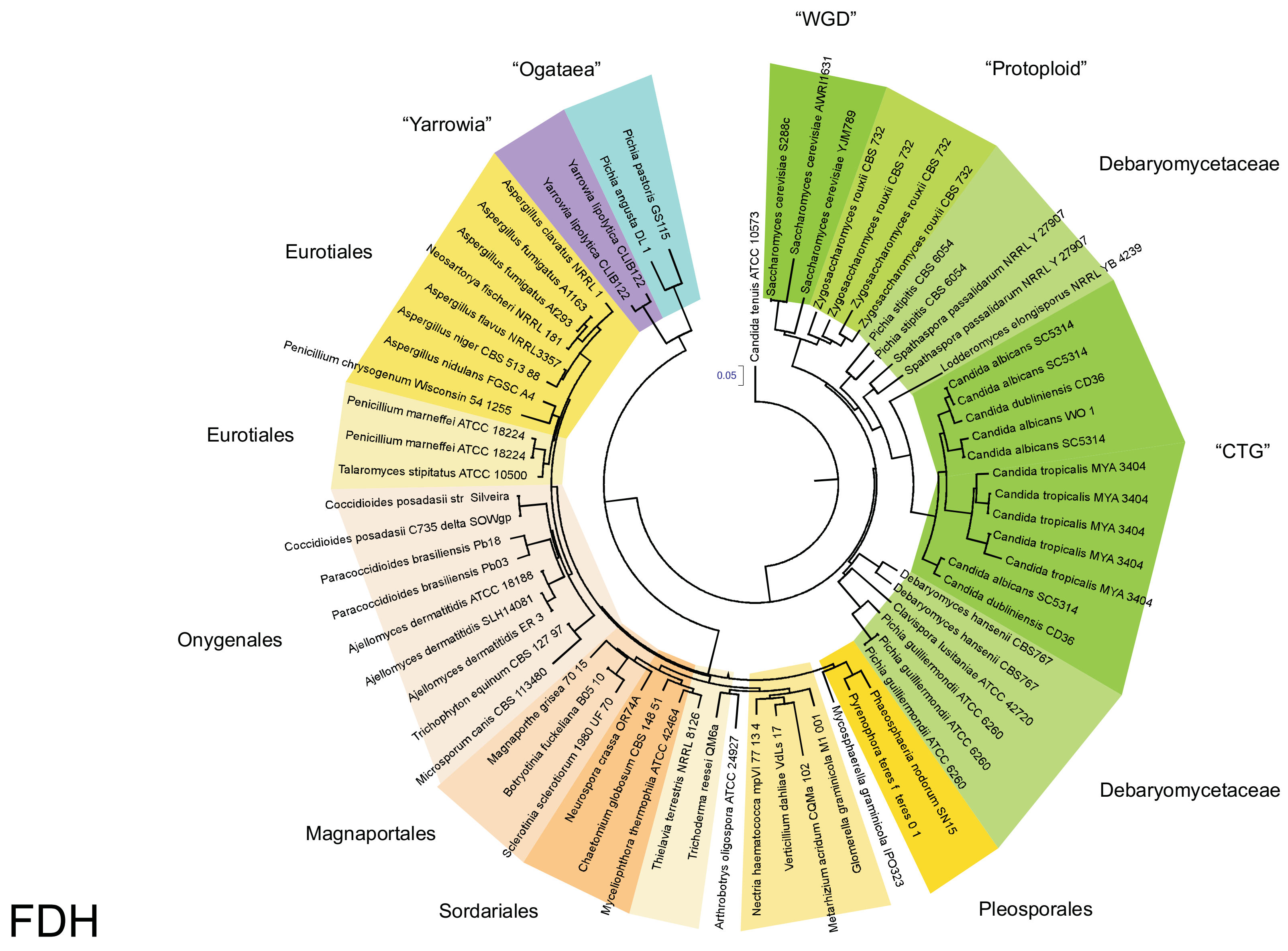

Figure S32. Phylogenetic tree of FDH orthologs in Ascomycetes.
Tree construction procedure and color code as on Fig.30
