# Supplementary material for: Genome sequence and analysis of methylotrophic yeast Hansenula polymorpha DL1
Source: BMC Genomics. 2013 Nov 27;14:837. doi: 10.1186/1471-2164-14-837 (PMC3866509; doi:10.1186/1471-2164-14-837)
Supplement: Additional file 2 — Supplementary tables. [file 1471-2164-14-837-S2.docx]

**Table S1. Differential expression of *H. polymorpha* DL-1 genes classified into KEGG groups**

| **KEGG groups** | **Total number of genes** | **Glucose-induced genes** | **No significant changes in expression** | **Methanol-induced genes** |
| --- | --- | --- | --- | --- |
|  |  | **(log2M/G<1)** | **1<log2M/G<1** | **(log2M/G>1)** |
| Metabolism |  |  |  |  |
| 1. Carbohydrate metabolism | **274** | **133** | **98** | **43** |
| 2. Energy metabolism | **149** | **18** | **45** | **86** |
| 3. Lipid metabolism | **129** | **13** | **38** | **78** |
| 4. Nucleotide metabolism | **159** | **26** | **61** | **72** |
| 5. Amino acid metabolism | **320** | **102** | **84** | **134** |
| 6. Glycan biosynthesis and metabolism | **83** | **13** | **42** | **28** |
| 7. Metabolism of cofactors and vitamins | **109** | **33** | **36** | **40** |
| 8. Biosynthesis of other secondary metabolites | **38** | **9** | **14** | **15** |
| 9. Xenobiotics biodegradation and metabolism | **83** | **8** | **31** | **44** |
| **Genetic Information Processing** |  |  |  |  |
| 10. Transcription | **129** | **26** | **68** | **35** |
| 11. Translation | **318** | **42** | **144** | **132** |
| 12. Folding, sorting and degradation | **238** | **24** | **135** | **79** |
| 13. Replication and repair | **145** | **16** | **59** | **70** |
| **Environmental Information Processing** |  |  |  |  |
| 14. Signal transduction | **197** | **23** | **104** | **70** |
| Cellular Processes |  |  |  |  |
| 15. Transport and catabolism | **130** | **22** | **44** | **64** |
| 16. Cell growth and death | **260** | **33** | **128** | **99** |

**Table S2. Expression levels of *H. polymorpha* DL-1 genes involved in glycolysis, gluconeogenesis and ethanol production**

| **Function** | **Gene ID** | **Normalized coverage (x10^6^)** | | **LOG2 M/G** | |
| --- | --- | --- | --- | --- | --- |
|  |  | **glucose** | **methanol** | **DL-1** | **NCYC**  **495*** |
| Glucokinase | HPODL_04126 | **914.89** | **385.33** | **-1.25** | **-1.20** |
| Hexokinase | HPODL_04936 | **75.62** | **298.41** | **1.98** | **-2.51** |
|  | HPODL_04428 | **7.41** | **15.93** | **1.10** |  |
| Glucose-6-phosphate isomerase | HPODL_01256 | **1147.69** | **460.65** | **-1.32** | **-0.76** |
| Phosphofructokinase | HPODL_00776 | **26.69** | **43.46** | **0.70** | **-1.53** |
|  | HPODL_01506 | **35.59** | **31.87** | **-0.16** | **-1.00** |
| Fructose-bisphosphate aldolase | HPODL_03669 | **2950.77** | **9988.08** | **1.76** | **1.07** |
| Triosephosphate isomerase | HPODL_03187 | **1054.27** | **5452.52** | **2.37** | **-0.58** |
| Glyceraldehyde-3-phosphate dehydrogenase | HPODL_04957 | **6849.04** | **3381.03** | **-1.02** | **-0.48** |
| Phosphoglycerate kinase | HPODL_04829 | **689.50** | **269.44** | **-1.36** | **-0.38** |
| Phosphoglycerate mutase | HPODL_01332 | **1539.14** | **967.66** | **-0.67** | **0.26** |
|  | HPODL_00350 | **2.97**** | **14.49** | **2.29** |  |
| Enolase | HPODL_04582 | **750.30** | **444.72** | **-0.75** | **-0.76** |
| Pyruvate kinase | HPODL_01513 | **1290.03** | **504.11** | **-1.36** | **-0.26** |
| Pyruvate carboxylase | HPODL_03803 | **391.46** | **286.82** | **-0.45** | **0.93** |
| Phosphoenolpyruvate carboxykinase | HPODL_00509 | **37.07** | **28.97** | **-0.36** | **3.15** |
| Fructose-1,6-bisphosphatase | HPODL_03321 | **47.45** | **711.26** | **3.91** | **2.14** |
| Alcohol dehydrogenase 2 | HPODL_03666 | **16724,47** | **21204,57** | **0,34** |  |
|  | HPODL_02528 | **22,24** | **553,36** | **4,64** |  |
| Pyruvate decarboxylase | HPODL_04375 | **2767.20** | **836.56** | **-1.73** | **-0.68** |
| Pyruvate dehydrogenase (subunit alpha) | HPODL_00436 | **224.20** | **300.15** | **0.42** | **-0.38** |
| Pyruvate dehydrogenase (subunit beta) | HPODL_01422 | **276.10** | **201.79** | **-0.45** | **-0.85** |
| Acetyl-CoA hydrolase | HPODL_04251 | **71,17** | **40,56** | **-0,81** |  |
| Acetyl-coenzyme A synthetase 1 | HPODL_02713 | **148,28** | **1771,63** | **3,58** |  |
| Acetyl-coenzyme A synthetase 2 | HPODL_02549 | **93,42** | **33,32** | **-1,49** |  |

* strain described in [27]

** means that 1 read was added to the gene coverage

**Table S3. Expression levels of *H. polymorpha* DL-1 methanol utilization pathway genes**

| **Function** | **Gene ID** | **Normalized coverage (x10^6^)** | | **LOG2 M/G** | |
| --- | --- | --- | --- | --- | --- |
|  |  | **glucose** | **methanol** | **DL-1** | **NCYC**  **495*** |
| Аlcohol oxidase | HPODL_03886 | **23.72** | **15669.48** | **9.37** | **4.11** |
| Сatalase | HPODL_04626 | **148.28** | **12011.77** | **6.34** | **5.42** |
| Dihydroxyacetone synthase | HPODL_04602 | **11.86** | **12304.39** | **10.02** | **4.25** |
| Dihydroxyacetone kinase | HPODL_04538 | **1.48**** | **756.17** | **8.99** | **1.54** |
|  | HPODL_01039 | **22.24** | **817.01** | **5.20** |  |
| Formaldehyde dehydrogenase | HPODL_02554 | **628.71** | **18455.13** | **4.88** | **3.07** |
| S-formylglutathione hydrolase | HPODL_03324 | **222.42** | **8423.59** | **5.24** | **1.14** |
| Formate dehydrogenase | HPODL_03145 | **10.38** | **19760.32** | **10.89** | **8.44** |

* strain described in [27]

** means that 1 read was added to the gene coverage

**Table S4. Expression levels of *H. polymorpha* DL-1 pentose phosphate pathway genes**

| **Function** | **Gene ID** | **Normalized coverage (x10^6^)** | | **LOG2 M/G** |
| --- | --- | --- | --- | --- |
|  |  | **glucose** | **methanol** | **DL-1** |
| Glucose-6-phosphate dehydrogenase | HPODL_03864 | 203.14 | 139.07 | **-0.55** |
| 6-phosphogluconolactonase | HPODL_03047 | 163.11 | 202.80 | **0.31** |
|  | HPODL_01158 | 45.97 | 153.55 | **1.74** |
| 6-phosphogluconate dehydrogenase | HPODL_02085 | 1521.35 | 1014.02 | **-0.59** |
| Ribose-5-phosphate isomerase | HPODL_02542 | 1.48 | 1070.51 | **9.50** |
|  | HPODL_02541 | 2.97 | 796.73 | **8.07** |
| Ribulose-phosphate 3-epimerase | HPODL_03355 | 17.79 | 408.50 | **4.52** |
| Transketolase | HPODL_04404 | 124.55 | 470.79 | **1.92** |
|  | HPODL_02162 | 1.48** | 10.14 | **2.77** |
| Transaldolase | HPODL_02007 | 1807.53 | 1668.78 | **-0.12** |
|  | HPODL_02415 | 2.97 | 693.88 | **7.87** |
|  | HPODL_02008 | 206.11 | 12741.86 | **5.95** |

** means that 1 read was added to the gene coverage

**Table S5. Expression levels of *H. polymorpha* DL-1 PEX genes**

| **Function** | **Gene ID** | **Normalized coverage (x10^6^)** | | **LOG2 M/G** | |
| --- | --- | --- | --- | --- | --- |
|  |  | **glucose** | **methanol** | **DL-1** | **NCYC**  **495*** |
| PEX1 | HPODL_03328 | 5.93 | 66.64 | **3.49** | **1.54** |
| PEX2 | HPODL_04254 | 7.41 | 325.93 | **5.46** | **0.76** |
| PEX3 | HPODL_03618 | 56.35 | 696.78 | **3.63** | **0.58** |
| PEX4 | HPODL_02596 | 10.38 | 28.97 | **1.48** | **1.63** |
| PEX5 | HPODL_00015 | 4.45 | 223.08 | **5.65** | **1.72** |
| PEX6 | HPODL_01223 | 16.31 | 252.06 | **3.95** | **0.68** |
| PEX7 | HPODL_03008 | 29.66 | 40.56 | **0.45** | **0.85** |
| PEX8 | HPODL_04665 | 16.31 | 175.28 | **3.43** | **0.77** |
| PEX10 | HPODL_00444 | 32.62 | 155.00 | **2.25** | **0.68** |
| PEX11 | HPODL_04241 | 41.52 | 5109.20 | **6.94** | **2.23** |
| PEX11C | HPODL_00505 | 32.62 | 55.05 | **0.75** | **-0.68** |
| PEX12 | HPODL_01617 | 8.90 | 76.78 | **3.11** | **1.38** |
| PEX13 | HPODL_02269 | 2.97 | 317.24 | **6.74** | **1.58** |
| PEX14 | HPODL_04224 | 20.76 | 298.41 | **3.85** | **1.81** |
| PEX17 | HPODL_02666 | 19.28 | 453.41 | **4.56** | **1.07** |
| PEX19 | HPODL_01942 | 23.72 | 169.49 | **2.84** | **0.14** |
| PEX20 | HPODL_02412 | 41.52 | 30.42 | **-0.45** | **0.00** |
| PEX22 | HPODL_01386 | 60.79 | 131.82 | **1.12** | **-0.38** |
| PEX23 | HPODL_01523 | 45.97 | 56.50 | **0.30** | **0.26** |
| PEX24 | HPODL_03516 | 419.63 | 105.75 | **-1.99** | **1.54** |
| PEX25 | HPODL_03136 | 25.21 | 433.13 | **4.10** | **1.14** |
| PEX26 | HPODL_02690 | 351.42 | 589.58 | **0.75** | **1.85** |
| PEX29 | HPODL_00080 | 10.38 | 94.16 | **3.18** | **-0.26** |
| PEX32 | HPODL_04641 | 14.83 | 10.14 | **-0.55** | **2.26** |

* strain described in [27]

**Table S6. Expression levels of *H. polymorpha* DL-1 autophagy-related genes**

| **Function** | **Gene ID** | **Normalized coverage (x10^6^)** | | **LOG2 M/G** | |
| --- | --- | --- | --- | --- | --- |
|  |  | **glucose** | **methanol** | **DL-1** | **NCYC495*** |
| ATG1 | HPODL_04379 | 172,00 | 33,32 | **-2,37** | **1,89** |
| ATG2 | HPODL_03060 | 191,28 | 59,39 | **-1,69** | **1,38** |
| ATG3 | HPODL_01803 | 17,79 | 42,01 | **1,24** | **1,96** |
| ATG4 | HPODL_04394 | 5,93 | 10,14 | **0,77** | **1,20** |
| ATG5 | HPODL_03429 | 8,90 | 15,93 | **0,84** | **0,85** |
| ATG6 | HPODL_04150 | 557,53 | 47,80 | **-3,54** | **1,89** |
| ATG7 | HPODL_03851 | 5,93 | 26,07 | **2,14** | **1,14** |
| ATG8 | HPODL_03718 | 99,35 | 392,57 | **1,98** | **2,63** |
| ATG9 | HPODL_03149 | 10,38 | 15,93 | **0,62** | **0,68** |
| ATG10 | HPODL_05255 | 1,48** | 7,24 | **2,29** | **1,32** |
| ATG11 | HPODL_05134 | 4,45 | 20,28 | **2,19** | **2,38** |
| ATG12 | HPODL_01112 | 1,48** | 4,35 | **1,55** | **1,20** |
| ATG13 | HPODL_03959 | 131,97 | 36,21 | **-1,87** | **0,68** |
| ATG15 | HPODL_03503 | 80,07 | 147,76 | **0,88** | **1,14** |
| ATG16 | HPODL_05261 | 35,59 | 43,46 | **0,29** | **0,77** |
| ATG17 | HPODL_00301 | 1,48** | 18,83 | **3,67** | **0,14** |
| ATG18 | HPODL_04964 | 48,93 | 43,46 | **-0,17** | **0,93** |
| ATG19-like | HPODL_02613 | 68,21 | 126,03 | **0,89** | **2,72** |
| ATG20 | HPODL_03229 | 20,76 | 40,56 | **0,97** | **-0,14** |
| ATG21 | HPODL_01040 | 1,48** | 17,38 | **3,55** | **0,85** |
| ATG22 | HPODL_03790 | 16,31 | 130,37 | **3,00** | **0,77** |
| ATG24 | HPODL_01247 | 292,11 | 141,96 | **-1,04** | **1,49** |
| ATG25 | HPODL_01515 | 7,41 | 27,52 | **1,89** | **-2,10** |
| ATG26 | HPODL_02824 | 19,28 | 70,98 | **1,88** | **1,07** |
| ATG27 | HPODL_01559 | 238,73 | 214,39 | **-0,16** | **-0,77** |
| ATG28 | HPODL_03555 | 51,90 | 173,83 | **1,74** | **0,85** |
| ATG30 | HPODL_02312 | 203,14 | 59,39 | **-1,77** | **2,04** |

* strain described in [27]

** means that 1 read was added to the gene coverage

**Table S7. Expression levels of *H. polymorpha* DL-1 antioxidant system genes**

| **Function** | **Gene ID** | **Normalized coverage (x10^6^)** | | **LOG2 M/G** | |
| --- | --- | --- | --- | --- | --- |
|  |  | **glucose** | **methanol** | **DL-1** | **NCYC**  **495*** |
| Catalase | HPODL_04626 | 148.28 | 12011.77 | **6.34** | **5,42** |
| Superoxide dismutase (Mn) | HPODL_01414 | 20.76 | 89.81 | **2.11** | **2,1** |
|  | HPODL_02458 | 533.81 | 2507.52 | **2.23** |  |
|  | HPODL_03894 | 11.86 | 104.30 | **3.14** |  |
| Superoxide dismutase (Cu-Zn) | HPODL_01412 | 1768.98 | 185.42 | **-3.25** |  |
| Peroxiredoxin | HPODL_03023 | 69.69 | 1253.04 | **4.17** | **3,46** |
|  | HPODL_00398 | 44.48 | 237.57 | **2.42** |  |
|  | HPODL_02878 | 2236.06 | 4916.54 | **1.14** |  |
|  | HPODL_03404 | 4.45 | 2627.76 | **9.21** | **5,56** |
|  | HPODL_00527 | 72.66 | 1507.99 | **4.38** |  |
|  | HPODL_03659 | 7.41 | 39.11 | **2.40** |  |
|  | HPODL_00254 | 1.48** | 292.62 | **7.62** |  |
| Thioredoxin | HPODL_03852 | 1.48 | 4.35 | **1.55** |  |
|  | HPODL_01485 | 22.24 | 23.18 | **0.06** |  |
| Thioredoxin reductase | HPODL_04814 | 40.04 | 521.50 | **3.70** | **3,38** |
| Glutaredoxin | HPODL_00292 | 1.48 | 13.04 | **3.14** | **1,5** |
|  | HPODL_04165 | 5.93 | 14.49 | **1.29** |  |
|  | HPODL_02727 | 1.48 | 23.18 | **3.97** |  |
| Glutathione S-transferase | HPODL_02388 | 91.93 | 964.77 | **3.39** | **1,38** |
|  | HPODL_03063 | 741.40 | 525.84 | **-0.50** |  |
| Glutathione reductase | HPODL_01177 | 269.87 | 320.14 | **0.25** | **3** |
| Glutathione peroxidase | HPODL_03940 | 338.08 | 31.87 | **-3.41** |  |
| Gamma-glutamylcysteine synthetase | HPODL_02265 | 47.45 | 65.19 | **0.46** |  |
| Glutathione synthetase | HPODL_00931 | 10.38 | 70.98 | **2.77** |  |

* strain described in [27]

** means that 1 read was added to the gene coverage **Table S8. Expression levels of *H. polymorpha* DL-1 fatty acid beta-oxidation genes**

| **Function** | **Gene ID** | **Normalized coverage (x10^6^)** | | **LOG2M/G** | |
| --- | --- | --- | --- | --- | --- |
|  |  | **glucose** | **methanol** | **DL-1** | **NCYC**  **495*** |
| Peroxisomal ABC-transporter sub-unit 1 | HPODL_01261 | 145.31 | 115.89 | **-0.33** | **3.00** |
| Peroxisomal ABC-transporter sub-unit 2 | HPODL_00217 | 173.49 | 317.24 | **0.87** | **2.68** |
| Acyl-coenzyme A oxidase | HPODL_01143 | 28.17 | 385.33 | **3.77** | **3.81** |
| Multifunctional enzyme | HPODL_00315 | 25.21 | 156.45 | **2.63** | **4.43** |
| 3-ketoacyl-CoA thiolase | HPODL_04502 | 1.48** | 102.85 | **6.12** | **4.03** |
| Catalase | HPODL_04626 | 148.28 | 12011.77 | **6.34** | **5.42** |
| Isocitrate lyase | HPODL_00142 | 11.86 | 18.83 | **0.67** | **4.94** |
|  | HPODL_01751 | 13.35 | 4.35 | **-1.62** |  |
|  | HPODL_01752 | 22.24 | 13.04 | **-0.77** |  |
| Malate synthase | HPODL_01838 | 83.04 | 60.84 | **-0.45** | **3.14** |
| Isocitrate dehydrogenase | HPODL_01327 | 1.48 | 110.09 | **6.21** | **4.74** |
|  | HPODL_03428 | 53.38 | 204.25 | **1.94** |  |
|  | HPODL_01696 | 268.39 | 521.50 | **0.96** |  |
|  | HPODL_04851 | 177.94 | 52.15 | **-1.77** |  |
|  | HPODL_03527 | 1107.65 | 136.17 | **-3.02** |  |
| Carnitine acetyltransferase | HPODL_03642 | 37.07 | 231.78 | **2.64** | **5.38** |
|  | HPODL_00251 | 4.45 | 143.41 | **5.01** | **5.56** |
|  | HPODL_01480 | 1.48 | 18.83 | **3.67** | **5.17** |
| Mitochondrial carnitine carrier | HPODL_02892 | 1.48 | 323.04 | **7.77** | **6.8** |
| peroxisomal 2,4-dienoyl-CoA reductase | HPODL_04580 | 2.97 | 31.87 | **3.43** | **1.49** |
|  | HPODL_02860 | 74.14 | 440.37 | **2.57** |  |
| fatty acyl-CoA synthetase | HPODL_02909 | 295.08 | 1707.90 | **2.53** | **0.85** |

* strain described in [27]

** means that 1 read was added to the gene coverage

**Table S9. Expression levels of selected *H. polymorpha* DL-1 transcription factor genes ***

| **Function** | **Gene ID** | **GO terms** | **Interpro**  **domains** | **Normalized coverage (x10^6^)** | | **LOG2 M/G** |
| --- | --- | --- | --- | --- | --- | --- |
|  |  |  |  | **GLU** | **MET** |  |
| Putative transcription factor SEF1 | HPODL_03233 | GO:0003700 sequence-specific DNA binding transcription factor activity; GO:0005634 nucleus; GO:0006355 regulation of transcription, DNA-dependent; GO:0008270 zinc ion binding | IPR001138 Zn(2)-C6 fungal-type DNA-binding domain | **1,48** | **204,25** | **7,11** |
| Regulatory protein ADR1^[[1]](#footnote-1)^ | HPODL_00650 | GO:0003676 nucleic acid binding | IPR013087 Zinc finger C2H2-type/integrase DNA-binding domain | **2,97** | **230,33** | **6,28** |
| MPP1 | HPODL_04601 | GO:0003700 sequence-specific DNA binding transcription factor activity; GO:0005634 nucleus; GO:0006355 regulation of transcription, DNA-dependent; GO:0008270 zinc ion binding | IPR001138 Zn(2)-C6 fungal-type DNA-binding domain | **7,41** | **392,57** | **5,73** |
| hypothetical protein | HPODL_04541 | GO:0003700 sequence-specific DNA binding transcription factor activity; GO:0005634 nucleus; GO:0006355 regulation of transcription, DNA-dependent; GO:0008270 zinc ion binding | IPR001138 Zn(2)-C6 fungal-type DNA-binding domain | **4,45** | **210,05** | **5,56** |
| Transcriptional activator protein acu-15 | HPODL_02294 | GO:0003700 sequence-specific DNA binding transcription factor activity; GO:0005634 nucleus; GO:0006355 regulation of transcription, DNA-dependent; GO:0008270 zinc ion binding | IPR001138 Zn(2)-C6 fungal-type DNA-binding domain | **1,48** | **57,94** | **5,29** |
| hypothetical protein | HPODL_03024 | GO:0003677 DNA binding; GO:0003700 sequence-specific DNA binding transcription factor activity; GO:0006355 regulation of transcription, DNA-dependent | IPR001138 Zn(2)-C6 fungal-type DNA-binding domain | **1,48** | **20,28** | **3,77** |
| hypothetical protein | HPODL_05042 | GO:0003700 sequence-specific DNA binding transcription factor activity; GO:0006355 regulation of transcription, DNA-dependent; GO:0043565 sequence-specific DNA binding | IPR004827 Basic-leucine zipper domain | **4,45** | **59,39** | **3,74** |
| Transcriptional repressor XBP1 | HPODL_03402 | GO:0003677 DNA binding; GO:0003700 sequence-specific DNA binding transcription factor activity; GO:0006355 regulation of transcription, DNA-dependent | IPR003163 Transcription regulator HTH, APSES-type DNA-binding domain | **1,48** | **17,38** | **3,55** |
| hypothetical protein | HPODL_01491 | GO:0003700 sequence-specific DNA binding transcription factor activity; GO:0006355 regulation of transcription, DNA-dependent; GO:0043565 sequence-specific DNA binding | IPR004827 Basic-leucine zipper domain | **8,90** | **98,50** | **3,47** |
| Enhanced filamentous growth protein | HPODL_01835 | GO:0003677 DNA binding; GO:0003700 sequence-specific DNA binding transcription factor activity; GO:0006355 regulation of transcription, DNA-dependent | IPR003163 Transcription regulator HTH, APSES-type DNA-binding domain | **10,38** | **92,71** | **3,16** |
| DNA-binding protein RAP1 | HPODL_03159 | GO:0005515 protein binding | IPR009057 Homeodomain-like | **38,55** | **166,59** | **2,11** |
| hypothetical protein | HPODL_02840 | GO:0003677 DNA binding; GO:0003700 sequence-specific DNA binding transcription factor activity; GO:0006355 regulation of transcription, DNA-dependent | IPR001138 Zn(2)-C6 fungal-type DNA-binding domain | **363,29** | **1144,39** | **1,66** |
| member of the Gal4p family of zinc cluster proteins^[[2]](#footnote-2)^ | HPODL_02522 | GO:0003677 DNA binding; GO:0005634 nucleus; GO:0006351 transcription, DNA-dependent; GO:0008270 zinc ion binding | IPR007219 Transcription factor, fungi | **22,24** | **39,11** | **0,81** |
| transcription factor SKN7 | HPODL_02729 | GO:0000156 phosphorelay response regulator activity; GO:0000160 phosphorelay signal transduction system; GO:0003700 sequence-specific DNA binding transcription factor activity; GO:0005634 nucleus; GO:0006355 regulation of transcription, DNA-dependent; GO:0006950 response to stress | IPR014402 Signal transduction response regulator, SKN7-like | **93,42** | **111,54** | **0,26** |
| Zinc cluster transcriptional activator^[[3]](#footnote-3)^ | HPODL_04955 | GO:0003677 DNA binding; GO:0005634 nucleus; GO:0006351 transcription, DNA-dependent; GO:0008270 zinc ion binding | IPR007219 Transcription factor, fungi | **60,79** | **57,94** | **-0,07** |
| General control protein GCN4 | HPODL_00153 | GO:0003700 sequence-specific DNA binding transcription factor activity; GO:0006355 regulation of transcription, DNA-dependent; GO:0043565 sequence-specific DNA binding | IPR004827 Basic-leucine zipper domain | **19737,52** | **4286,40** | **-2,20** |
| Transcriptional activator HAC1 | HPODL_01520 | GO:0003700 sequence-specific DNA binding transcription factor activity; GO:0006355 regulation of transcription, DNA-dependent; GO:0043565 sequence-specific DNA binding | IPR004827 Basic-leucine zipper domain | **821,47** | **137,62** | **-2,58** |
| Nitrogen regulatory protein GLN3 | HPODL_04003 | GO:0003700 sequence-specific DNA binding transcription factor activity; GO:0006355 regulation of transcription, DNA-dependent; GO:0008270 zinc ion binding | IPR013088 Zinc finger, NHR/GATA-type | **676,16** | **75,33** | **-3,17** |
| zinc cluster transcription factor, putative | HPODL_04995 | GO:0003700 sequence-specific DNA binding transcription factor activity; GO:0005634 nucleus; GO:0006355 regulation of transcription, DNA-dependent; GO:0008270 zinc ion binding | IPR001138 Zn(2)-C6 fungal-type DNA-binding domain | **634,64** | **39,11** | **-4,02** |
| hypothetical protein | HPODL_02684 | GO:0003700 sequence-specific DNA binding transcription factor activity; GO:0005634 nucleus; GO:0006355 regulation of transcription, DNA-dependent; GO:0008270 zinc ion binding | IPR001138 Zn(2)-C6 fungal-type DNA-binding domain | **539,74** | **21,73** | **-4,63** |
| hypothetical protein | HPODL_01452 | GO:0003700 sequence-specific DNA binding transcription factor activity; GO:0006355 regulation of transcription, DNA-dependent; GO:0043565 sequence-specific DNA binding | IPR004827 Basic-leucine zipper domain | **1097,27** | **40,56** | **-4,76** |
| Fluconazole resistance protein 1 | HPODL_01831 | GO:0003700 sequence-specific DNA binding transcription factor activity; GO:0005634 nucleus; GO:0006355 regulation of transcription, DNA-dependent; GO:0008270 zinc ion binding | IPR001138 Zn(2)-C6 fungal-type DNA-binding domain | **243,18** | **7,24** | **-5,07** |
| hypothetical protein | HPODL_01110 | GO:0003700 sequence-specific DNA binding transcription factor activity; GO:0005634 nucleus; GO:0006355 regulation of transcription, DNA-dependent; GO:0008270 zinc ion binding | IPR001138 Zn(2)-C6 fungal-type DNA-binding domain | **124,55** | **1,45** | **-6,43** |

*** Light-green colored cells – genes, upregulated on methanol, pink-colored cells – genes, downregulated on methanol, uncoloured cells – constitutively-expressed genes**

**Table S10. Expression levels of *H. polymorpha* DL-1 specific metabolic genes.**

| **Function** | **Gene ID** | **Normalized coverage (x10^6^)** | | **LOG2 M/G** |
| --- | --- | --- | --- | --- |
|  |  | **glucose** | **methanol** | **DL-1** |
| **Nitrate utilization** |  |  |  |  |
| YNA1 | HPODL_02383 | 1057.2 | 62.3 | -4.09 |
| nitrate reductase [NADPH] | HPODL_02384 | 13.3 | 56.5 | 2.1 |
| YNA2 | HPODL_02385 | 11.9 | 23.2 | 0.97 |
| nitrite reductase subunit NirD | HPODL_02386 | 14.8 | 28.9 | 0.97 |
| high affinity nitrate transporter NrtB | HPODL_02387 | 3 | 71 | 4.58 |
| Maltose assimilation |  |  |  |  |
| Sucrose utilization protein SUC1 | HPODL_02604 | 22.24 | 60.84 | 1.45 |
| MAL activator1 | HPODL_02605 | 20.76 | 46.35 | 1.16 |
| General alpha-glucoside permease | HPODL_02606 | 32.62 | 1589.11 | 5.61 |
| Alpha-glucosidase | HPODL_02607 | 54.86 | 1583.32 | 4.85 |
| Trehalose metabolism |  |  |  |  |
| Neutral trehalase | HPODL_03898 | 56.35 | 17.38 | -1.7 |
| Acid trehalase required for utilization of extracellular trehalose | HPODL_04314 | 13.35 | 26.07 | 0.97 |
| Arabinose assimilation |  |  |  |  |
| D-arabinose 1-dehydrogenase | HPODL_00314 | 65.24 | 147.76 | 1.18 |
| D-arabinose dehydrogenase [NAD(P)+] heavy chain | HPODL_02625 | 130.48 | 137.62 | 0.08 |
| **Xylose and cellobiose utilization** |  |  |  |  |
| Xylulose kinase | HPODL_01145 | 397.39 | 257.85 | -0.62 |
| Xylitol dehydrogenase (sorbitol dehydrogenase) | HPODL_01148 | 434.46 | 731.54 | 0.75 |
| Xylitol dehydrogenase (sorbitol dehydrogenase2) | HPODL_00131 | 28.17 | 466.45 | 4.05 |
| NADPH-dependent D-xylose reductase | HPODL_01370 | 32.61 | 6001.54 | 7.52 |
| Beta-glucosidase J | HPODL_03777 | 37.07 | 10.14 | -1.87 |
| Beta-glucosidase I | HPODL_05055 | 26.67 | 36.21 | 0.44 |
| Beta-glucosidase I | HPODL_01992 | 14.83 | 17.38 | 0.23 |

# Table S11. Retained introns in *H. polymorpha* DL1 genome.

| **chromosome** | **intron** | **start** | **end** | **strand** | **gene** |
| --- | --- | --- | --- | --- | --- |
| **chr1-1** | intron | 82120 | 82368 | - | HPODL_00038 |
| **chr1-1** | intron | 168799 | 168833 | - | HPODL_00081 |
| **chr1-3** | intron | 51838 | 51894 | + | HPODL_02016 |
| **chr1-3** | intron | 133199 | 133233 | + | HPODL_02066 |
| **chr1-3** | intron | 307989 | 308037 | + | HPODL_02177 |
| **chr1-3** | intron | 588994 | 589033 | - | HPODL_02361 |
| **chr2** | intron | 227172 | 227208 | - | HPODL_04654 |
| **chr2** | intron | 240512 | 240696 | - | HPODL_04663 |
| **chr2** | intron | 271273 | 271307 | + | HPODL_04681 |
| **chr2** | intron | 294438 | 294472 | - | HPODL_05120 |
| **chr2** | intron | 325951 | 326399 | + | HPODL_04715 |
| **chr2** | intron | 364703 | 364736 | - | HPODL_05123 |
| **chr2** | intron | 378416 | 378455 | - | HPODL_04738 |
| **chr2** | intron | 385823 | 385866 | - | HPODL_04742 |
| **chr2** | intron | 385910 | 385956 | - | HPODL_04742 |
| **chr2** | intron | 385981 | 386032 | - | HPODL_04742 |
| **chr2** | intron | 389821 | 390074 | - | HPODL_04745 |
| **chr2** | intron | 427983 | 428029 | + | HPODL_05125 |
| **chr2** | intron | 478037 | 478073 | + | HPODL_05130 |
| **chr2** | intron | 667667 | 667698 | - | HPODL_04904 |
| **chr2** | intron | 968642 | 968675 | - | HPODL_05141 |
| **chr3** | intron | 143522 | 143566 | - | HPODL_00242 |
| **chr3** | intron | 228190 | 228241 | + | HPODL_00289 |
| **chr3** | intron | 401096 | 401130 | - | HPODL_05148 |
| **chr3** | intron | 485087 | 485147 | - | HPODL_00440 |
| **chr3** | intron | 543120 | 543152 | - | HPODL_00470 |
| **chr3** | intron | 614061 | 614094 | - | HPODL_05157 |
| **chr3** | intron | 681339 | 681376 | - | HPODL_00550 |
| **chr3** | intron | 706648 | 706681 | - | HPODL_00565 |
| **chr3** | intron | 836010 | 836045 | + | HPODL_00638 |
| **chr3** | intron | 896543 | 896687 | + | HPODL_00669 |
| **chr3** | intron | 899324 | 899362 | + | HPODL_05165 |
| **chr3** | intron | 942089 | 942143 | - | HPODL_00687 |
| **chr3** | intron | 1040397 | 1040560 | + | HPODL_00751 |
| **chr3** | intron | 1217935 | 1217974 | + | HPODL_00856 |
| **chr4-1** | intron | 107918 | 107960 | + | HPODL_00953 |
| **chr4-1** | intron | 139310 | 139347 | - | HPODL_00970 |
| **chr4-1** | intron | 362523 | 362555 | - | HPODL_01092 |
| **chr4-2** | intron | 50928 | 50958 | + | HPODL_03267 |
| **chr4-2** | intron | 302766 | 302805 | - | HPODL_05194 |
| **chr4-2** | intron | 393919 | 393953 | + | HPODL_05198 |
| **chr4-2** | intron | 394085 | 394122 | + | HPODL_05198 |
| **chr4-2** | intron | 502509 | 502543 | - | HPODL_03514 |
| **chr4-2** | intron | 591645 | 591678 | - | HPODL_05210 |
| **chr4-2** | intron | 624748 | 624779 | - | HPODL_05212 |
| **chr4-2** | intron | 732835 | 732870 | - | HPODL_03653 |
| **chr4-2** | intron | 797629 | 797663 | + | HPODL_03693 |
| **chr5** | intron | 359823 | 359997 | - | HPODL_03983 |
| **chr5** | intron | 421952 | 422029 | - | HPODL_04013 |
| **chr5** | intron | 427298 | 427329 | - | HPODL_05244 |
| **chr5** | intron | 427442 | 427477 | - | HPODL_05244 |
| **chr5** | intron | 428330 | 428377 | + | HPODL_04016 |
| **chr5** | intron | 428392 | 428428 | + | HPODL_04016 |
| **chr5** | intron | 467000 | 467039 | + | HPODL_04033 |
| **chr5** | intron | 538510 | 538550 | - | HPODL_04066 |
| **chr5** | intron | 667034 | 667087 | - | HPODL_04135 |
| **chr5** | intron | 674270 | 674301 | + | HPODL_04142 |
| **chr5** | intron | 791605 | 791635 | + | HPODL_04214 |
| **chr5** | intron | 895052 | 895087 | + | HPODL_04267 |
| **chr5** | intron | 1130686 | 1130903 | + | HPODL_04408 |
| **chr5** | intron | 1215031 | 1215065 | - | HPODL_04459 |
| **chr5** | intron | 1266873 | 1266908 | + | HPODL_04489 |
| **chr5** | intron | 1277261 | 1277301 | - | HPODL_05272 |
| **chr6** | intron | 174764 | 174806 | - | HPODL_01193 |
| **chr6** | intron | 205390 | 205430 | - | HPODL_01214 |
| **chr6** | intron | 242373 | 242407 | + | HPODL_01239 |
| **chr6** | intron | 294302 | 294333 | + | HPODL_01270 |
| **chr6** | intron | 335174 | 335252 | - | HPODL_01289 |
| **chr6** | intron | 476991 | 477023 | + | HPODL_01376 |
| **chr6** | intron | 479927 | 479969 | + | HPODL_01379 |
| **chr6** | intron | 532909 | 532947 | + | HPODL_01412 |
| **chr6** | intron | 593244 | 593278 | - | HPODL_05295 |
| **chr6** | intron | 593348 | 593387 | - | HPODL_05295 |
| **chr6** | intron | 593429 | 593471 | - | HPODL_05295 |
| **chr6** | intron | 787548 | 787585 | - | HPODL_01569 |
| **chr6** | intron | 789327 | 789357 | + | HPODL_01571 |
| **chr6** | intron | 904655 | 904686 | + | HPODL_01645 |
| **chr6** | intron | 976065 | 976106 | - | HPODL_01686 |
| **chr6** | intron | 1176762 | 1176793 | - | HPODL_01799 |
| **chr6** | intron | 1348366 | 1348405 | - | HPODL_01896 |
| **chr6** | intron | 1438321 | 1438353 | - | HPODL_05323 |
| **chr7** | intron | 211767 | 211805 | + | HPODL_02508 |
| **chr7** | intron | 268958 | 268991 | - | HPODL_05332 |
| **chr7** | intron | 310198 | 310235 | - | HPODL_05334 |
| **chr7** | intron | 368899 | 368939 | - | HPODL_02592 |
| **chr7** | intron | 420539 | 420584 | + | HPODL_02623 |
| **chr7** | intron | 510872 | 510939 | - | HPODL_05338 |
| **chr7** | intron | 589021 | 589115 | - | HPODL_02702 |
| **chr7** | intron | 643221 | 643330 | + | HPODL_02737 |
| **chr7** | intron | 900381 | 900415 | - | HPODL_05356 |
| **chr7** | intron | 1111377 | 1111408 | + | HPODL_05359 |
| **chr7** | intron | 1114212 | 1114251 | - | HPODL_03019 |
| **chr7** | intron | 1168539 | 1168576 | + | HPODL_03051 |
| **chr7** | intron | 1414480 | 1414572 | - | HPODL_03187 |

**Table S12. Codon usage frequencies of protein-coding genes in *H. polymorpha* (HPOL), *D. bruxellensis* (DBRUX) and *P. pastoris* (PPAS) nuclear genomes**.

|  |  | **DBRUX** | **PPAS** | HPOL |  |  | **DBRUX** | **PPAS** | HPOL |
| --- | --- | --- | --- | --- | --- | --- | --- | --- | --- |
| Codon | AA | Fraction | Fraction | Fraction | Codon | AA | Fraction | Fraction | Fraction |
| **GCA** | A | 0,363 | 0,275 | 0,238 | **CCA** | P | 0,422 | 0,379 | 0,31 |
| **GCC** |  | 0,232 | 0,245 | 0,317 | **CCC** |  | 0,118 | 0,18 | 0,163 |
| **GCG** |  | 0,123 | 0,079 | 0,184 | **CCG** |  | 0,19 | 0,102 | 0,263 |
| **GCT** |  | 0,283 | 0,401 | 0,261 | **CCT** |  | 0,27 | 0,339 | 0,263 |
| **TGC** | C | 0,52 | 0,377 | 0,537 | **CAA** | Q | 0,438 | 0,604 | 0,377 |
| **TGT** |  | 0,48 | 0,623 | 0,463 | **CAG** |  | 0,562 | 0,396 | 0,623 |
| **GAC** | D | 0,327 | 0,383 | 0,615 | **AGA** | R | 0,399 | 0,456 | 0,348 |
| **GAT** |  | 0,673 | 0,617 | 0,385 | **AGG** |  | 0,198 | 0,181 | 0,12 |
| **GAA** | E | 0,511 | 0,595 | 0,399 | **CGA** |  | 0,082 | 0,12 | 0,094 |
| **GAG** |  | 0,489 | 0,405 | 0,601 | **CGC** |  | 0,076 | 0,051 | 0,182 |
| **TTC** | F | 0,411 | 0,422 | 0,534 | **CGG** |  | 0,123 | 0,052 | 0,136 |
| **TTT** |  | 0,589 | 0,578 | 0,466 | **CGT** |  | 0,121 | 0,141 | 0,12 |
| **GGA** | G | 0,348 | 0,364 | 0,296 | **AGC** | S | 0,121 | 0,104 | 0,179 |
| **GGC** |  | 0,202 | 0,156 | 0,344 | **AGT** |  | 0,157 | 0,159 | 0,094 |
| **GGG** |  | 0,115 | 0,122 | 0,131 | **TCA** |  | 0,212 | 0,208 | 0,122 |
| **GGT** |  | 0,335 | 0,358 | 0,229 | **TCC** |  | 0,142 | 0,174 | 0,181 |
| **CAC** | H | 0,409 | 0,383 | 0,579 | **TCG** |  | 0,153 | 0,095 | 0,23 |
| **CAT** |  | 0,591 | 0,617 | 0,421 | **TCT** |  | 0,215 | 0,26 | 0,195 |
| **ATA** | I | 0,267 | 0,236 | 0,165 | **ACA** | T | 0,356 | 0,277 | 0,239 |
| **ATC** |  | 0,269 | 0,298 | 0,442 | **ACC** |  | 0,189 | 0,238 | 0,296 |
| **ATT** |  | 0,465 | 0,466 | 0,393 | **ACG** |  | 0,179 | 0,123 | 0,249 |
| **AAA** | K | 0,462 | 0,52 | 0,465 | **ACT** |  | 0,277 | 0,362 | 0,216 |
| **AAG** |  | 0,538 | 0,48 | 0,535 | **GTA** | V | 0,093 | 0,178 | 0,058 |
| **CTA** | L | 0,095 | 0,123 | 0,083 | **GTC** |  | 0,178 | 0,217 | 0,258 |
| **CTC** |  | 0,117 | 0,084 | 0,174 | **GTG** |  | 0,305 | 0,217 | 0,412 |
| **CTG** |  | 0,076 | 0,153 | 0,298 | **GTT** |  | 0,424 | 0,388 | 0,272 |
| **CTT** |  | 0,257 | 0,171 | 0,164 | **TGG** | W | 1 | 1 | 1 |
| **TTA** |  | 0,167 | 0,178 | 0,065 | **TAC** | Y | 0,471 | 0,486 | 0,636 |
| **TTG** |  | 0,288 | 0,291 | 0,216 | **TAT** |  | 0,529 | 0,514 | 0,364 |
| **ATG** | M | 1 | 1 | 1 | **TAA** | * | 0,375 | 0,4 | 0,397 |
| **AAC** | N | 0,419 | 0,466 | 0,617 | **TAG** |  | 0,203 | 0,339 | 0,313 |
| **AAT** |  | 0,581 | 0,534 | 0,383 | **TGA** |  | 0,422 | 0,261 | 0,291 |

**Table S13. Repetitive elements in *H. polymorpha* and *P. pastoris* genomes**

|  |  | ***Hansenula polymorpha*** | | | Pichia pastoris | | |
| --- | --- | --- | --- | --- | --- | --- | --- |
|  |  | **Number of elements** | **Length occupied, bp** | **% of the genome** | **Number of elements** | **Length occupied, bp** | **% of the genome** |
| Retroelements | **SINEs:** | **0** | **0** | **0.00%** | **0** | **0** | **0.00%** |
|  | Penelope | 0 | 0 | 0.00% | 0 | 0 | 0.00% |
|  | **LINEs:** | **3** | **180** | **0.00%** | **4** | **209** | **0.00%** |
|  | CRE/SLACS | 0 | 0 | 0.00% | 0 | 0 | 0.00% |
|  | L2/CR1/Rex | 0 | 0 | 0.00% | 0 | 0 | 0.00% |
|  | R1/LOA/Jockey | 0 | 0 | 0.00% | 0 | 0 | 0.00% |
|  | R2/R4/NeSL | 0 | 0 | 0.00% | 0 | 0 | 0.00% |
|  | RTE/Bov-B | 0 | 0 | 0.00% | 0 | 0 | 0.00% |
|  | L1/CIN4 | 0 | 0 | 0.00% | 0 | 0 | 0.00% |
|  | **LTR elements:** | **70** | **22505** | **0.25%** | **45** | **2706** | **0.03%** |
|  | BEL/Pao | 0 | 0 | 0.00% | 0 | 0 | 0.00% |
|  | Ty1/Copia | 49 | 20772 | 0.23% | 13 | 831 | 0.01% |
|  | Gypsy/DIRS1 | 20 | 1673 | 0.02% | 31 | 1830 | 0.02% |
| Total retroelements | | 73 | 22685 | 0.26% | 49 | 2915 | 0.03% |
| DNA transposons | hobo-Activator | 1 | 60 | 0.00 | 2 | 107 | 0.00% |
|  | Tc1-IS630-Pogo | 7 | 338 | 0.00 | 4 | 185 | 0.00% |
|  | En-Spm | 0 | 0 | 0.00 | 0 | 0 | 0.00% |
|  | MuDR-IS905 | 0 | 0 | 0.00 | 0 | 0 | 0.00% |
|  | PiggyBac | 0 | 0 | 0.00 | 1 | 46 | 0.00% |
|  | Tourist/Harbinger | 2 | 108 | 0.00 | 2 | 128 | 0.00% |
|  | Other | 0 | 0 | 0.00 | 1 | 37 | 0.00% |
|  | Rolling-circles | 0 | 0 | 0.00 | 0 | 0 | 0.00% |
|  | Unclassified: | 1 | 85 | 0.00 | 4 | 351 | 0.00% |
| Total DNA transposons | | 16 | 987 | 0.01% | 16 | 970 | 0.01% |
| Total interspersed repeats | |  | 23757 | 0.27 |  | 4236 | 0.05% |
|  | Satellites | 0 | 0 | 0.00 | 1 | 85 | 0.00% |
|  | Simple repeats | 681 | 30130 | 0.34% | 853 | 40582 | 0.44% |
|  | Low complexity | 116 | 6215 | 0.07 | 136 | 7206 | 0.08% |

**Table S14. Estimation of genome redundancy in *H.polymorpha* and related yeasts.**

| Compared species | Duplicated 2000 bp (5000 bp) segments within genome identified by BLASTN | | | Number of fragments |
| --- | --- | --- | --- | --- |
|  | 90% | 80% | 70% |  |
| *S. cerevisiae* | 167 (64) | 209 (68) | 398 (89) | 6029 (2407) |
| *P. pastoris* | 21 (6) | 53 (10) | 103 (20) | 4607 (1841) |
| *D bruxellensis* CBS2499 | 301 (77) | 316 (77) | 376 (81) | 6646 (2644) |
| *H. polymorpha* DL-1 | 22 (10) | 29 (12) | 71 (14) | 4429 (1769) |

**Table S15. Top 11 paralogues in *H. polymorpha*, *D. bruxellensis* and *P. pastoris* genomes by gene count.**

| **OrthoMCL ID** | Count | **Gene ID** | **Function** |
| --- | --- | --- | --- |
|  |  | Dekkera bruxellensis |  |
| OG5_126579 | 11 | Dekbr2\|36211, Dekbr2\|3872, Dekbr2\|111691, Dekbr2\|50127, Dekbr2\|8081, Dekbr2\|51704, Dekbr2\|87516, Dekbr2\|13303, Dekbr2\|30383, Dekbr2\|8709, Dekbr2\|23164 | Ca2+-modulated nonselective cation channel polycystin |
| OG5_130472 | 10 | Dekbr2\|24636, Dekbr2\|45365, Dekbr2\|8847, Dekbr2\|145548, Dekbr2\|8848, Dekbr2\|8849, Dekbr2\|51825, Dekbr2\|30579, Dekbr2\|51836, Dekbr2\|8854 | alpha-glucoside permease |
| OG5_130665 | 9 | Dekbr2\|27574, Dekbr2\|23680, Dekbr2\|45121, Dekbr2\|26158, Dekbr2\|22222, Dekbr2\|49678, Dekbr2\|49789, Dekbr2\|8114, Dekbr2\|50755 | Monocarboxylate transporter |
| OG5_126661 | 7 | Dekbr2\|18416, Dekbr2\|18417, Dekbr2\|23364, Dekbr2\|4552, Dekbr2\|6459, Dekbr2\|47581, Dekbr2\|7174 | NADP-dependent alcohol dehydrogenase |
| OG5_127087 | 7 | Dekbr2\|64524, Dekbr2\|64600, Dekbr2\|64990, Dekbr2\|45870, Dekbr2\|25375, Dekbr2\|74435, Dekbr2\|51533 | Amino acid transporter |
| OG5_144544 | 7 | Dekbr2\|31638, Dekbr2\|43410, Dekbr2\|24639, Dekbr2\|56092, Dekbr2\|20019, Dekbr2\|5589, Dekbr2\|26800 | Chitinase |
| OG5_128593 | 7 | Dekbr2\|4422, Dekbr2\|63829, Dekbr2\|6884, Dekbr2\|25529, Dekbr2\|33407, Dekbr2\|48407, Dekbr2\|49744 | Monocarboxylate transporter |
| OG5_126664 | 7 | Dekbr2\|41212, Dekbr2\|42530, Dekbr2\|7314, Dekbr2\|26032, Dekbr2\|51428, Dekbr2\|67545, Dekbr2\|8840 | multidrug-resistance transporter |
| OG5_126798 | 7 | Dekbr2\|29923, Dekbr2\|25434, Dekbr2\|66929, Dekbr2\|8101, Dekbr2\|8102, Dekbr2\|8103, Dekbr2\|26639 | glucose transporter |
| OG5_126583 | 6 | Dekbr2\|3893, Dekbr2\|63666, Dekbr2\|47567, Dekbr2\|7973, Dekbr2\|26718, Dekbr2\|26576 | Aldo/keto reductase |
| OG5_126560 | 6 | Dekbr2\|23596, Dekbr2\|20645, Dekbr2\|6903, Dekbr2\|7376, Dekbr2\|114128, Dekbr2\|26762 | LAR-interacting protein |
|  |  | Hansenula polymorpha |  |
| OG5_132365 | 9 | HPODL_02104, HPODL_04840, HPODL_01489, HPODL_01382, HPODL_01624, HPODL_01625, HPODL_02407, HPODL_02408, HPODL_03209 | alpha 1,6-mannosyltransferase |
| OG5_126583 | 8 | HPODL_00887, HPODL_00934, HPODL_03756, HPODL_03784, HPODL_05275, HPODL_01184, HPODL_01370, HPODL_02752 | Aldo/keto reductase |
| OG5_134531 | 8 | HPODL_02296, HPODL_04819, HPODL_03547, HPODL_03663, HPODL_01623, HPODL_01669, HPODL_01927, HPODL_05372 | Alpha-1,2-mannosyltransferase |
| OG5_126647 | 7 | HPODL_00034, HPODL_00047, HPODL_04530, HPODL_00586, HPODL_00646, HPODL_00972, HPODL_02813 | Uncharacterized transporter |
| OG5_126661 | 6 | HPODL_00654, HPODL_03666, HPODL_02398, HPODL_02406, HPODL_02410, HPODL_02528 | NADP-dependent alcohol dehydrogenase |
| OG5_128108 | 6 | HPODL_00002, HPODL_02375, HPODL_02392, HPODL_00213, HPODL_00916, HPODL_01745 | 5-oxoprolinase |
| OG5_132166 | 5 | HPODL_03754, HPODL_01659, HPODL_01917, HPODL_01918, HPODL_01919 | alpha 1,2-mannosyltransferase |
| OG5_126561 | 5 | HPODL_02289, HPODL_04837, HPODL_04937, HPODL_04179, HPODL_04463 | ATP-dependent bile acid permease |
| OG5_127267 | 5 | HPODL_00001, HPODL_00340, HPODL_00864, HPODL_00889, HPODL_04523 | oxidoreductase |
| OG5_128593 | 5 | HPODL_02159, HPODL_05132, HPODL_04430, HPODL_04431, HPODL_04432 | Monocarboxylate transporter |
| OG5_144599 | 5 | HPODL_02117, HPODL_04684, HPODL_04866, HPODL_00873, HPODL_04451 | hypothetical protein |
|  |  | ***Pichia pastoris*** |  |
| OG5_127062 | 7 | gi\|254573296\|ref\|XP_002493757.1\|, gi\|254573296\|ref\|XP_002493757.1\|, gi\|254571131\|ref\|XP_002492675.1\|, gi\|254571129\|ref\|XP_002492674.1\|, gi\|254571127\|ref\|XP_002492673.1\|, gi\|254571125\|ref\|XP_002492672.1\|, gi\|254570229\|ref\|XP_002492224.1\| | Putative dihydrokaempferol 4-reductase |
| OG5_179559 | 7 | gi\|254572606\|ref\|XP_002493412.1\|, gi\|254570213\|ref\|XP_002492216.1\|, gi\|254570206\|ref\|XP_002492213.1\|, gi\|254567555\|ref\|XP_002490888.1\|, gi\|254567229\|ref\|XP_002490725.1\|, gi\|254567217\|ref\|XP_002490719.1\|, gi\|254564465\|ref\|XP_002489343.1\| | hypothetical protein |
| OG5_127087 | 6 | gi\|254568926\|ref\|XP_002491573.1\|, gi\|254567259\|ref\|XP_002490740.1\|, gi\|254567003\|ref\|XP_002490612.1\|, gi\|254565779\|ref\|XP_002490000.1\|, gi\|254565483\|ref\|XP_002489852.1\|, gi\|254565109\|ref\|XP_002489665.1\| | Amino acid transporter |
| OG5_126574 | 6 | gi\|254574342\|ref\|XP_002494280.1\|, gi\|254569556\|ref\|XP_002491888.1\|, gi\|254568128\|ref\|XP_002491174.1\|, gi\|254567936\|ref\|XP_002491078.1\|, gi\|254567533\|ref\|XP_002490877.1\|, gi\|254565891\|ref\|XP_002490056.1\| | Plasma membrane ATP binding cassette (ABC) transporter |
| OG5_137281 | 6 | gi\|254573826\|ref\|XP_002494022.1\|, gi\|254571979\|ref\|XP_002493099.1\|, gi\|254570827\|ref\|XP_002492523.1\|, gi\|254570819\|ref\|XP_002492519.1\|, gi\|254570525\|ref\|XP_002492372.1\|, gi\|254565855\|ref\|XP_002490038.1\| | Aspartic protease |
| OG5_126641 | 6 | gi\|254572806\|ref\|XP_002493512.1\|, gi\|254565519\|ref\|XP_002489870.1\|, gi\|254565457\|ref\|XP_002489839.1\|, gi\|254572806\|ref\|XP_002493512.1\|, gi\|254565519\|ref\|XP_002489870.1\|, gi\|254565457\|ref\|XP_002489839.1\| | 60S ribosomal protein L2 |
| OG5_130254 | 5 | gi\|254573078\|ref\|XP_002493648.1\|, gi\|254573438\|ref\|XP_002493828.1\|, gi\|254573078\|ref\|XP_002493648.1\|, gi\|254570923\|ref\|XP_002492571.1\|, gi\|254567635\|ref\|XP_002490928.1\| | Ferric reductase |
| OG5_126561 | 5 | gi\|254572966\|ref\|XP_002493592.1\|, gi\|254572964\|ref\|XP_002493591.1\|, gi\|254567295\|ref\|XP_002490758.1\|, gi\|254567175\|ref\|XP_002490698.1\|, gi\|254565521\|ref\|XP_002489871.1\| | ATP-dependent bile acid permease |
| OG5_129349 | 5 | gi\|254568928\|ref\|XP_002491574.1\|, gi\|254565933\|ref\|XP_002490077.1\|, gi\|254565931\|ref\|XP_002490076.1\|, gi\|254565853\|ref\|XP_002490037.1\|, gi\|254565385\|ref\|XP_002489803.1\| | Putative transmembrane protein |
| OG5_126661 | 4 | gi\|254573810\|ref\|XP_002494014.1\|, gi\|254570215\|ref\|XP_002492217.1\|, gi\|254568544\|ref\|XP_002491382.1\|, gi\|254565807\|ref\|XP_002490014.1\| | NADP-dependent alcohol dehydrogenase |
| OG5_126687 | 4 | gi\|254572507\|ref\|XP_002493363.1\|, gi\|254567029\|ref\|XP_002490625.1\|, gi\|254572507\|ref\|XP_002493363.1\|, gi\|254567029\|ref\|XP_002490625.1\| | mitochondrial 37S ribosomal protein |

# Table S16. Fungal and yeast species used in phylogenetic analysis­­­­­

| **Ascomycetes species** | **BioProject** |
| --- | --- |
| *Ajellomyces capsulatus* NAm1 | PRJNA20041 |
| *Ashbya gossypii* ATCC 10895 | PRJNA10623 |
| *Aspergillus clavatus* NRRL 1 | PRJNA18467 |
| *Aspergillus flavus* NRRL3357 | PRJNA38227 |
| *Aspergillus fumigatus* A1163 | PRJNA18733 |
| *Aspergillus nidulans* FGSCA4 | PRJNA13961 |
| *Aspergillus niger* CBS 513.8 | PRJNA19263 |
| *Aspergillus oryzae* RIB40 | PRJNA28175 |
| *Aspergillus terreus* NIH2624 | PRJNA17637 |
| *Botryotinia fuckeliana* T4 | PRJNA20061 |
| *Candida albicans* SC5314 | PRJNA14005 |
| *Candida dubliniensis* CD36 | PRJNA38659 |
| *Candida glabrata* CBS138 | PRJNA12376 |
| *Candida tropicalis* MYA-3404 | PRJNA39569 |
| *Chaetomium globosum* CBS 148 | PRJNA16821 |
| *Clavispora lusitaniae* ATCC 42720 | PRJNA41079 |
| *Debaryomyces hansenii* CBS7657 | PRJNA12410 |
| *Dekkera bruxellensis* AWRI1499 | PRJNA78661 |
| *Dekkera bruxellensis* CBS 2499 | PRJNA76499 |
| *Exophiala dermatitidis* NIH UT8656 | PRJNA64935 |
| *Fusarium pseudograminearum* CS3096 | PRJNA66583 |
| *Gibberella zeae* PH-1 | PRJNA243 |
| *Grosmannia clavigera* kw1407 | PRJNA39837 |
| *Hansenula polymorpha* DL-1 | This study |
| *Kluyveromyces lactis* NRRL Y-1140 | PRJNA12377 |
| *Komagataella pastoris* GS115 | PRJNA39439 |
| *Lachancea thermotolerans* 6340 | PRJNA39575 |
| *Leptosphaeria maculans* JN3 | PRJNA171003 |
| *Lodderomyces elongisporus* NRRL YB-4239 | PRJNA19611 |
| *Magnaporthe oryzae* 70-15 | PRJNA1433 |
| *Marssonina brunnea* MB-m1 | PRJNA66127 |
| *Meyerozyma guilliermondii* ATCC 6260 | PRJNA19593 |
| *Naumovozyma castellii* CBS 4309 | PRJNA79343 |
| *Neosartorya fischeri* NRRL 181, 79 | PRJNA18475 |
| *Neurospora crassa* OR74A | PRJNA132 |
| *Penicillium chrysogenum* Wisconsin 54-1255 | PRJNA39879 |
| *Phaeosphaeria nodorum* SN15 | PRJNA21049 |
| *Podospora anserina* S mat+ | PRJNA29799 |
| *Saccharomyces cerevisiae* S288c | PRJNA128 |
| *Scheffersomyces stipitis* CBS 6054 | PRJNA18881 |
| Schizosaccharomyces pombe | PRJNA127 |
| *Sclerotinia sclerotiorum* 1980 UF-70 | PRJNA20263 |
| *Vanderwaltozyma polyspora* DSM70294 | PRJNA20539 |
| *Yarrowia lypolitica* CLIB122 | PRJNA12414 |
| *Zygosaccharomyces rouxii* CBS732 | PRJNA39573 |
| *Zymoseptoria tritici* IPO323 | PRJNA170847 |

1. Orthologous to *P.pastoris* Mxr1p, *C.boidinii* Trm2p, *S.cerevisiae*  Adr1p [↑](#footnote-ref-1)
2. Orthologous to *C.boidinii* Trm1p [↑](#footnote-ref-2)
3. Orthologous to *S.cerevisiae* CAT8p [↑](#footnote-ref-3)
