# Supplementary material for: Genome sequence and analysis of methylotrophic yeast Hansenula polymorpha DL1
Source: BMC Genomics. 2013 Nov 27;14:837. doi: 10.1186/1471-2164-14-837 (PMC3866509; doi:10.1186/1471-2164-14-837)
Supplement: Additional file 3 — Validation of RNA-seq data by quantitative PCR. [file 1471-2164-14-837-S3.docx]

Validation of RNA-seq data by quantitative PCR

In order to validate RNA-seq data and evaluate reproducibility of observed patterns of expression, we performed qPCR analysis of transcription of three selected genes differently expressed in glucose and methanol. Gene HPODL_1177 showed approximately equal expression on methanol and on glucose, HPODL_1513 is 2,6 times downregulated by methanol, and HPODL_2458 is 4,7 times induced on methanol according to RNA-seq data. New cultures, other than ones used for RNA-seq, were prepared for these experiments.

For qPCR analysis *H. polymorpha* DL-1 was grown up to OD_660_ ~2.2 in 0.67% YNB medium containing leucine (20 mg/l) and either 1% glucose or 1% methanol at 37°C while shaking at 250 rpm. Two independent cultures grown in medium with methanol and two cultures grown in medium with glucose were prepared. Cells were harvested by centrifugation (4000 rpm, 10 min, 4°C) and taken up in AE-buffer (50mM sodium acetate, 10mM EDTA, pH 5.0). The total RNA was extracted by a hot phenol method followed by purification using RNeasy Mini Kit (Qiagen).

Total RNA samples were used for cDNA synthesis employing RevertAid First Strand cDNA Synthesis Kit (Thermo Scientific) with either oligo-dT or random hexamer primers. qPCR analysis of cDNA samples was performed using SsoFast EvaGreen Supermix (BioRad) and primers shown below. As an additional control we used cDNA samples analysed in RNA-seq experiments.

HPODL_1177_F TTTGGTCATTGGAGGAGGTTCTG

HPODL_1177_R GTACCACATCACTTTCTTTGGAACA

HPODL_1513_F CAAACTCGGATGGCTTACCAG

HPODL_1513_R GATGTTCAAGCCTGCCTTTCTG

HPODL_2458_F CCAGTCAGATCTGCACTGAATG

HPODL_2458_R AACTCATTGATCTGGCCAGAAATG

Table 1. Differential expression of genes HPODL_1177, HPODL_1513, and HPODL_2458

| Sample and approach used | Relative expression level (LOG2 M/G) | | |
| --- | --- | --- | --- |
|  | HPODL_1177 | HPODL_1513 | HPODL_2458 |
| RNA-seq | 0,25 | -1,36 | 2,23 |
| qPCR on cDNA used in RNA-seq experiment | 0,36 | -1,39 | 2,32 |
| qPCR on cDNAs synthesized using oligo-dT (new cultures) | 0,29±0,06 | -2,74±0,13 | 1,99±0,09 |
| qPCR on cDNAs synthesized using random primer (new cultures) | 0,38±0,02 | -2,44±0,12 | 2,29±0,13 |

The data presented in Table 1 show that, in spite of some variability associated with different techniques used, the patterns of regulation are reproducible.
