# Supplementary material for: Genome sequence and analysis of methylotrophic yeast Hansenula polymorpha DL1
Source: BMC Genomics. 2013 Nov 27;14:837. doi: 10.1186/1471-2164-14-837 (PMC3866509; doi:10.1186/1471-2164-14-837)
Supplement: Additional file 4 — Confirmation of alternative splicing events. [file 1471-2164-14-837-S4.docx]

Confirmation of alternative splicing events

In order to confirm alternative splicing events, predicted by transcriptome sequencing, we analysed the structure of transcripts by PCR. Two genes were selected for such analysis, HPODL_03187 and HPODL_04408. In both cases mapping of RNA-seq reads indicated approximately equal amounts of two transcripts – correctly spliced variant and the transcript with retained intron. cDNA sample of *H. polymorpha* DL-1 grown with methanol was used as a template for PCR amplification with exon-specific primers, flanking the predicted intron. Primers 3187_F (TCC CCA ACT GCT GTC AAT TAA AAG T) and 3187_R (TGG TTG CTT GTT GTG CTC AGC) were used for gene HPODL_03187 cDNAs, and primers 4408_F (ATG TTA GTG ACT GGA GTT GCG ATT) and 4408_R (TCC AGA AAT GGT GTA CAC CAA G) – for gene HPODL_04408. The sizes of PCR fragments corresponding to “retained intron” variant and correctly spliced transcripts of genes HPODL_03187 and HPODL_04408, as predicted by RNA-seq, are 306/215 bp and 416/200 bp, respectively.

Results of gel electrophoresis analysis of transcripts are shown in Figure 1. In both cases we observed two fragments with lengths corresponding to full size (retained intron) and spliced variants of the transcript. Sequencing of these fragments confirmed positions of splice sites.


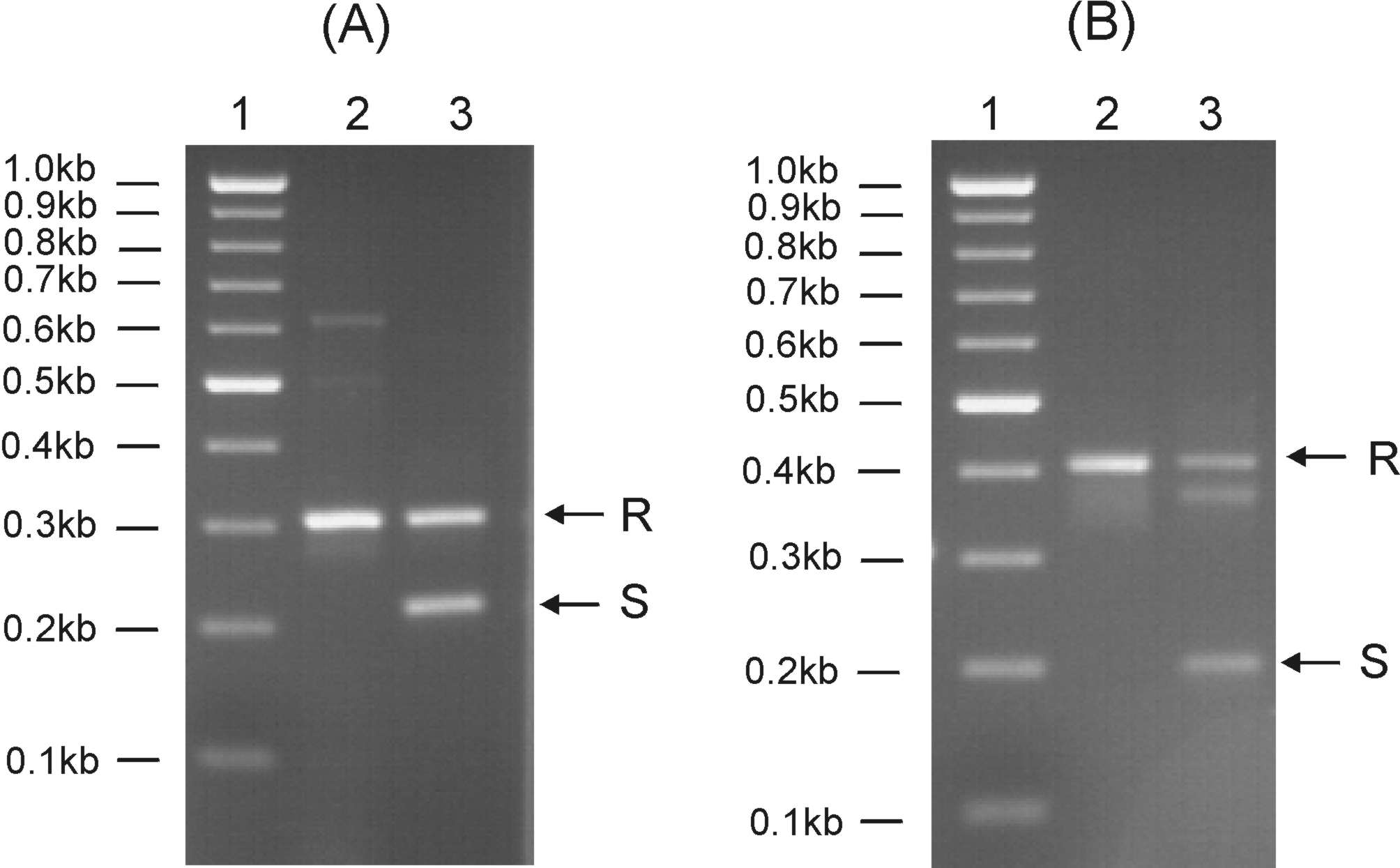


Figure 1. Alternative splicing of transcripts of genes HPODL_3187 (A) and HPODL_4408 (B).

1, - molecular weight markers,

2, - PCR fragments obtained on genomic DNA as a template,

3, - PCR fragments obtained on cDNA

Fragments representing retained intron (R) and spliced transcript (S) are shown.

Note that fragment migrating slightly faster than fragment “R” in panel B represents hybrid resulting from annealing of R and S strands, as revealed by sequencing.
